# S100A11 regulates hepatic cholesterol metabolism and promotes steatohepatitis via noncanonical SREBP2 signaling

**DOI:** 10.1172/JCI201406

**Published:** 2026-08-04

**Authors:** Mingfeng Zhan, Xiumei Xu, Huiyin Wu, Qijing Fan, Hongsheng Lu, Chengbin Li, Linqiang Zhang, Tingting Zhu, Yunqian Shen, Jing Liu, Yaomei He, Yingjie Wu, Jingjing Zhang, Xiaoju Zou, Bin Liang

**Affiliations:** 1 School of Life Sciences, Yunnan Key Laboratory of Cell Metabolism and Diseases, Yunnan University, Kunming, China.; 2 College of Chinese Materia Medica and Yunnan Key Laboratory of Southern Medicinal Utilization, Yunnan University of Chinese Medicine, Kunming, Yunnan, China.; 3 Southwest United Graduate School, Kunming, China.; 4 Department of Hepatobiliary and Pancreatic Surgery, the Second Affiliated Hospital of Guangdong Medical University, Zhanjiang, Guangdong, China.; 5 Institute of Life Sciences, School of Basic Medicine, Chongqing Medical University, Chongqing, China.; 6 School of Medicine, Guizhou University, Guiyang, Guizhou, China.; 7 Institute for Genome Engineered Animal Models of Human Diseases, National Center of Genetically Engineered Animal Models for International Research, Liaoning Provence Key Lab of Genome Engineered Animal Models, Dalian Medical University, Dalian, Liaoning, China.

**Keywords:** Hepatology, Inflammation, Cholesterol, Hepatitis, Homeostasis

## Abstract

Dietary cholesterol and de novo cholesterol synthesis in the liver use reciprocal coordination to maintain cholesterol homeostasis. However, high levels of dietary cholesterol still promote excessive cholesterol accumulation in the liver, leading to metabolic dysfunction–associated steatohepatitis (MASH), yet the mechanisms remain poorly understood. Here, we show that hepatic S100 calcium-binding protein A11 (S100A11), a member of the S100 family of calcium-binding proteins, positively responded to dietary cholesterol levels and was involved in hepatic cholesterol metabolism. S100A11 localized to the ER and could bind to cholesterol. In vivo and in vitro, hepatic overexpression of S100A11 led to sterol regulatory element–binding transcription factor 2 (SREBP2) activation to promote cholesterol synthesis, uptake, and accumulation, consequently exacerbating steatohepatitis. In contrast, inactivation of S100A11 had opposite effects and improved steatohepatitis. Mechanistically, S100A11 triggered the noncanonical entry of SREBP2 into the nucleus through a S100A11/annexin A1/importin β (S100A11/ANXA1/KPNB) axis, distinct from the well-known insulin-induced gene /SREBP cleavage-activating protein or caspase 2 pathways. Therefore, our work identifies S100A11 as a regulator of liver cholesterol metabolism, providing a promising target to treat MASH and hypercholesterolemia.

## Abstract 2

This study reveals that S100A11 is a previously unreported key regulator of cholesterol metabolism, suggesting the potential value of S100A11 as a biomarker for MASH.

## Graphical abstract

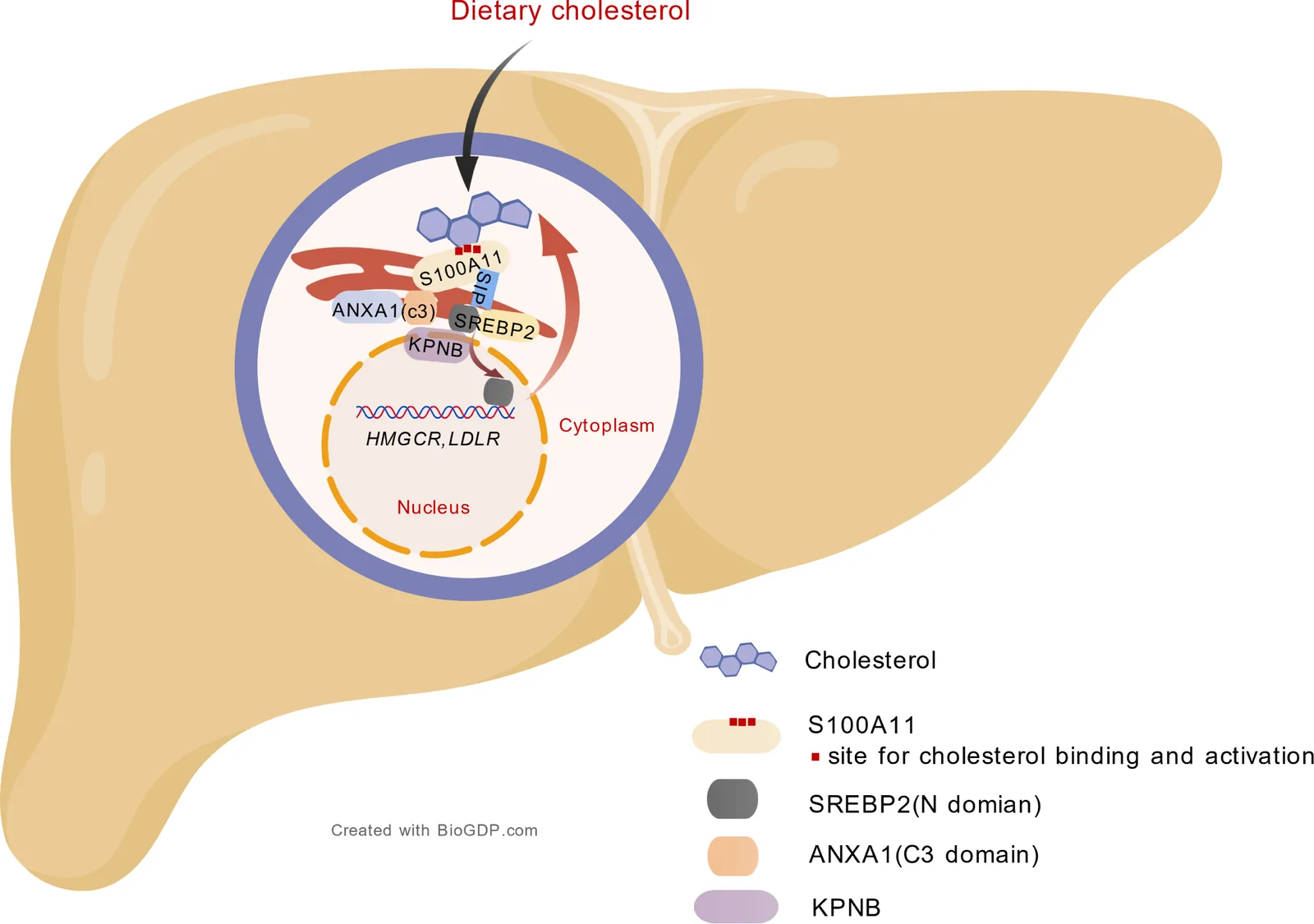

## Introduction

Cholesterol is an essential component of eukaryotic cells, and it plays vital roles in numerous biological processes. Either a deficiency or, in particular, an excess of cholesterol can be harmful to the body, leading to a variety of severe diseases, including atherosclerosis, cardiovascular disease, metabolic dysfunction–associated steatotic liver disease (MASLD, formerly called NAFLD), and others (1–3). The liver is the crucial organ in regulating cholesterol homeostasis, since the majority of cholesterol synthesis occurs within this organ (4). Increased cholesterol synthesis and accumulation in the liver correlates with the severity of metabolic dysfunction–associated steatohepatitis (MASH) (5), an advanced stage of MASLD. Therefore, animals must possess strict and finely tuned regulatory mechanisms to maintain liver cholesterol homeostasis.

In general, cholesterol homeostasis in the liver is tightly regulated in several ways, including de novo synthesis, exogenous absorption and transportation, and transformation and efflux (2, 3, 6). Correspondingly, the liver has evolved multiple mechanisms to regulate these processes in response to cholesterol levels. When the intracellular sterol content is insufficient, the sterol regulatory element–binding transcription factor 2 (SREBP2), a master regulator of hepatic cholesterol synthesis, is activated to transactivate genes involved in cholesterol synthesis via a negative feedback mechanism (3, 7–9). On the other hand, when cellular cholesterol levels become excessive, other factors, such as liver X receptor α (LXRA) and nuclear respiratory factor 1 (NRF1) (10–12), activate the expression of a series of genes related to cholesterol efflux, transport, and excretion, thereby reducing hepatic cholesterol content and maintaining homeostasis. Dysfunction of either LXRA (10) or NRF1 (11) in mice further leads to exacerbated hepatic cholesterol accumulation under the challenge of a high-cholesterol diet.

In the human body, the amount of cholesterol from endogenous biosynthesis versus dietary absorption is approximately 7:3 (2, 4, 13). A reciprocal coordination between dietary cholesterol absorption and de novo cholesterol synthesis in the liver is crucial to maintain cholesterol homeostasis. Increased cholesterol synthesis is correlated with decreased cholesterol absorption (14). Dietary cholesterol intake activates the cholesin/GPR146 axis, which suppresses hepatic SREBP2 to reduce cholesterol synthesis in the liver (15). In contrast, some existing studies from human and animal models have found that hepatic cholesterol content and MASH development are positively correlated with dietary cholesterol intake (16–20). The activity of SREBP2 and its involvement downstream with the cholesterol synthesis–related enzyme 3-hydroxy-3-methyl-glutaryl-coenzyme A reductase (HMGCR) is enhanced in clinical samples with increased hepatic cholesterol accumulation (5, 17). In some diet-induced mouse models of MASLD/MASH, a high-fat, high-cholesterol (HFHC) diet is still able to promote hepatic SREBP2 expression and cholesterol accumulation, exacerbating MASH. For example, feeding a HFHC diet to *foz/foz* mice results in increased SREBP2 expression and cholesterol accumulation in the liver, which subsequently exacerbates the course of MASH (21). These studies suggest that the regulation of hepatic cholesterol homeostasis is much more complicated than what we have understood so far, and, consequently, there may be other factors in response to excessive cholesterol to upregulate hepatic cholesterol accumulation and MASH development.

Therefore we analyzed integrated data from genes or proteins in response to excessive cholesterol diets in different animal models of MASH. S100A11, a member of the S100 family of calcium-binding proteins, was identified as a cholesterol sensor, whose expression was positively regulated by cholesterol levels. Both in vivo and in vitro, S100A11 contributed to hepatic cholesterol accumulation by prompting the nuclear translocation of SREBP2 through a S100A11/annexin A1/importin β (S100A11/ANXA1/KPNB) axis. Inactivation of S100A11 in hepatocytes reduced cholesterol accumulation and, subsequently, inflammation, and thus alleviated MASH and hypercholesterolemia.

## Results

### Identification of S100A11 in the liver in response to high dietary cholesterol.

In order to search for potential factors in the liver in response to high dietary cholesterol, we screened differentially expressed genes (DEGs) by coanalyzing published gene set enrichment (GSE) data from several MASH models of distinct organisms including mice, Tibetan pig, Olive baboon, and rabbits under cholesterol induction (Figure 1A and Supplemental Table 1; supplemental material available online with this article; https://doi.org/10.1172/JCI201406DS1). A total of 7 genes showed consistent upregulation in mice in the GSE data, namely *S100A11*, *ANXA2*, *LGALS3*, *LYZ2*, *CLEC7A*, *LPL*, and *SERPINA12* (Figure 1B, left pane, and Supplemental Table 2). Furthermore, intersection results demonstrated that only *S100A11* (S100 calcium-binding protein A11) was consistently upregulated among these DEGs from multiple species (Figure 1B, right panel). Moreover, *S100A11* upregulation was also detected in the liver of patients with MASH (Figure 1, C–E). Taken together, these lines of evidence imply that S100A11 may respond to high cholesterol levels and be involved in hepatic cholesterol metabolism.

To explore the relationship between S100A11 and cholesterol levels, different amounts of cholesterol were added to the hepatocyte cell lines HepG2, Huh7, or Hepa1–6. Western blot analysis showed that the protein level of S100A11 was elevated by cholesterol in a dose-dependent manner (Figure 1, F–H). We generated a cell line with stable expression of S100A11-RFP, and it showed that the fluorescence expression of S100A11-RFP was activated by cholesterol and repressed by lipid-depleted serum (LDS), which we used to establish a low-cholesterol cell culture environment (Figure 1I). S100A11 is expressed less in the liver of mice and humans when compared with other tissues and organs (22). We therefore fed mice diets containing varying concentrations of cholesterol for different durations. Both longer high-cholesterol diet feeding duration and higher dietary cholesterol levels resulted in upregulated S100A11 expression (Figure 1, J–L). Collectively, these results demonstrate that S100A11 is a potential player in the cellular response to cholesterol.

### S100A11 has the ability to bind to cholesterol.

Since the expression of S100A11 is regulated by cholesterol, we next examined whether S100A11 can bind to cholesterol. The currently available results of the structural elucidation of S100A11 indicate that S100A11 is a calcium-binding protein containing 2 EF-hand motifs, and it is usually present as homodimers and heterodimers in specific cell types (23–26). Although the structure of S100A11 is symmetric, we gave each monomer a different color for easy identification (Figure 2A). AlphaFold structure prediction showed that each monomer of the recombinant human S100A11 protein dimer consists of 5similar helices (H1–H5), 2 β-strands (B1: AA33-35; B2: AA74-76), and intrinsically disordered regions (IDRs) (Figure 2A). The 2 EF-hand motifs, EF-hand 1 (AA13-49) contain parts of H1, B1, H2, and part of H3, whereas EF–hand 2 (AA55-90) contains H4 and part of H5. Through molecular docking prediction, we identified several potential binding sites between S100A11 and cholesterol, with the 4 amino acids N82, G86, K36, and D61 exhibiting the highest binding energies (Figure 2, B and C).

In order to test whether S100A11 could directly bind to cholesterol, we performed membrane lipid strip assays. The results showed that S100A11 indeed exhibited in vitro binding to cholesterol (Figure 2D). In addition, it also bound to other lipids such as phosphatidic acid (PA), phosphatidylserine (PS), and cardiolipin (CL) (Figure 2D). However, unlike cholesterol (Figure 1, F–H), the other 3 available lipids — PA, PS, and CL — could not activate S100A11 expression (Supplemental Figure 1A), suggesting that S100A11 may specifically respond to cholesterol. In Huh7 cells, S100A11 could colocalize with cholesterol in the ER using the S100A11 antibody and the ER markers calnexin (Figure 2E) and binding immunoglobulin protein (BIP) (Supplemental Figure 1B) with filipin used to stain free cholesterol (FC). Next, to examine whether S100A11 could respond to cholesterol in the ER, we applied U18666A, which is a cholesterol transporter inhibitor that specifically inhibits the transfer of exogenous cholesterol into the ER (27). Application of U18666A blocked the increased expression of S100A11 triggered by cholesterol in both Huh7 and Hepa1–6 cell lines (Figure 2, F and G), suggesting that S100A11 may be directly regulated by cholesterol in the ER.

Next, to test whether the potential binding sites in S100A11 are required for its activation, we constructed N- and C-terminal deletion mutants and induced expression of the N- and C-terminal domains of S100A11 alone. These constructs were fused with GFP and transfected into Huh7 cells (Figure 2H). Both Western blot analysis and fluorescence microscopy revealed that the expression of S100A11(ΔN) and S100A11(ΔC) were activated by cholesterol, similar to what was observed in full-length S100A11 (Supplemental Figure 1, C and D). In contrast, neither S100A11(N) nor S100A11(C) responded to cholesterol (Supplemental Figure 1, C and D), suggesting that the region from AA23 to AA86 is essential for S100A11 activation by cholesterol. This region (AA23-86) contains the 4 potential cholesterol-binding sites N82, G86, K36, and D61. We therefore generated 4 truncations with the corresponding point mutations N82G, G86T, K36I, and D61G, respectively. Notably, all mutant proteins no longer responded to cholesterol (Figure 2, I and J). Moreover, MicroScale thermophoresis (MST) analysis further confirmed the binding of S100A with cholesterol, while the three truncations N82G, G86T, and K36I lost its binding activity (Figure 2K), suggesting that these 3 sites are essential and necessary for cholesterol binding and activation of S100A1. Altogether, these lines of evidences demonstrate that S100A11 is a cholesterol sensor that can physically bind to cholesterol and is activated by cholesterol.

### S100A11 promotes hepatic cholesterol accumulation via the SREBP2 pathway.

To explore how S100A11 is involved in cholesterol metabolism, stable *S100A11*-overexpressing (*S100A11*-OE) and *S100A11*-knockdown cell lines in both Huh7 and Hepa1–6 were constructed (Figure 3, A and B). Compared with the control cells, *S100A11*-OE cells accumulated increased FC in both cell lines, whereas knockdown of *S100A11* decreased the FC level (Figure 3, C and D), suggesting that S100A11 is involved in hepatic cholesterol metabolism.

Cholesterol accumulation in the liver may be caused by a variety of factors, including cholesterol synthesis, uptake, and efflux pathways (1). The sterol regulatory element–binding transcription factor 2 (SREBP2) is a master regulator of hepatic cholesterol homeostasis (3, 7–9). Lovastatin is a classical inhibitor of the cholesterol synthesis pathway (28). Application of lovastatin to either the control cell line or the *S100A11*-OE cell line led to a similar trend in the reduction of intracellular cholesterol content (Figure 3, E and F). Furthermore, Western blot analysis showed that overexpression of *S100A11* increased protein levels of the total (P) and nuclear (N) forms of SREBP2 as well as its downstream targets 3-hydroxy-3-methylglutaryl-CoA reductase (HMGCR), a rate-limiting enzyme in cholesterol synthesis, and LDL receptor (LDLR), which regulates liver cholesterol uptake (Figure 3, G and H). In contrast, knockdown of *S100A11* displayed completely opposite effects in both cell lines (Figure 3, I and J). Furthermore, immunofluorescence staining supported that SREBP2 was at a higher level in the nucleus when *S100A11* was overexpressed (Figure 3K). Taken together, these results indicate that accumulation of cholesterol promoted by S100A11 may be mediated by SREBP2-regulated cholesterol synthesis or uptake.

### Liver-specific overexpression of S100A11 promotes SREBP2 expression and hepatic cholesterol accumulation.

To better understand the role of S100A11 in hepatic cholesterol metabolism, liver-specific *S100A11*-OE mice (*S100A11^HepKI^*, simplified as *Hep*-KI) were generated (Supplemental Figure 2, A–C). The HFHC diet has been widely used to promote hepatic cholesterol accumulation that consequently triggers the development of MASH (2, 6, 29–31) and also activates SREBP2 and cholesterol accumulation in the liver of *foz/foz* mice (21). Therefore, we subjected *Hep*-KI mice, in parallel with their corresponding controls (*S100A11^HepROSA^*, *HepROSA*), to a HFHC diet for 20 weeks (Figure 4A).

Compared with the control mice, *Hep*-KI mice displayed increased plasma levels of alanine aminotransferase (ALT) and aspartate aminotransferase (AST) (Supplemental Figure 2, D and E), which are indicators of exacerbated liver inflammation and injury. Body weight, along with plasma levels of triglyceride (TG) and HDL cholesterol (HDL-c) showed no obvious change (Supplemental Figure 2, F–H). More important, the plasma level of total cholesterol (TC) and LDL cholesterol (LDL-C) as well as liver TC and TG levels were substantially increased (Figure 4, B–E). Furthermore, liver sections from *Hep*-KI mice also showed a substantial increase in steatosis by H&E staining (Figure 4F) and FC by filipin staining (Figure 4G), as well as increased F4/80 IHC staining and collagen deposition, as indicated by Picrosirius red staining (Figure 4F). These data suggest that overexpression of S100A11 in the liver promoted hypercholesterolemia, hepatic cholesterol accumulation, and inflammation.

Consistent with the in vitro results (Figure 3, G and H), overexpression of *S100A11* promoted the expression (total [P]) and nuclearization (nuclear [N]) of SREBP2 and its downstream targets HMGCR and LDLR (Figure 4H). Similarly, IHC analysis also confirmed their upregulated expression (Figure 4I) and increased nuclear localization of SREBP2 (Figure 4J). Excessive cholesterol is able to activate the expression of inflammatory factors including IL-1B, IL-1B–p17, and caspase 1–p20, ultimately exacerbating inflammation (32, 33). Indeed, overexpression of *S100A11* promoted the expression of these inflammatory factors (Figure 4K), indicating increased inflammation. Taken together, these results, in line with the in vitro data, consistently suggested that overexpression of *S100A11* in the liver activated SREBP2-mediated hepatic cholesterol accumulation to aggravate inflammation and MASH.

### Liver-specific KO of S100A11 reduces SREBP2 expression and hepatic cholesterol accumulation.

Next, to determine whether disruption of S100A11 in the liver could have beneficial effects, we generated liver-specific *S100A11*-KO mice (*S100A11^HepKO^*, simplified as *Hep*-KO) (Supplemental Figure 3, A–C). These mice were similarly subjected to a HFHC diet for 20 weeks, in parallel with their corresponding controls (*S100A11^F/F^*, simplified as *F/F*) (Figure 5A). Compared with the control mice, *Hep*-KO mice had reduced plasma levels of AST, ALT, and HDL-c, with plasma TG levels and body weight remaining unchanged (Supplemental Figure 3, D–H). Their plasma levels of TC and LDL-C, as well as liver TC and TG levels, were substantially decreased (Figure 5, B–E). Additionally, liver sections from *Hep*-KO mice also showed substantially reduced F4/80 IHC staining as well as collagen deposition, as indicated by Picrosirius red staining (Figure 5F), suggesting alleviated hepatic inflammation by KO of *S100A11* in the liver. Moreover, the livers of these mice showed substantially reduced steatosis by H&E staining (Figure 5F) and FC by filipin staining (Figure 5G). Collectively, these data suggest that hepatic dysfunction of S100A11 has the ability to reduce liver cholesterol accumulation and inflammation.

Similar to the in vitro results (Figure 3, I and J), Western blot analysis showed that liver KO of *S100A11* attenuated SREBP2 expression (P) and nuclear localization (N), as well as the expression of its downstream targets HMGCR and LDLR (Figure 5H). These data were also confirmed by IHC analysis (Figure 5I). Furthermore, *S100A11* KO reduced the expression of inflammatory factors including IL-1B, IL-1B–p17, and caspase 1–p20 (Figure 5J). Altogether, the above data demonstrate that disruption of S100A11 can reduce hypercholesterolemia and liver cholesterol accumulation, thereby alleviating MASH.

### S100A11 activation of SREBP2 is independent of the insulin-induced gene /SREBP cleavage-activating protein or caspase 2 pathway.

The in vivo and in vitro data previously shown consistently uncovered an uncharacterized role of S100A11 in regulating SREBP2-mediated cholesterol metabolism, but the underlying mechanism was unclear. The well-established negative feedback mechanism in the SREBP2/cholesterol pathway is mainly maintained by insulin-induced gene 1 (INSIG) and SREBF chaperone (SCAP) proteins (7). However, expression levels of INSIG and SCAP proteins were not changed in the livers of either *S100A11*-OE mice (Supplemental Figure 4A) or KO mice (Supplemental Figure 4B) when compared with their corresponding control mice under HFHC feeding. We made an identical observation in Huh7 cells that either overexpressed or had knockdown of *S100A11* (Supplemental Figure 4, C and D). SREBP2 is also negatively regulated by 25-hydroxycholesterol (25-HC) (34–36). Under normal conditions, we found that addition of 25-HC led to a decrease in intracellular cholesterol content (Supplemental Figure 4E) as well as a reduced accumulation of SREBP2 and HMGCR (Supplemental Figure 4F). This decrease was not observed with overexpression of *S100A11* in Hepa1–6 cells (Supplemental Figure 4, E and F). In addition, under normal conditions, immunofluorescence staining of SREBP2 in Hepa1–6 cells showed that 25-HC enhanced the removal of SREBP2 from the nucleus, while it was retained in the nucleus with overexpression of *S100A11* (Supplemental Figure 4G). Thus, these results suggest that S100A11 activation of SREBP2 may be independent of the classical INSIG/SCAP pathway.

Previous studies identified caspase 2 as a regulator of SREBP2/cholesterol through a nonclassical pathway (37, 38), raising the question of whether S100A11 is involved in the caspase 2/SREBP2 pathway. However, in the liver of mice fed a HFHC diet, either overexpression (Supplemental Figure 4H) or KO (Supplemental Figure 4I) of *S100A11* did not affect the expression of caspase 2. In Huh7 cells, overexpression of *S100A11* did not affect caspase 2 expression (Supplemental Figure 4J). Likewise, overexpression of *caspase2* did not change S100A11 expression (Supplemental Figure 4K). In addition, S100A11 was reported to be involved in immune responses via receptor for advanced glycation end products) (RAGE) or TLR4 (39). However, the expression of RAGE and TLR4 was not changed in the livers of either *S100A11^HepKI^* mice or *S100A11^HepKO^* mice (Supplemental Figure 4, L and M). Taken together, these lines of evidences suggest that the upregulation of SREBP2 by S100A11 may be distinguished from the INSIG/SCAP, caspase 2, and RAGE/TLR4 pathways.

### S100A11 activates SREBP2 cleavage through S1P in the ER.

Next, we asked how S100A11 affects SREBPs nuclear translocation in both Huh7 and Hepa1–6 cell lines. The entry of SREBP2 into the nucleus requires cleavage to release its N-terminal structural domain. SREBP2 is cleaved in 2 steps in the Golgi by site-1 proteases (S1P), site-2 proteases (S2P) (8), or SREBP2 is directly cleaved in the ER via S1P facilitated by caspase 2 (37). Immunofluorescence analysis revealed colocalization of S100A11 with the ER markers calnexin and BIP (Figure 2E and Supplemental Figure 1B), and S100A11 is also regulated by cholesterol (Figure 2, F and G) in the ER, suggesting that S100A11 may sense cholesterol and then directly regulate SREBP2 cleavage in the ER.

S1P is an ER- and Golgi-localized protease that has many mature forms. Previous studies revealed that through autocatalytic processing, S1P produces 2–3 smaller (–30 kDa) intracellular S1P polypeptides (37). Similar to SREBP2 (Figure 4H and Figure 5H), the expression of S1P was increased in the livers of *S100A11^HepKI^* mice (Figure 6A), but was inversely decreased in the livers of *S100A11^HepKO^* mice (Figure 6B). The expression of S2P was unchanged in the livers of both lines of mice (Figure 6, C and D). To test whether S1P or S2P is required for S100A11-triggered SREBP2 cleavage, we generated *S1P*- or *S2P*-knockdown cells using shRNA, in which the reduced expression of these cells was confirmed by Western blotting (Supplemental Figure 5, A and B). Clearly, a reduction of either S1P or S2P could alleviate the increased expression of SREBP2 and HMGCR, as well as the cholesterol accumulation induced by overexpression of *S100A11* (Figure 6, E–G). Treatment of stably overexpressing *S100A11* liver cells with the S1P inhibitor PF429242 (40, 41) also showed similar results (Supplemental Figure 5, C–F). Thus, these results suggest that both S1P and S2P are required for the cleavage of SREBP2 under S100A11 regulation.

Next, co-immunoprecipitation (co-IP) analysis demonstrated that S100A11 was able to physically interact with the S1P –35 kDa sized peptide (Figure 6H), which was increased by overexpression of S100A11 (Figure 6I), similar to the in vivo results (Figure 6A). Likewise, immunofluorescence analysis of Huh7 and Hepa1–6 cells showed colocalization of S100A11 with S1P (Supplemental Figure 5G). In contrast, we found that S100A11 could not interact with or activate S2P (Supplemental Figure 6, H and I). Moreover, deletion of AA23 [S100A11(N)-GFP] to AA86 [S100A11(C-GFP)] in S100A11 failed to interact with S1P and was unable to upregulate SREBP2 and HMGCR or increase cholesterol accumulation (Figure 6, J–L), suggesting that this region is essential and necessary for interaction with S1P to activate SREBP cleavage and function. Taken together, these results indicate that S100A11can both bind to and activate S1P in the ER to cleave SREBP2 for cholesterol metabolism, which is a mechanism similar to that of caspase 2 (37).

### S100A11 promotes SREBP2 nuclear translocation through KPNB.

Although S100A11 interacts with S1P for SREBP cleavage, co-IP analysis did not reveal a direct interaction between S100A11 and SREBP2 (Supplemental Figure 6A), suggesting that S100A11 indirectly regulated the nuclear translocation of SREBP2. It has been shown that SREBP2 can specifically and directly interact with KPNB, a nuclear transporter receptor, to enter the nucleus (42, 43). Application of importazole (IPZ), a KPNB-specific inhibitor (44), could attenuate intracellular cholesterol accumulation (Supplemental Figure 6B) as well as the upregulation of SREBP2 (N) and HMGCR proteins caused by *S100A11* overexpression in Huh7 cells (Supplemental Figure 6C). In addition, immunofluorescence staining showed that transient overexpression of *S100A11* promoted the nuclear accumulation of both SREBP2 and KPNB, and this accumulation was blocked by IPZ (Supplemental Figure 6D). Similar results were confirmed in Hepa1–6 cells (Supplemental Figure 6, E–G). Taken together, these data suggest that S100A11 increases the nuclear accumulation of SREBP2 through the nuclear importer KPNB.

### A S100A11/ANXA1/KPNB axis mediates SREBP2 nuclear translocation.

We next asked whether S100A11 interacted with KPNB to promote SREBP2 nuclear translocation. However, co-IP results did not support a direct interaction between S100A11 and KPNB in 293T cells (Supplemental Figure 7A), implying that other factor(s) may be involved in this process. Therefore, we performed a co-IP and liquid chromatography tandem mass spectrometry (LC-MS/MS) analysis to screen for S100A11-interacting proteins in Hepa1–6 cells (Supplemental Figure 7B). One interesting protein identified was the membrane-associated protein ANXA1, which was detected at high abundance (Supplemental Table 3) and was previously reported to be able to affect KPNB nucleation (45). Similar to SREBP2 (Figure 4H and Figure 5H) and S1P (Figure 6, A and B), the expression of both KPNB and ANXA1 was upregulated in the livers of *S100A11^HepKI^* mice (Figure 7A), but downregulated in the livers of *S100A11^HepKO^* mice (Figure 7B), when compared with their corresponding control, implying that KPNB and ANXA1 may be involved in S100A11 regulation of SREBP activation. Furthermore, as hypothesized, knockdown of *ANXA1* expression could alleviate the upregulated expression of SREBP2(N) and KPNB as well as cholesterol accumulation in both Huh7 (Figure 7, C and D) and Hepa1–6 (Supplemental Figure 7, C and D) cells overexpressing *S100A11*, indicating that ANXA1 mediated S100A11-trigged SREBP2 nuclear accumulation and cholesterol synthesis.

To test the relationships among S100A11, ANXA1, and KPNB, we performed co-IP experiments. These results showed that in 293T cells, S100A11 was able to bind to ANXA1, ANXA1 was able to bind to KPNB, and SREBP2 was able to bind to KPNB (Figure 7, E–H). Furthermore, we constructed exogenous vectors for ANXA1 and KPNB and transfected them into 293T cells. Co-IP assays consistently showed a direct interaction of ANXA1 with KPNB (Supplemental Figure 7, E and F). Immunofluorescence staining also demonstrated colocalization of S100A11 and ANXA1 and of ANXA1 and KPNB in both Huh7 (Figure 7I) and Hepa1–6 cells (Supplemental Figure 7G). More important, Co-IP analysis revealed that the mutual binding of KPNB with either SREBP2 or ANXA1 was enhanced with the overexpression of S100A11 in 293T cells (Figure 7, J and K). Thus, these results uncovered a S100A11/ANXA1/KPNB axis for SREBP2 nuclear import.

Since ANXA1 links S100A11 to KPNB, we examined which region determines its interaction with either S100A11 or KPNB. We generated several truncated ANXA1 proteins, which were fused with GFP and transfected into 293T cells (Figure 7L). IP analysis demonstrated that only the full-length and the C3 segment of ANXA1 had the ability to interact with either S100A11 or KPNB (Figure 7M). In addition, immunofluorescence staining also showed a similar interaction with SREBP2 (Figure 7N). Only the full-length and C3 segments of ANXA1 promoted SREBP2 nuclear accumulation (Figure 7N). Together, these lines of evidence consistently suggested that a S100A11/ANXA1/KPNB axis drives the translocation of SREBP2 into the nucleus.

### In vivo inhibition of SREBP2 signaling alleviates S100A11 promotion of hepatic cholesterol accumulation and MASH.

As shown previously, SREBP2 mediated S100A11 promotion of hepatic cholesterol accumulation and MASH, so we therefore asked whether inhibition of SREBP2 signaling can relieve the above-described symptoms in vivo. Since S1P and ANXA1/KPNB are required for SREBP2 cleavage and nuclear translocation, respectively, we opted to focus on these factors. TAT-NTS, a cell-penetrating peptide derived by combining the transcription activator (Tat) domain with an nuclear translocation signal (NTS) sequence, was reported as an inhibitor of ANXA1-KPNB interaction (45), and PF429242 is an inhibitor of S1P (40, 41). Additional mouse experiments were performed using adeno-associated virus serum type 8–mediated (AAV8-mediated) *S100A11* overexpression (*S100A11*-OE), with or without treatment with either TAT-NTS or PF429242 (Supplemental Figure 8A). These mice and control animals (*GFP*) were subjected to a HFHC diet for 16 weeks.

Compared with the controls (*GFP*), AAV8-mediated *S100A11* overexpression successfully increased liver levels of S100A11 (Figure 8A) and activated liver expression of SREBP2(P), SREBP2(N), HMGCR, the NLRP3 inflammasome (NLRP3, IL1B, and caspase 1–p20), and collagen deposition, as indicated by Masson’s trichrome staining and Picrosirius red staining, and increased cholesterol accumulation, as seen shown by filipin staining (Figure 8, B–E, and G–J). In addition, *S100A11* overexpression increased the ANXA1-KPNB interaction (Figure 8F), plasma levels of ALT, AST, TC, LDL-C, as well as hepatic levels of TC and TG (Supplemental Figure 8, C–I). Taken together, the AAV8-mediated overexpression of *S100A11* definitely phenocopied the *S100A11^HepKI^* mice. More important, all the above phenotypes could be reversed by either TAT-NTS or PF429242 treatment (Figure 8, B–J, and Supplemental Figure 8, C–I). Collectively, these lines of in vivo evidence further strengthen the S100A11/SREBP2 axis in the regulation of hepatic cholesterol metabolism.

## Discussion

Cholesterol synthesis in the liver undergoes strict negative feedback regulation to maintain hepatic cholesterol homeostasis. However, a high-cholesterol diet still promotes hepatic cholesterol accumulation, leading to MASH. In this study, S100A11 was identified as being responsive to and binding of cholesterol. Furthermore, we observed that S100A11 upregulated cholesterol accumulation through a noncanonical S100A11/ANXA1/KPNB/SREBP2 axis, consequently promoting the progression of MASH (Figure 9). S100A11 is a member of the S100 family of calcium-binding proteins and has been reported to be associated with the development of a variety of diseases, including inflammation, autoimmune diseases, hepatic steatosis, and tumorigenesis (46–50). Analysis of integrated transcriptome data from various MASH models under cholesterol induction revealed that S100A11 was positively correlated with cholesterol. Consistently, hepatic *S100A11* expression was positively correlated with TC levels in the liver and serum of patients with MASLD (51). More important, S100A11 can directly bind to cholesterol. In vivo and in vitro results consistently confirmed that overexpression of *S100A11* in liver cells promoted cholesterol synthesis and uptake, accumulation, and MASH; while dysfunction of S100A11 alleviated cholesterol accumulation and MASH, uncovering S100A11 as a regulator of hepatic cholesterol metabolism. Previous studies including ours have showed that S100A11 regulates hepatic steatosis via FOXO1 or AKT/mTOR/SREBP1c signaling (48, 50), indicating that S100A11 may regulate liver TG and cholesterol metabolism via distinct mechanisms, which requires future investigation.

Several key factors, such as SREBP2, LXRA, and NRF1, maintain cholesterol homeostasis in the liver (7, 10–12). LXRA and NRF1 are 2 critical transcription factors that act in response to excessive cholesterol in the liver. They are involved in cholesterol efflux, transport, and excretion to maintain cholesterol homeostasis (10–12). However, dysfunction of either LXRA (10) or NRF1 (11) further increased hepatic cholesterol and exacerbated inflammation in mice when fed a high-cholesterol diet. Unlike LXRA and NRF1, although S100A11 is also a positive responsive factor to excessive cholesterol, knocking out *S100A11* could reduce hypercholesterolemia and hepatic cholesterol accumulation and consequently alleviate inflammation and MASH under high-cholesterol conditions. Thus, targeting of S100A11 may offer an effective therapeutic strategy for treating MASH.

SREBP2 regulates cholesterol homeostasis through a long, well-established negative feedback mechanism, in which low cholesterol levels activate SREBP2 nuclear translocation via INSIG/SCAP to transactivate cholesterol biosynthesis in the liver, whereas high cholesterol levels reversely inhibit this pathway (3, 7–9). However, upregulation of SREBP2 and cholesterol accumulation has been reported in clinical MASH samples and various MASH animal models under cholesterol induction (17, 21). Consistent with these reports, this study found that overexpression of *S100A11* activated SREBP2 nuclear translocation and the expression of its targets HMGCR and LDLR to promote cholesterol biosynthesis, uptake and accumulation in both in vitro liver cell lines and in vivo mouse liver. In contrast, reduction or dysfunction of S100A11 had opposite results. Remarkably, this study revealed that S100A11 activation of SREBP2 was not mediated by the classical INSIG/SCAP pathway. Activation of SREBP2 has also been reported to be mediated via caspase 2, distinct from the classical INSIG/SCAP pathway, under ER stress or a high-fat, high-fructose (HFHFr) diet (37, 38, 52, 53). We determined that S100A11 and the caspase 2/SREBP2–activating pathways are distinct and that S100A11 regulation of SREBP2 and, ultimately cholesterol levels, occurs separately from the previously elucidated pathways.

S100A11 does not directly interact with SREBP2. Co-IP analysis revealed that S100A11 could interact with ANXA1 to activate the SREBP2/cholesterol pathway. S100A11 and ANXA1 have previously been shown to form a connecting channel between the EGFR-containing signaling multivesicular bodies and the ER, facilitating cholesterol transport from the ER to the middle of the multivesicular bodies (54). However, this relationship is unclear. This work revealed that S100A11 could physically interact with ANXA1 and that the C3 segment of ANXA1 was necessary for their interaction as well as SREBP nuclear translocation. Together, these results consistently indicated a role of S100A11 and ANXA1 in regulating cholesterol metabolism.

SREBP2 must be cleaved by S1P/S2P proteases for release of its N-terminal to be imported into the nucleus via importin β (KPNB) in both the negative feedback pathway and the caspase 2 pathway. Consistent with these well-known mechanisms, the cleavage of SREBP2 was also mediated by S1P and S2P, although S100A11 only physically interacts with S1P but not S2P, in the S100A11-activated SREBP2 pathway. ANXA1 was reported to be able to affect KPNB nucleation (45), and KPNB could interact with SREBP2 (43). In support of these results, our co-IP data showed that ANXA1 could directly interact with KPNB, which further interacted with SREBP2, for the entry of SREBP2 into the nucleus. Thus, the downstream signals regarding SREBP2 cleavage and nuclear transportation were highly conserved, regardless of the difference of its upstream activation by negative feedback, caspase 2, or S100A11. More important, although additional in vivo genetic gain- and loss-of-function studies of ANXA1 and KPNB1 as key downstream mediators of S100A11 are needed, we observed that in vitro and in vivo reduction or inhibition of either S1P by PF429242 or ANXA1-KPNB interaction by TAT-NTS could reverse S100A11-promoted SREBP2 activation, thereby alleviating hepatic cholesterol accumulation and MASH, which may provide potential targets for the treatment of cholesterol-related MASH.

## Methods

### Sex as a biological variable.

Sex was not considered as a biological variable in this study; only male mice were used for the MASH model, and the findings may not be directly generalizable to female mice.

### Generation of S100A11-transgenic mice.

The *S100A11* gene–knockin mice (*S100A11^Rosa^*) were generated by Beijing Biocytogen Co., Ltd. under contract. To obtain mice with conditional knockin of *S100A11*, the *Rosa-S100A11* mice on a C57BL/6J background were generated by knocking in *S100A11* at the mouse *Rosa26* locus through a CRISPR/Cas-regulated genome engineering system. Briefly, the *Rosa26-pCAG-loxp-STOP-loxp-mS100A11-CDS-pA* cassette was cloned into intron 1 of *Rosa26*. In addition, homology arms were constructed by PCR for the design of targeting vectors. After this, the targeting vector, gRNA, and Cas9 were coinjected into fertilized eggs to produce *RosaS100A11* mice. A simple schematic diagram is provided in Supplemental Figure 2, A and B.

### Generation of S100A11-KO mice.

*S100A11^fl/fl^* mice were generated by Yingjie Wu (Shandong First Medical University, Jinan City, Shandong Province, China). To generate mice with a conditional KO (cKO) allele of *S100A11*, the *S100A11^fl/fl^* mice on a C57BL/6J background were generated using a CRISPR/Cas9-regulated genome engineering system. Exon 2 and exon 3 of *S100A11* were selected as a cKO region. Briefly, the selected *S100A11* exon was flanked by 2 loxP sites, so 2 sgRNAs (gRNA1 and gRNA2) targeting the *S100A11* intron were designed. The targeting vector containing *S100A11* exons 2–3 with 2 loxP sites and 2 homology arms on the exon 2–3 side was used as a template. The targeting vector, gRNA1, and gRNA2 were injected with Cas9 into fertilized eggs at the single-cell stage. After injection, the surviving zygote was transferred into the oviduct of a pseudopregnant female Kunming mouse. Newborn pups were genotyped by PCR using tail-extracted DNA, and KO alleles were confirmed by PCR amplification sequencing. The founding mice were crossed with WT C57BL/6 mice to produce heterozygous *S100A11^fl/+^* mice, which were then crossed to produce homozygous *S100A11^fl/fl^* mice. A simple schematic diagram is shown in Supplemental Figure 3, A and B.

### Generation of tissue-specific transgenic mice.

To achieve hepatocyte-specific manipulation of *S100A11* expression, the previously generated *S100A11*-overexpressing (*S100A11^Rosa^*) and cKO (*S100A11^fl/fl^*) mice were crossed with albumin-Cre–transgenic mice. Subsequently, the offspring yielding hepatocyte-specific *S100A11-*KI (*S100A11^HepKI^*) and -KO (*S100A11^HepKO^*) models, respectively, were selected for further study. The albumin-Cre mice, which have directed Cre recombinase expression specifically in hepatocytes (55), were gifts from Yonglong Wei (School of Life Sciences, Yunnan University, Kunming, China). Following the breeding strategy, all mice were genotyped by PCR to confirm the presence of the desired alleles. For experimental consistency, only male mice were used in all subsequent analyses.

### A high-fat, high-cholesterol diet–induced mouse model of metabolic dysfunction–associated steatohepatitis.

To establish a mouse model of MASH, 8-week-old male C57BL6J mice were fed a specified HFHC diet (D12108C, Research Diets) for 20 weeks, as previously described, with minor modifications (56). This dietary regimen is widely recognized for its efficacy in inducing key pathological features of human MASH, including hepatic steatosis and fibrosis. Following the 20-week feeding period, all mice were humanely euthanized according to established protocols. Subsequently, liver tissues and other relevant biological samples were systematically collected for subsequent analysis.

### AAV8-mediated S100A11 overexpression.

C57BL/6J mice were purchased from the Animal Center at Yunnan University (Kunming, Yunnan, China). Eight-week-old male C57BL/6J mice were selected. After an acclimatization period, the mice were randomly assigned to groups (at least 10 animals per group). Each mouse received a 100 μL i.v. injection of AAV8-*GFP* or AAV8-*S100A11* adenovirus (purchased from Hanheng Biotechnology) via the tail vein. One week later, the mice were fed a HFHC diet (Research Diet, D12108C). Mice in the HFHC diet group also received weekly i.p. injections of a drug (placebo, TAT-NTS 20 mg/kg or PF429242 20 mg/kg) for 16 weeks; 1 week prior to sample collection, the injection schedule was adjusted to every other day. TAT-NTS was dissolved in saline, while PF429242 working solutions were prepared using a stepwise gradient solvent dissolution method. The solvent system consisted of 10% DMSO, 40% PEG300, 5% Tween 80, and 45% sterile distilled water. Both drugs were purchased from Med Chem Express.

### Husbandry environment.

All animal experiments were performed on male mice, which were housed in a temperature-controlled environment (25°C ± 5°C) under a 12-hour light/12-hour dark cycle. Adequate amounts food and water were provided.

### Cell culture.

HepG2 and Hepa1–6 cells were purchased from the Cell Bank of the Chinese Academy of Sciences. Huh7 and 293T cells were purchased from the Kunming Cell Bank of Type Culture Collection. HepG2, Huh7, Hepa1–6, and 293T cells were cultured in DMEM (Gibco, Thermo Fisher Scientific; C11995500C) supplemented with 10% FBS (Gibco, Thermo Fisher Scientific; 10099–141). The medium was changed every 2 days. Low-cholesterol cell culture environments were cultured in DMEM supplemented with 10% LDS (C3840; VivaCell). For cholesterol treatments, 5 mg/mL cholesterol stock solution was added to the medium to the corresponding treatment concentration. For PA, PS, and CL, the appropriate lipid stock solution was added to the medium to the corresponding treatment concentration.

### Statistics.

Statistical analysis was performed using GraphPad Prism 8 (GraphPad Software) unless otherwise specified. All quantitative data are presented as the mean ± SEM. For comparisons between 2 groups, a 2-tailed, unpaired Student’s *t* test was used. When more than 2 groups were compared, 1-way ANOVA was applied. For comparisons of multiple groups against a single control group, Dunnett’s post hoc test was used. For all pairwise comparisons among groups, a Tukey’s honestly significant difference (HSD) test was applied. *P* values of less than 0.05 were considered statistically significant.

### Study approval.

All animal experiments were conducted according to the guidelines approved by the Animal Ethics Committee of Yunnan University (approval number: 10610).

### Data availability.

The data that support the findings of this study are available from the corresponding authors upon reasonable request. Additional data and raw image files are provided in the Supporting Data Values file.

## Author contributions

BL and MZ conceived and designed the experiments. BL, MZ, and JZ wrote and revised the manuscript. MZ, XX, HW, and JZ performed most of the experiments and data analysis. QF, HL, and CL provided some technical methods. XZ, LZ, TZ, YS, JL, YH, and YW provided the guidance for some experiments and contributed reagents, materials, and analysis tools and revised the manuscript. BL initiated the project. All authors reviewed the manuscript. MZ is listed first because MZ conceived and designed the experiments, wrote and revised the manuscript, and performed most of the experiments. XX is listed second and HW third based on their relative contributions to the experimental work and data analysis, with the order agreed upon by all co–first authors.

## Conflict of interest

The authors have declared that no conflict of interest exists.

## Funding support

- Noncommunicable Chronic Diseases-National Science and Technology Major Project grants 2024ZD0523100 and 2024ZD0523103.
- National Natural Science Foundation of China grants 32371232, 82360726, 32360161, 82300972, and 32360221.
- Yunnan Provincial Science and Technology Project at Southwest United Graduate School grant 202302AO370004.
- The ministry of Science and Technology of the People’s Republic of China grant 2024YFA1802800.
- Yunnan Fundamental Research Projects grants 202301BF070001-023, 202401AS070146, and 202401AT070485.
- Yunnan Xingdian Talent Support Program and the China Postdoctoral Science Foundation grant 2023M731447.

## Supplementary Material

Supplemental data

Unedited blot and gel images

Supporting data values

## Figures and Tables

**Figure 1:**
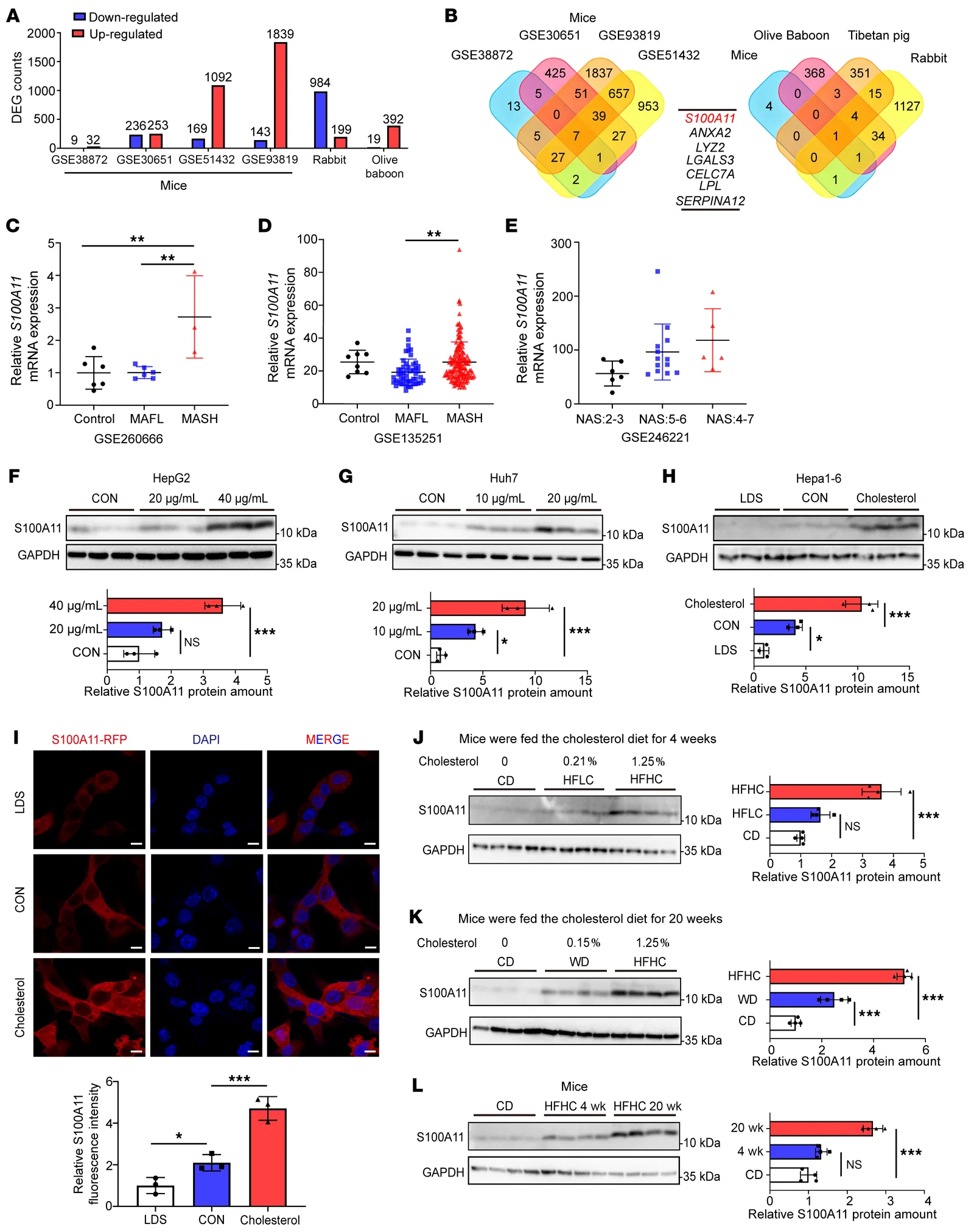
S100A11 positively responds to cholesterol. (**A**) Integrated analysis of DEGs in the liver of various animal models of cholesterol-induced MASH. (**B**) Comprehensive analysis of differentially altered genes in cholesterol diet–induced MASH in multiple species. (**C**–**E**) Expression of liver *S100A11* in human MASLD from Gene Expression Omnibus (GEO) GSE data. NAS, NAFLD activity score. (**F** and **G**) Western blot analysis and quantification of S100A11 protein in HepG2 cells (**F**) and Huh7 cells (**G**) induced by different concentrations of cholesterol (*n* = 3). (**H**) Western blot analysis and quantification of S100A11 protein in Hep1–6 cells induced by either LDS or cholesterol (20 μg/mL) (*n* = 3). (**I**) Fluorescence expression of S100A11-RFP in Hepa1–6 cells induced by either LDS or cholesterol (20 μg/mL) (*n* = 3). Scale bars: 10 μm. Graph shows the quantification. (**J** and **K**) Western blot analysis of S100A11 protein in mouse livers after 4 weeks (**J**) or 20 weeks (**K**) of feeding with cholesterol diets at different concentrations (*n* = 4). Graphs show the quantification. (**L**) Western blot analysis of S100A11 protein in the livers of mice fed a HFHC at different time points (*n* = 4). Graph shows the quantification. GAPDH was used as an internal control for all Western blot analyses. Data are presented as the mean ± SEM. Data were compared using 1-way ANOVA with Tukey’s HSD test (**C** and **D**) or Dunnett’s test (**F**–**L**). *P < 0.05, **P < 0.01, and ***P < 0.001. CON, control; CD, chow diet; WD, Western diet, HFLC, high-fat, low-cholesterol diet; MAFL, metabolic dysfunction-associated fatty liver.

**Figure 2:**
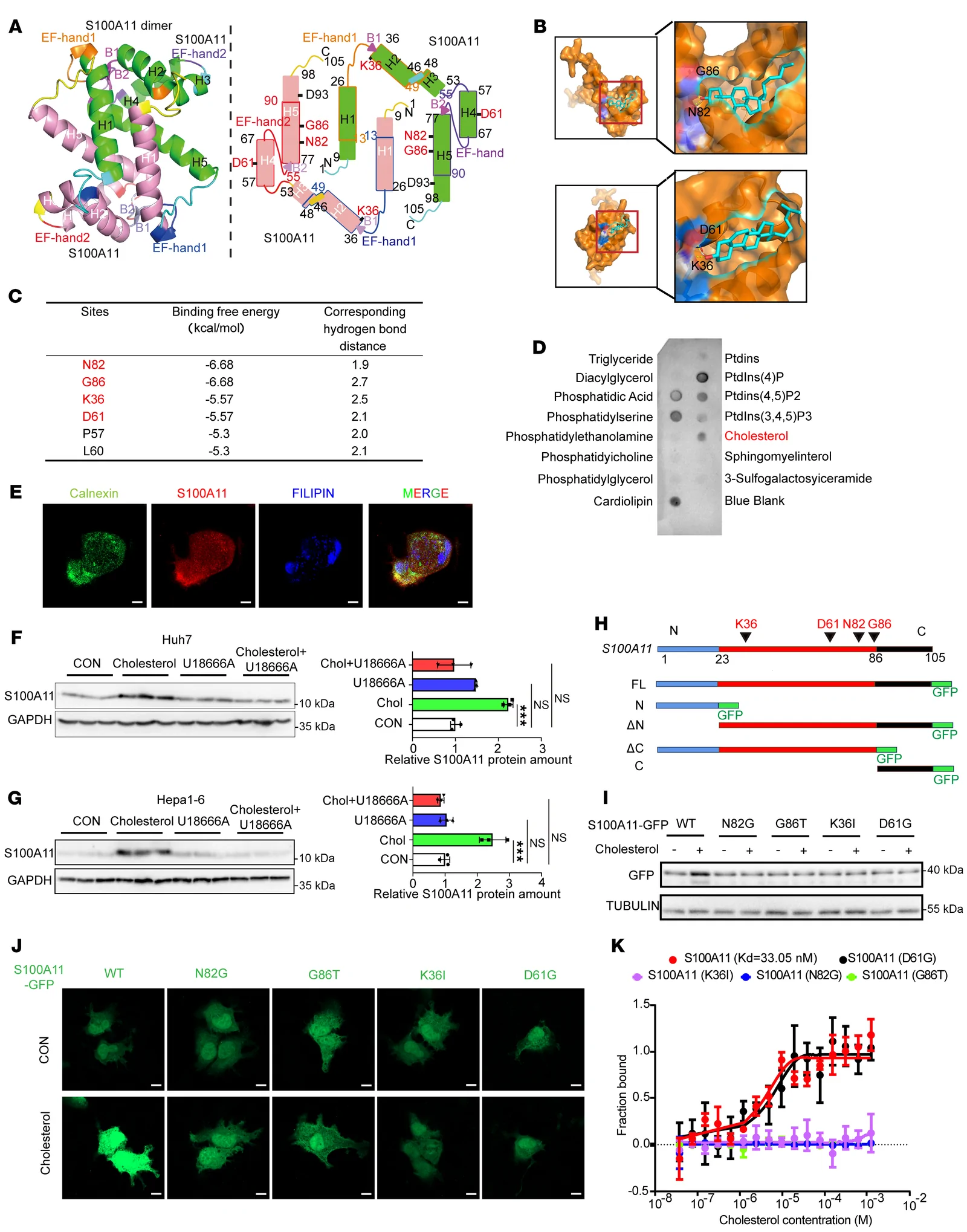
S100A11 can bind to cholesterol. (**A**) The recombinant human S100A11 protein dimer structure (left, Protein Databank [PDB] ID: 2LUC) and its corresponding topological model (right). Light purple and green helices (left) and boxes (right) represent the 5 helical regions of the 2 monomers, respectively. Dark blue and red arrows indicate the 2 β-strands of each monomer. The amino acids shown in red indicate potential cholesterol-binding sites on S100A11. (**B**) Molecular docking results for S100A11 with cholesterol. The predicted pocket positions of cholesterol interaction with S100A11 amino acid residues are shown. (**C**) Results for potential sites of S100A11 binding to cholesterol, the corresponding binding energies, and the hydrogen bonding distances. (**D**) Assay of S100A11 protein binding to the membrane lipid strip. (**E**) Colocalization of S100A11 with the FC stained by filipin and the ER marker calnexin in Huh7 cells. Scale bars: 10 μm. (**F** and **G**) Western blot analysis of S100A11 protein in Huh7 (**F**) and Hepa1–6 (**G**) cells treated with cholesterol (Chol), U18666A, or their combination (*n* = 3). Graphs show the quantification. (**H**) Schematic diagram of GFP-tagged S100A11 truncations. (**I**) Western blot analysis of S100A11 point-mutant protein in Huh7 cells treated with cholesterol. (**J**) Fluorescent expression of GFP-tagged S100A11 point mutations in Huh7 cells treated with cholesterol. Scale bars: 10 μm. (**K**) Cholesterol binding curves for WT and point mutation S100A11 proteins measured by MST. GAPDH or tubulin was used as an internal control for Western blot analysis. Data are presented as the mean ± SEM. Data were compared using 1-way ANOVA with Dunnett’s test. ***P < 0.001.

**Figure 3:**
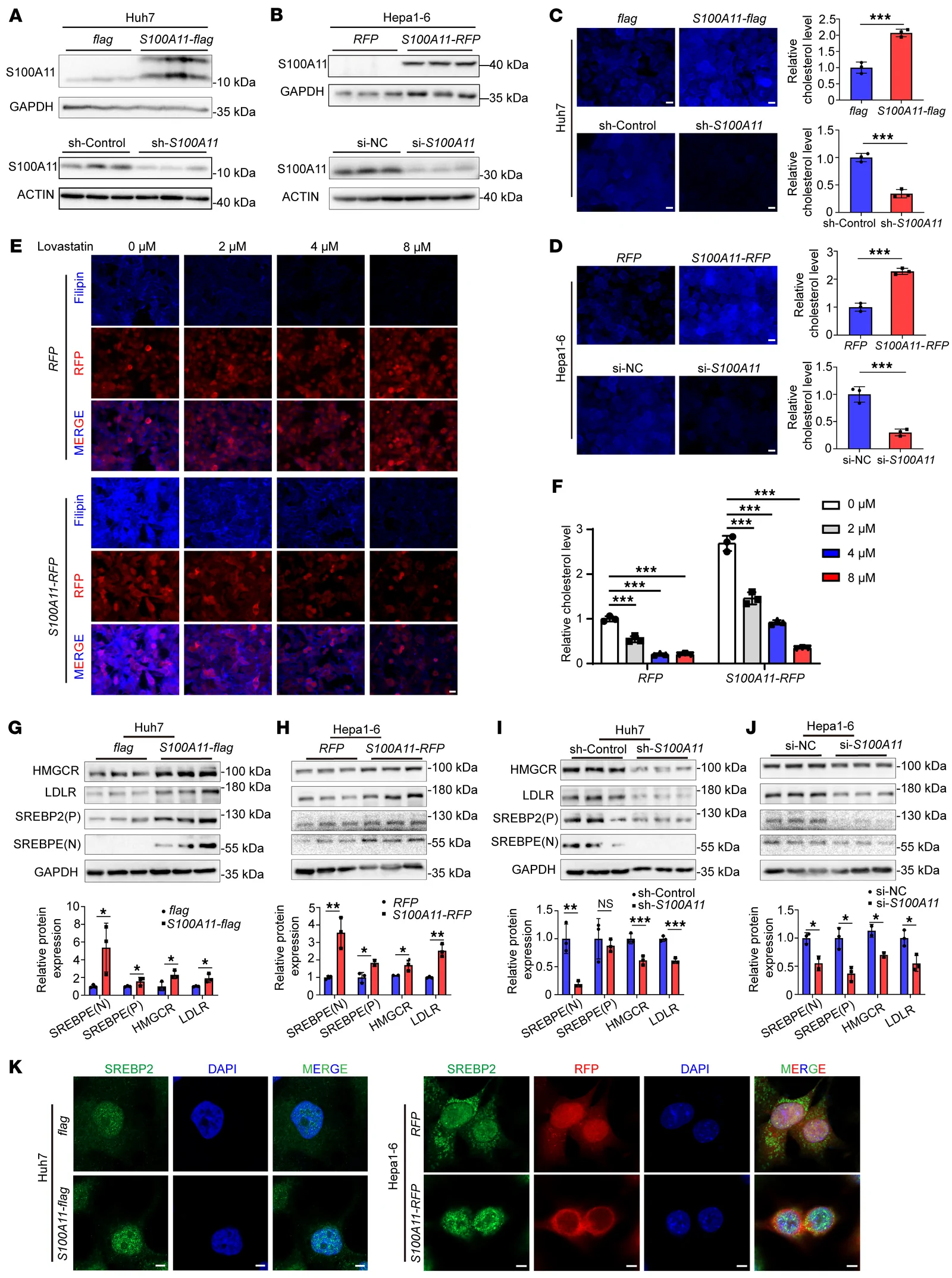
S100A11 is involved in regulating the SREBP2/cholesterol pathway. (**A** and **B**) Western blot analysis of S100A11 protein (overexpression, upper panel; knockdown, lower panel) in Huh7 (**A**) and Hepa1–6 (**B**) cells. (**C** and **D**) Analysis of FC levels by filipin staining in Huh7 (**C**) and Hepa1–6 (**D**) cells (*n* = 3). Scale bars: 20 μm. si-NC, siRNA negative control. (**E** and **F**) Analysis of FC levels by filipin staining in *S100A11*-OE or -knockdown Hepa1–6 cells under lovastatin treatment (**E**) and corresponding quantification (**F**) (*n* = 3). Scale bars: 20 μm. (**G**–**J**) Western blot analysis of the SREBP2 pathway proteins (SREBP, HMGCR, LDLR) in *S100A11*-OE (**G** and **H**) or -knockdown (**I** and **J**) Huh7 and Hepa1–6 cells, respectively. SREBP2(P), precursor form; SREBP2(N), nuclear form. *n* = 3. Graphs show the quantification. (**K**) Immunofluorescence staining to detect SREBP2 in the nucleus in *S100A11*-OE Huh7 and Hepa1–6 cells. Scale bars: 5 μm. GAPDH or actin was used as an internal control for Western blot analysis. Data are presented as the mean ± SEM. Data were compared using a 2-tailed, unpaired Student’s *t* test (**C**, **D**, and **G**–**J**) or 2-way ANOVA with Dunnett’s test (**F**). *P < 0.05, **P < 0.01, and ***P < 0.001.

**Figure 4:**
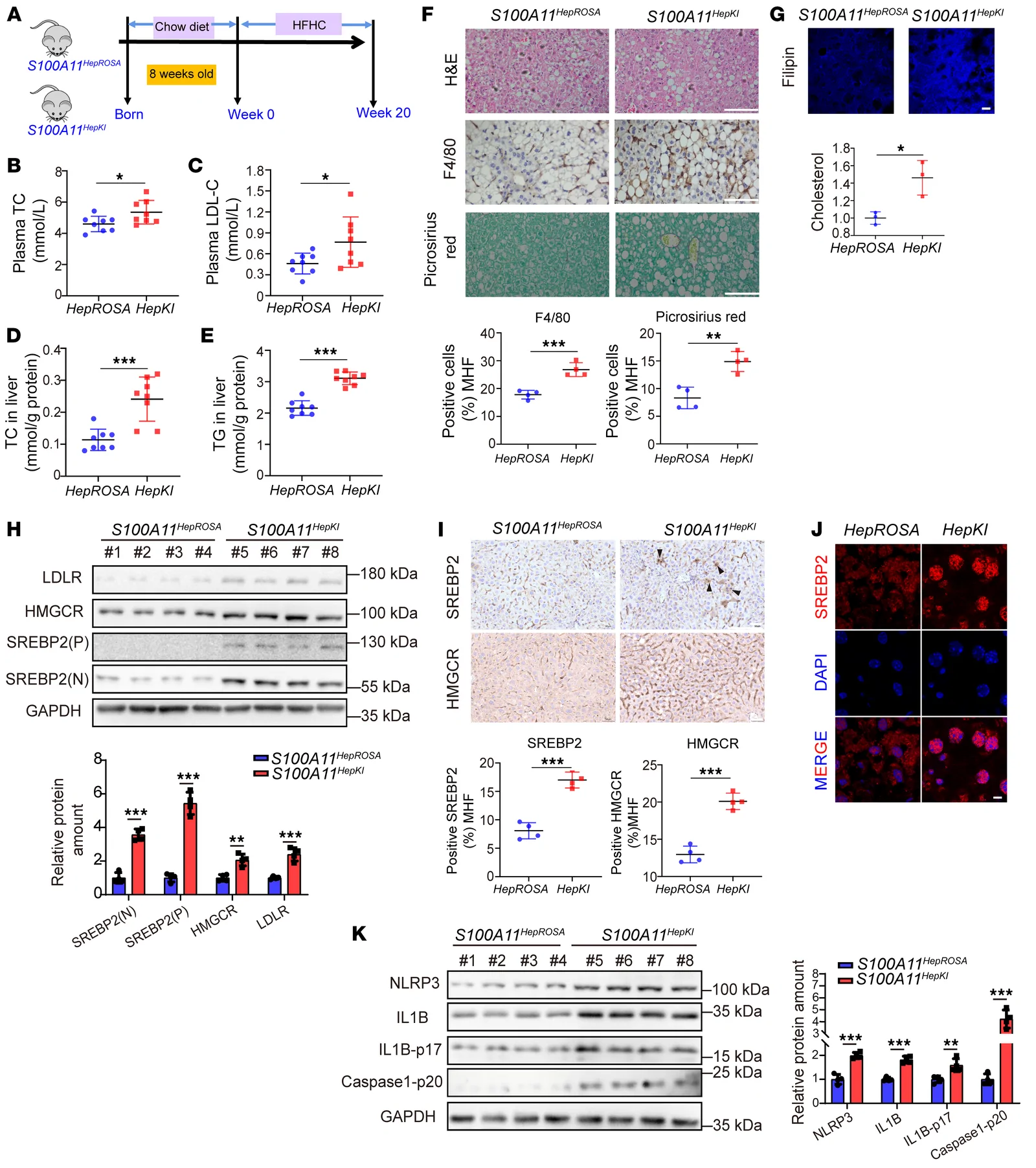
Hepatic overexpression of *S100A11* promotes hepatic cholesterol accumulation and MASH. (**A**) Schematic diagram of the experimental design using a HFHC diet. (**B**–**E**) Plasma levels of TC (**B**) and LDL-C (**C**), as well as liver TC (**D**) and TG (**E**) levels in *S100A11^HepKI^* mice and their littermate controls (*S100A11^HepROSA^*) fed a HFHC diet (*n* = 8–10 per group). (**F**) Liver H&E staining (top), F4/80 staining (middle), and Picrosirius red staining (bottom) in livers of indicated mice fed a HFHC diet (*n* = 4). Scale bars: 100 μm. Quantification was shown below. (**G**) Analysis of FC levels by filipin staining in livers of the indicated mice fed a HFHC diet (*n* = 3). Scale bar: 10 μm. (**H**) Western blot analysis of proteins in the SREBP2 pathway in the livers of the indicated mice fed a HFHC diet (*n* = 4). Quantification was shown below. (**I**) Representative IHC images of SREBP2 and HMGCR in livers of the indicated mice fed a HFHC diet (*n* = 4). White scale bars: 50 μm. Graphs show the quantification. MHF, magnified high-power field. (**J**) Immunofluorescence staining to detect SREBP2 in the nucleus in livers of the indicated mice. Scale bar: 10 μm. (**K**) Western blot analysis of the inflammation pathway in livers of the indicated mice fed a HFHC diet. For each group, data on 4 individual representative mouse samples are shown (*n* = 4). Graph shows the quantification. GAPDH was used as an internal control for all Western blot analyses. Data are presented as the mean ± SEM. Data were compared using a 2-tailed, unpaired Student’s *t* test *, *P* < 0.05, **P < 0.01, and ***P < 0.001.

**Figure 5:**
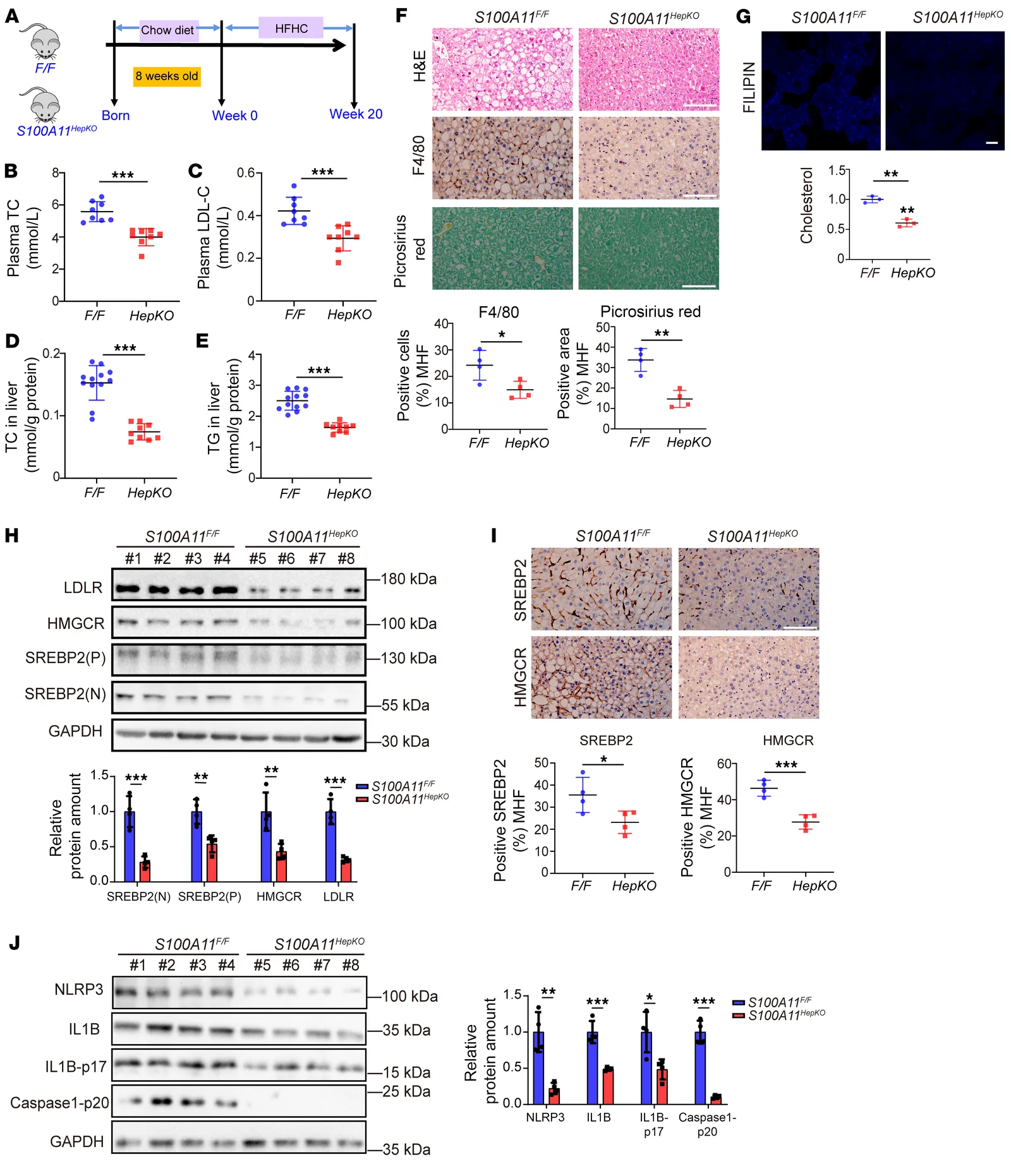
Hepatic KO of *S100A11* alleviates hepatic cholesterol accumulation and MASH. (**A**) Schematic diagram of the experimental design using a HFHC diet. (**B**–**E**) Plasma levels of TC (**B**) and LDL-C (**C**), as well as liver TC (**D**) and TG (**E**) levels in *S100A11^HepKO^* mice and their littermate controls (*S100A11^F/F^)* fed a HFHC diet (*n* = 8–12 per group). (**F**) H&E staining (top), F4/80 staining (middle), and Picrosirius red staining (bottom) in livers of the indicated mice fed a HFHC diet (*n* = 4). Scale bars: 100 μm. Graphs show the quantification. (**G**) Analysis of FC levels by filipin staining in livers of the indicated mice fed a HFHC diet (*n* = 3). Scale bar: 10 μm. Graph shows the quantification. (**H**) Western blot analysis of proteins in the SREBP2 pathway in livers of the indicated mice fed a HFHC diet (*n* = 4). Graph shows the quantification. (**I**) Representative IHC results for SREBP2 and HMGCR in livers of the indicated mice fed a HFHC diet (*n* = 4). Scale bar: 100 μm. Graphs show the quantification. (**J**) Western blot analysis of the inflammation pathway in livers of the indicated mice fed a HFHC diet. For each group, 4 individual representative mouse samples are shown (*n* = 4). Graph shows the quantification. GAPDH was used as an internal control for all Western blot analyses. Data are presented as the mean ± SEM and were compared using a 2-tailed, unpaired Student’s *t* test. *P < 0.05, **P < 0.01, and ***P < 0.001.

**Figure 6:**
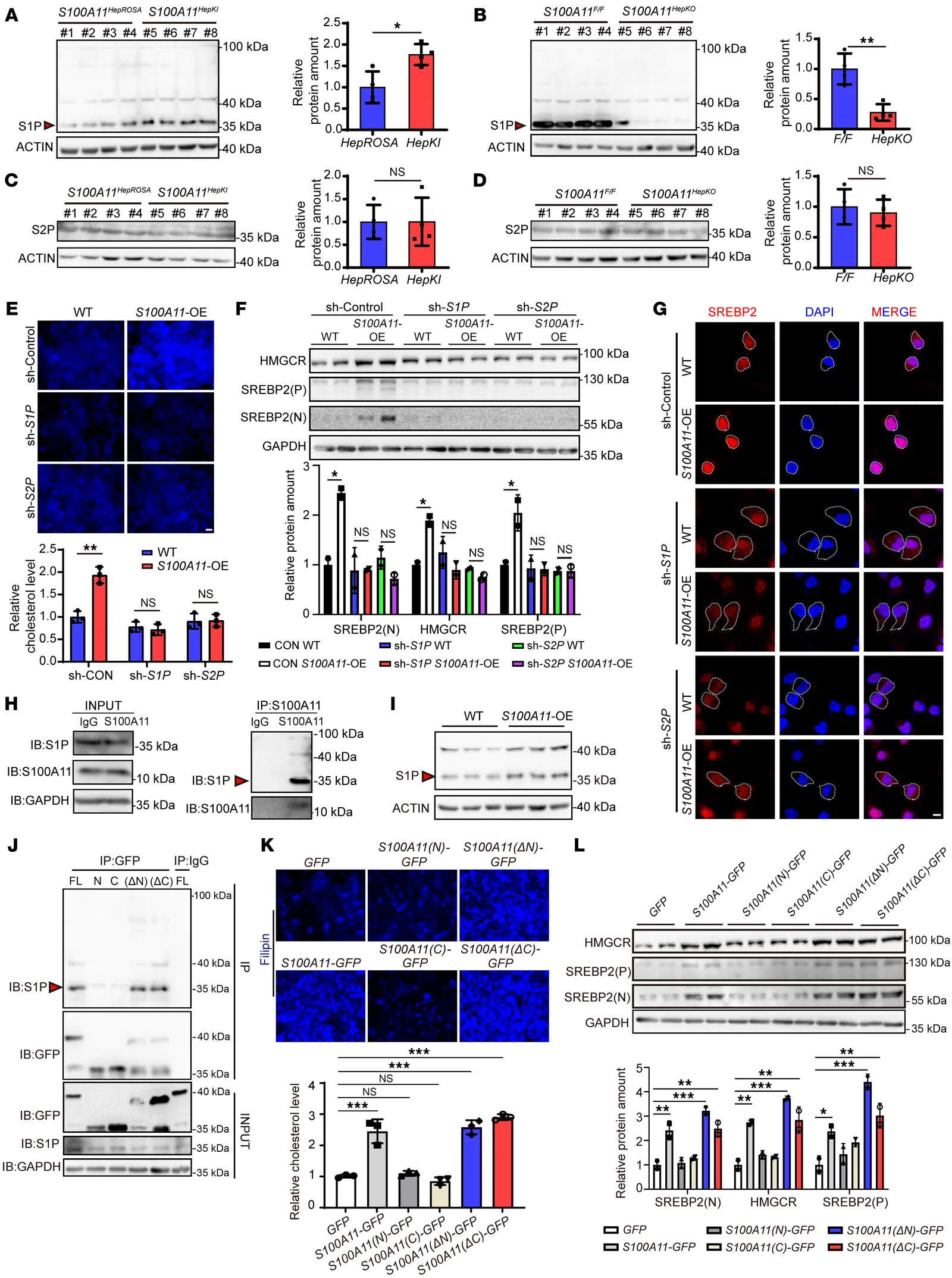
S100A11 directly cleaves SREBP2 via S1P in the ER. (**A**–**D**) Western blot analysis of S1P and S2P proteins in the livers of *S100A11*-OE (*S100A11^HepKI^*) (**A** and **C**) and -KO (*S100A11^HepKO^*) (**B** and **D**) mice (*n* = 4). Graphs show the quantification for the blots. (**E**) Analysis of FC levels by filipin staining in WT or *S100A11-OE* Huh7 cells, following *S1P* or *S2P* knockdown using an shRNA (*n* = 3). Scale bar: 20 μm. Graph shows the quantification. (**F**) Western blot analysis of SREBP2 and HMGCR proteins in WT or *S100A11*-OE Huh7 cells, following *S1P* or *S2P* knockdown using an shRNA. Data reflect 2 representative results from 4 biological replicates (*n* = 4). Graph shows the quantification. (**G**) Cellular localization of SREBP2 in WT or *S100A11*-OE Huh7 cells, following *S1P* or *S2P* knockdown using an shRNA. Scale bar: 10 μm. (**H**) Co-IP analysis of S100A11 and S1P interaction in 293T cells. IB, immunoblot. (**I**) Western blot analysis of S1P proteins in WT or *S100A11*-OE 293T cells. (**J**) Co-IP analysis of S1P and S100A11 truncations in 293T cells. FL, full length. (**K**) Analysis of FC levels by filipin staining in Huh7 cells expressing different *S100A11* truncations (*n* = 3). Original magnification, ×400. Graph shows the quantification. (**L**) Western blot analysis of SREBP2 and HMGCR proteins in Huh7 cells expressing different *S100A11* truncations. Data reflect 2 representative results from 4 biological replicates (*n* = 4). Graph shows the quantification. GAPDH or actin was used as an internal control for the Western blot analyses. Data are presented as the mean ± SEM and were compared using a 2-tailed, unpaired Student’s *t* test (**A**–**D**), 2-way ANOVA with Dunnett’s test (**E**), 1-way ANOVA with Tukey’s HSD test (**F**), or 1-way ANOVA with Dunnett’s test (**K** and **L**). *P < 0.05, **P < 0.01, and ***P < 0.001.

**Figure 7:**
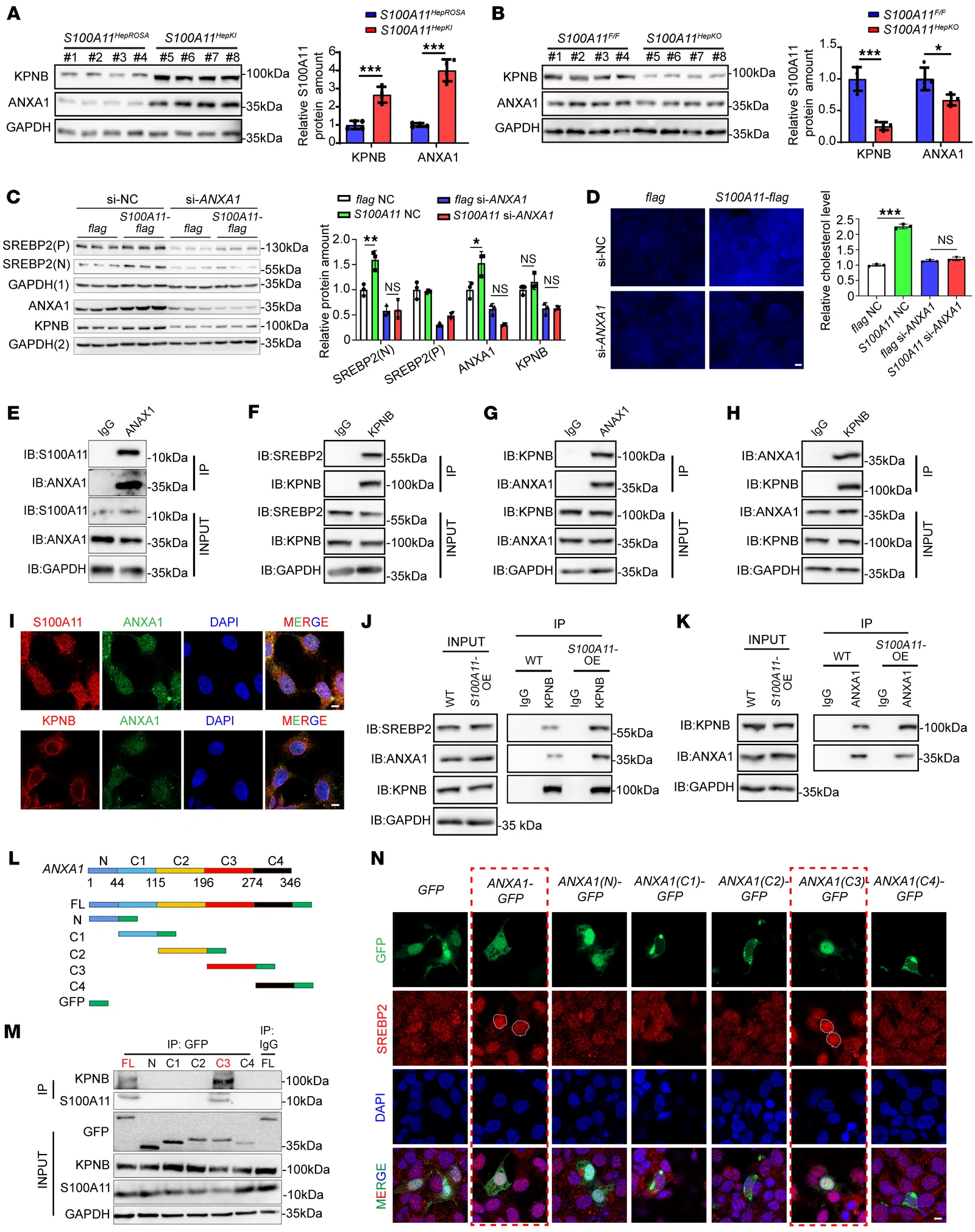
A S100A11/ANXA1/KPNB axis promotes SREBP2 nuclear entry. (**A** and **B**) Western blot analysis of ANXA1 and KPNB proteins in livers of *S100A11-OE* (*S100A11^HepKI^)* (**A**) and -KO (*S100A11^HepKO^*) (**B**) mice (*n* = 4). Graphs in **A** and **B** show the quantification. (**C**) Western blot analysis of SREBP2, ANXA1, and KPNB proteins in 293T cells following *ANXA1* knockdown using an siRNA (*n* = 3). Graph shows the quantification. (**D**) Analysis of FC levels by filipin staining in Huh7 cells following *ANXA1* knockdown using an siRNA (*n* = 3). Scale bar: 20 μm. Graph shows the quantification. (**E** and **F**) Co-IP analysis of the interaction between S100A11 and ANXA1(**E**) and SREBP2 and KPNB (**F**) in 293T cells. (**G** and **H**) Colocalization analysis of S100A11 and ANXA1 (**G**), and KPNB and ANXA1 (**H**) in Huh7 cells. (**I**) Immunofluorescence analysis of the colocalization of ANXA1 with S100A11 or with KPNB. Scale bars: 10 μm. (**J** and **K**) Co-IP analysis of the interaction between KPNB and SREBP (**J**) or between KPNB and ANXA1 (**K**) in WT or *S100A11*-OE 293T cells. (**L**) Schematic diagram of GFP-tagged ANXA1 truncations. (**M**) Co-IP analysis of the interaction of the various ANXA1 truncations with KPNB or S100A11 in 293T cells. (**N**) Immunofluorescence analysis of the colocalization of SREBP2 with different ANXA1 truncations. Scale bar: 10 μm. GAPDH was used as an internal control for all Western blot analyses. Data are presented as the mean ± SEM and were compared using a 2-tailed, unpaired Student’s *t* test (**A** and **B**) or 1-way ANOVA with Tukey’s HSD test (**C** and **D**). *P < 0.05, **P < 0.01, and ***P < 0.001.

**Figure 8:**
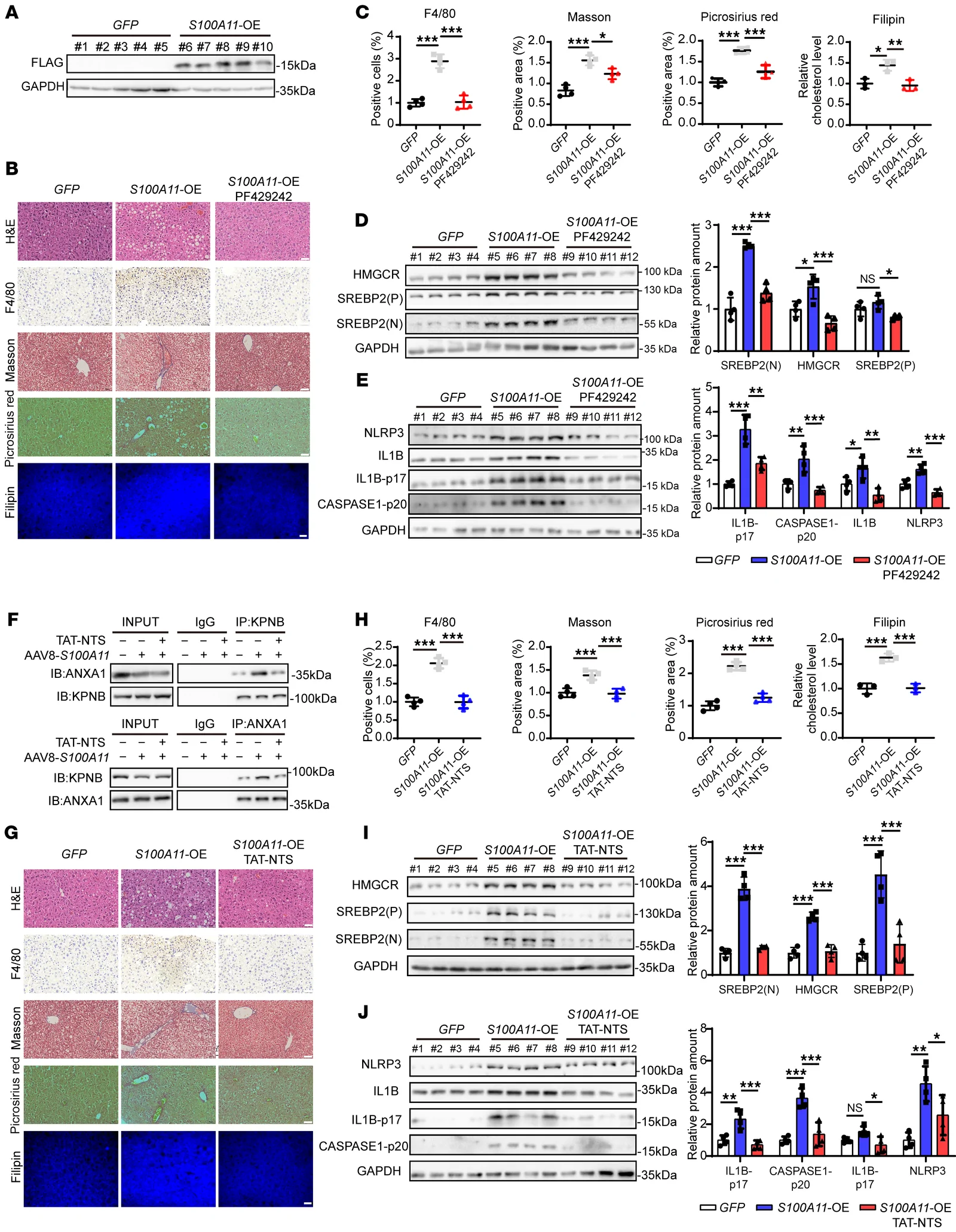
In vivo inhibition of SREBP2 signaling alleviates S100A11-promoted hepatic cholesterol accumulation and MASH. (**A**) Western blot analysis of S100A11 protein in the livers of WT (*GFP*) and *S100A11*-OE mice. (**B** and **G**) H&E, F4/80, Masson’s trichrome, Picrosirius red, and filipin staining in liver tissues from the indicated mice (*n* = 4). Scale bars: 50 μm (H&E, F4/80, Masson’s trichrome) and 10 μm (filipin). (**C** and **H**) Quantification the levels of macrophage (F4/80), collagen fiber (Masson’s trichrome and Picrosirius red) and FC (filipin) from **B** and **G**, respectively. (**D** and **I**) Western blot analysis of SREBP2 and HMGCR proteins in the livers of the indicated mice (*n* = 4). Graphs show the quantification. (**E** and **J**) Western blot analysis of the inflammation pathway in livers of the indicated mice fed a HFHC diet. For each group, 4 individual representative mouse samples (*n* = 4) are shown. Graphs show the quantification. (**F**) Co-IP analysis of ANXA1 and KPNB interaction in the livers of WT (AAV8-*GFP*) and *S100A11*-OE (AAV8-*S100A11*) mice fed a HFHC diet with or without TAT-NTS treatment. For each group, 4 individual representative mouse samples are shown. GAPDH was used as an internal control for all Western blot analyses. Data are presented as the mean ± SEM and were compared using 1-way ANOVA with Dunnett’s test. *P < 0.05, **P < 0.01, and ***P < 0.001.

**Figure 9:**
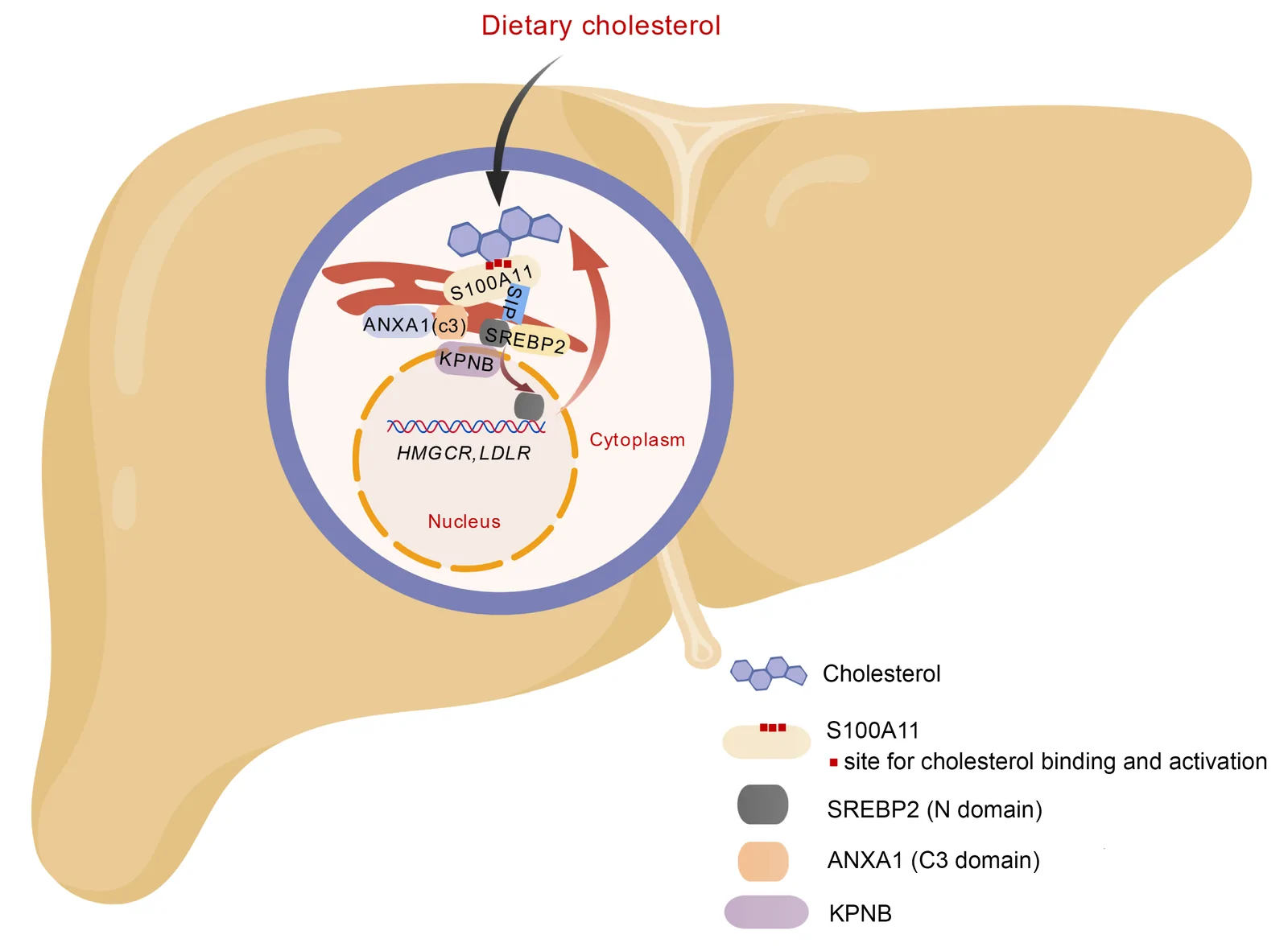
A proposed model of the S100A11/ANXA1/-KPNB/SREBP2 axis regulating liver cholesterol metabolism. Created with BioGDP.com. The binding of cholesterol activates S100A11, which reciprocally interacts with S1P for SREBP2 cleavage and with ANXA1 for KPNB-mediated SREBP2 nuclear translocation, thereby upregulating cholesterol synthesis and uptake and consequently promoting cholesterol accumulation to worsen MASH pathogenesis.

## Version 1 08/04/2026

In-Press Preview

## Version 2 10/01/2026

Electronic publication
