# Supplementary material for: S100A11 regulates hepatic cholesterol metabolism and promotes steatohepatitis via noncanonical SREBP2 signaling
Source: J Clin Invest. 2026 Aug 4;136(19):e201406. doi: 10.1172/JCI201406 (PMC13626821; doi:10.1172/JCI201406)
Supplement: Supplementary file 1 [file jci-136-201406-s021.pdf]

Supplemental Methods

Blood and Liver Samples

After the animals were sacrificed, blood as well as liver samples were collected. The liver samples were quickly frozen in liquid nitrogen and then stored at -80°C until analysis. Blood was centrifuged at 3,000 rpm for 15 min at 4°C to collect serum. An automatic biochemical analyzer from the First People's Hospital of Yunnan Province was used to detect TG (Triglyceride), TC (Total cholesterol), LDL-C (Low-density lipoprotein cholesterol), HDL-C (High-density lipoprotein cholesterol), AST (Aspartate aminotransferase), and ALT (Alanine aminotransferase) in serum.

Lipid measurement and analysis

The contents of TG, TC, and FC in mice liver or cell lines were measured using the kits (Applygen) as below, E1013 for TG, E1015 for TC, and E1016 for FC. All kits were used according to the manufacturer's instructions.

Histological Assays

For paraffin sections, liver samples were fixed in 10% formalin, embedded in paraffin, and sectioned at 4 μm thickness. Sections were stained with HE to assess the gross morphology and the degree of steatosis, and Sirius scarlet stain and Masson's trichrome staining was used to assess the degree of fibrosis. For frozen sections, liver samples were fixed in OCT and snap frozen in liquid nitrogen. After fixation, sections were made at 10 μm thickness using a cryostat (HM525 NX; Thermo Fisher Scientific). The coverslips then were mounted on glass slides with glycerol jelly mounting medium (C0187; Beyotime) and observed under a microscope (BX53; Olympus, Tokyo, Japan) (Axio Imager M2; Carl ZEISS). The degree of cholesterol accumulation was assessed by Filipin staining.

Filipin staining

Specific reagent-treated cells or frozen section samples were fixed with 4%

paraformaldehyde for 30 minutes at room temperature (RT) and then washed 3 times with PBS. Staining was performed with 50 μg/mL working concentration of Filipin solution (SAE00877; Sigma) for 2 hours, the sections were washed 3 times with PBS and finally sealed. All sections were blindly photographed using a microscope in 4 randomized areas and positive areas were quantified using ImageJ software.

Vector construction

The obtained *S100A11*, *ANXA1*, *KPNB*, and *Caspase2* plasmids were cloned into PEGFP-N1 and pCS2+-C-Flag vectors by homologous recombination technique. The obtained truncate of *ANXA1* were cloned into PEGFP-N1 by homologous recombination. The primer sequence information used in this study is shown in **Table S5.**

Cell transfection and inhibitor treatment

For the constructed vectors and siRNA transfection, transfection was performed according to the instructions of Lipo3000 (L3000015; Thermo Fisher Scientific), and samples were collected by removing the medium after 48-72 hours of transfection according to the experimental purpose. For negative feedback treatment of SREBP2, cells were treated with 25-HC (3 μg/mL) (HY113134; MCE) for 24 hours. For lovastatin treatment, cells were treated for 24 h with different concentrations as shown in the figure legend. For KPNB inhibition, the samples were treated with the specific inhibitor Importazole (HY101091; MCE) at a final concentration of 10 μM. For S1P inhibition, inhibition was performed by the specific inhibitor PF429242 (HY13447; MCE). For cholesterol transport inhibition treatment, U18666A (10 μM) (HY107433; MCE) was added to the cells and treated for 24 hours.

Construction of stable transformed cell lines

In this study, both stable knockdown and overexpression of genes in cells were achieved by lentiviral infection. Lentiviral packaging plasmids were psPAX2 (Plasmid #12260) and pMD2.G (Plasmid #12259). The overexpression vector was pCDH (Plasmid

#72265) and the knockdown vector was pLKO.1 (Plasmid #10878) carrying a hairpin structure. The transfection method was calcium transfection. For details, please refer to the following website: https://www.addgene.org/protocols/plko/. Briefly, lentiviral vectors were used to generate *S100A11-FLAG*, *S100A11-FLAG-RFP,* control *FLAG*, control *RFP,* sh-*S100A11*, sh-*S1P*, and sh-*S2P* lentiviral constructs, and the lentiviruses were then packaged in the mature HEK293T cell line. Hepatoma cell lines (Huh7 cells and Hepa1-6 cells) were then infected with lentivirus and screened with puromycin for 2 weeks. After screening, positive monoclonal cells were screened from the enriched cell mixture and verified by western blot **(Figure 3A-B) (Figure S5A-B)**.

Immunofluorescence Assays

The treated cell or tissue samples were fixed with 4% PFA for 15 minutes at RT, washed three times with PBS for 5 minutes each, and then treated with 0.1% Triton X-100 for 15 minutes at RT. Next the samples were incubated with 5% blocking serum for 30 min at RT. After blocking, the corresponding primary antibody was diluted at a specific proportion in primary antibody dilution buffer. The sample slides with the relevant primary antibody were incubated overnight at 4°C. The cells were washed three times with PBS, and then incubated with the relevant secondary antibody for one hour at RT in a dark environment. The cells were washed three times with PBS, incubated for 5 minutes with DAPI (1:1000) at RT, washed three more times with PBS, and finally sealed. Data were collected using fluorescence microscopy or laser confocal microscopy (Axio Imager M2 and LSM800; Carl ZEISS).

Protein extraction and Western Blot assays

Protein extraction and western blotting were performed as previously described (2). Briefly, liver tissues or cultured cells were lysed using ice-cold RIPA buffer supplemented with protease and phosphatase inhibitors. The lysates were centrifuged at 12,000 × g for 15 minutes at 4 °C to collect the supernatant. Subsequently, the protein supernatants were mixed with an equal volume of 2× SDS loading buffer and denatured

by heating at 95 °C for 5 min.

Following denaturation, equal amounts of protein were separated by SDS-polyacrylamide gel electrophoresis and electrophoretically transferred to PVDF membranes. After transfer, the membranes were blocked with 5% non-fat milk in TBST for 1 hour at RT and then incubated with specific primary antibodies diluted in blocking buffer overnight at 4 °C. The next day, membranes were washed and incubated with appropriate conjugated secondary antibodies for 1 hour at RT.

Finally, protein bands were visualized using an enhanced chemiluminescence substrate, and images were captured using Sage Capture system. Band intensities were quantified with LANE 1D software (Sage Creation Science). Detailed information regarding all antibodies used in this study is shown in **Table S4**.

Co-immunoprecipitation (Co-IP) assays

Co-immunoprecipitation assays were performed to investigate protein-protein interactions as previously described (2). Total protein was extracted from 293T cells or mouse liver tissues using RIPA lysis buffer, as described in the previous section. For each reaction, a predetermined amount of protein lysate was incubated with the corresponding primary antibody under gently agitation overnight at 4°C. To control for non-specific binding, parallel samples were incubated with an equivalent amount of species-matched control IgG (mouse or rabbit).

The following day, protein A agarose (ab193255; Abcam) or protein G agarose (20398; Thermo Fisher Scientific) beads were added to the lysate-antibody mixture, selecting the bead type based on the optimal binding affinity for the primary antibody species. The samples were then incubated with continuous mixing for 4 hours at 4 °C. Subsequently, the beads were collected and washed four times with ice-cold IP lysis buffer to remove non-specifically bound proteins. Finally, the immunoprecipitated complexes were eluted by boiling in 2× SDS loading buffer at 95 °C for 5 minutes and subjected to western blot analysis.

Protein-Lipid Binding Assay Using Membrane Lipid Strips

Protein-lipid binding interactions were assessed using pre-spotted membrane lipid strips (P-6002; Echelon Biosciences) according to the manufacturer's protocol and established methodologies (3). Briefly, the membrane was first blocked by incubation with 3% bovine serum albumin (BSA) in TBST for 1 hour at RT to prevent non-specific binding. Subsequently, it was incubated with a solution of recombinant human S100A11 protein (HY-P72482; MCE) for 1 hour at RT to allow for lipid binding.

Following protein incubation, the membrane was washed three times with TBST to remove unbound protein and then probed with a primary antibody against S100A11, diluted in blocking buffer, overnight at 4 °C. The next day, the membrane was washed again and incubated with the secondary antibody for 1 hour at RT. After a final series of washes, the specific binding of S100A11 to various lipids was visualized using Chemiluminescent or enhanced chemiluminescence (ECL) detection system and captured digitally.

Protein-small molecule docking experiment

Molecular docking simulations were performed to investigate the binding affinity and interaction modes between S100A11 and cholesterol, following established computational protocols (4-6). Initially, the three-dimensional chemical structure of cholesterol (CID: 5997) was retrieved in Mol2 format from the PubChem database (https://pubchem.ncbi.nlm.nih.gov/). Subsequently, the structure was energetically minimized using Chem3D software and prepared for docking with AutoDockTools 1.5.7, where partial charges were assigned and torsional roots were defined. For the protein target, the predicted structure of human S100A11 (UniProt ID: P31949) was obtained from the AlphaFold database. Prior to docking, the protein structure was prepared by removing water molecules, adding hydrogen atoms, and assigning charges using AutoDockTools. Finally, both the ligand and receptor were converted to PDBQT

format and subjected to molecular docking analysis using AutoDock, with the grid box dimensions set to encompass the entire protein structure. The resulting docking poses were visualized and analyzed using PyMOL Molecular Graphics System.

Gene Expression Omibus Analysis

To explore mRNA expression profiles associated with diet-induced steatohepatitis, we analyzed publicly available transcriptomic datasets from the Gene Expression Omnibus(GEO) repository *(https://www.ncbi.nlm.nih.gov/geo/)*. Specifically, datasets were selected based on the use of animal models fed a high-cholesterol diet (HC), Western diet (WD), or high-fat, high cholesterol diet (HFHC). Subsequently, gene expression patterns related to steatohepatitis pathogenesis were systematically evaluated across these dietary interventions. A detailed description of the included datasets, along with their respective accession numbers and experimental conditions, is provided in **Table S1**.

Cloning and expression of S100A11 and its point mutants

The protein-coding sequences of wild-type S100A11 and its point mutants (K36I, D61G, N82G, and G86T) were amplified by PCR from cDNA derived from the Huh7 cell line. The amplified fragments were cloned into the pET-28a(+) vector *via* conventional restriction digestion and ligation using NcoI and XhoI restriction sites (7). These constructs enabled the expression of the target proteins fused with a C-terminal 6×His tag. All recombinant plasmids were verified by DNA sequencing.

The validated plasmids were transformed into *Escherichia coli* BL21 (DE3) competent cells. Single colonies were picked and inoculated into Lysogeny Broth (LB) medium supplemented with 50 μg/mL kanamycin, followed by incubation at 37 °C for 12 h. The starter cultures were then inoculated into 1 L of fresh LB medium containing 50 μg/mL kanamycin and grown at 37°C until the optical density at OD_{600} reached 0.6 ~ 0.8. Protein expression was subsequently induced by the addition of 0.5 mM isopropyl β-d-1-thiogalactopyranoside (IPTG), followed by further incubation at 16 °C for 20 h. Cells

were harvested by centrifugation, flash-frozen in liquid nitrogen, and stored at -80 °C until further use.

Protein purification of S100A11 and its point mutants

To purify wild-type S100A11 and its point mutants (K36I, D61G, N82G, and G86T), the frozen cell pellets were resuspended in lysis buffer containing 50 mM Tris-HCl (pH 8.0), 200 mM NaCl, and 1 mM phenylmethylsulfonyl fluoride (PMSF). The cell suspension was disrupted using a high-pressure homogenizer (JNBIO, Guangzhou, China) at 1,000 bar for four cycles. After centrifugation at 20,000 × g for 30 min at 4 °C, the supernatant containing the soluble protein was loaded onto a disposable column packed with 1 mL of Ni-NTA agarose beads (GE Healthcare). After extensive washing steps, the bound proteins were eluted with an elution buffer consisting of 20 mM Tris-HCl (pH 8.0), 150 mM NaCl, and 200 mM imidazole. The eluates were concentrated and further purified using a Superdex 75 Increase size-exclusion chromatography column equilibrated with the aforementioned buffer (7). The concentration of each purified protein was determined, and the samples were aliquoted and stored at -80°C.

Microscale thermophoresis (MST) assay

To evaluate the binding affinity of cholesterol to full-length S100A11 and its point mutants, the proteins were purified in vitro using the *E. coli* prokaryotic expression system as described above (7). Protein labeling was performed using the Monolith His-Tag Labeling Kit RED-tris-NTA 2nd Generation (MO-L018, NanoTemper Technologies). Briefly, 100 μL of each His-tagged protein (wild-type S100A11 or the K36I, D61G, N82G, and G86T mutants at 200 nM) was mixed with 100 μL of the fluorescent dye (100 nM) and incubated for 30 min at RT in the dark. Following centrifugation at 15,000 × g for 10 min at 4 °C, the supernatant was collected for binding assays using the Monolith NT.115 instrument (NanoTemper Technologies). The reaction mixtures containing the labeled proteins and varying concentrations of cholesterol were loaded into Monolith NT.115 capillaries (NanoTemper Technologies,

Cat# MOK022). All experiments were performed in triplicate. Data analysis was carried out using the manufacturer's software (NanoTemper Technologies) to determine the dissociation constant Kd.

Reference

1. Charlton M, Krishnan A, Viker K, et al. Fast food diet mouse: novel small animal model of NASH with ballooning, progressive fibrosis, and high physiological fidelity to the human condition. *Am J Physiol Gastrointest Liver Physiol.* 2011;301(5):G825-834.
2. Zhang L, Zhang Z, Li C, et al. S100A11 promotes liver steatosis via FOXO1-mediated autophagy and lipogenesis. *Cell Mol Gastroenterol Hepatol.* 2021;11(3):697-724.
3. Dowler S, Kular G, and Alessi DR. Protein lipid overlay assay. *Sci STKE.* 2002;2002(129):pl6.
4. Song Z, Han C, Luo G, et al. Yinqin Qingfei granules alleviate Mycoplasma pneumoniae pneumonia via inhibiting NLRP3 inflammasome-mediated macrophage pyroptosis. *Front Pharmacol.* 2024;15:1437475.
5. Zou J, Xu W, Li Z, et al. Network pharmacology-based approach to research the effect and mechanism of Si-Miao-Yong-An decoction against thromboangiitis obliterans. *Ann Med.* 2023;55(1):2218105.
6. Sun Y, Zhao H, and Wang Z. Compound identification of Shuangxinfang and its potential mechanisms in the treatment of myocardial infarction with depression: insights from LC-MS/MS and bioinformatic prediction. *Front Pharmacol.* 2025;16:1499418.
7. Xiao H, Li S, Qi R, et al. Molecular mechanism of phosphate import by the bacterial PstSCAB transporter. *Nat Commun.* 2026;17(1).

Supplemental Figures

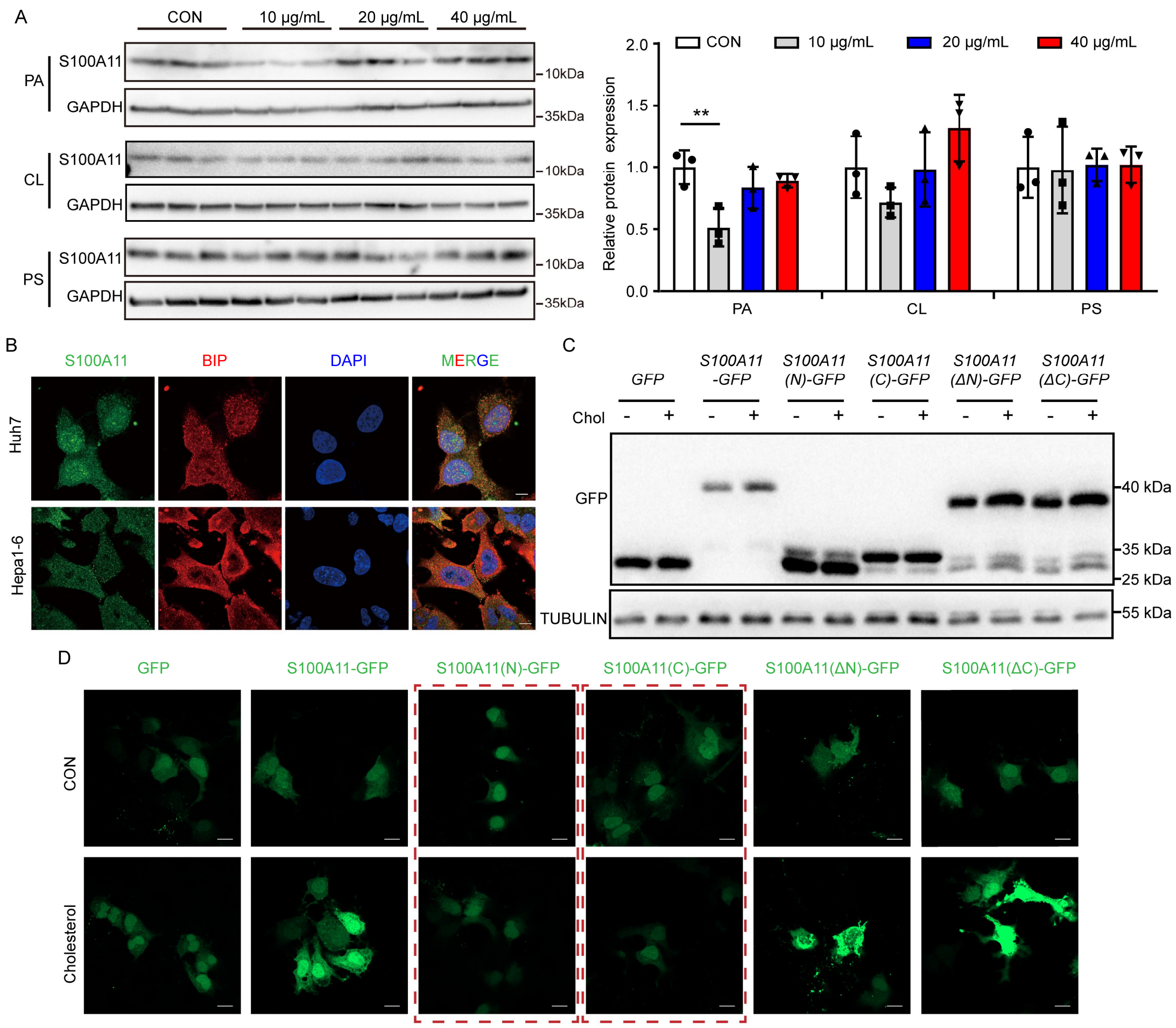

Figure S1. The cholesterol-binding region of S100A11

(A) Western blot analysis of S100A11 protein in Huh7 cells treated with PA, CL, and PS at different concentrations (n=3). Quantification was shown on the right.

(B) Co-localization of S100A11 and the ER marker BIP in Huh7 (top) and Hepa1-6 (bottom) cells. Scale bars: 10 μm.

(C) Western blot analysis of S100A11 truncation proteins in Huh7 cells treated with cholesterol (Chol).

(D) Fluorescent imaging of *GFP*-tagged *S100A11* truncations in Huh7 cells treated with cholesterol. Scale bars: 10 μm.

GAPDH or TUBULIN were used as internal controls for western blot analysis.

Data are presented as mean ± SEM. Data were compared using one-way ANOVA with Dunnett's test. *, $P < 0.05$; **, $P < 0.01$; ***, $P < 0.001$.

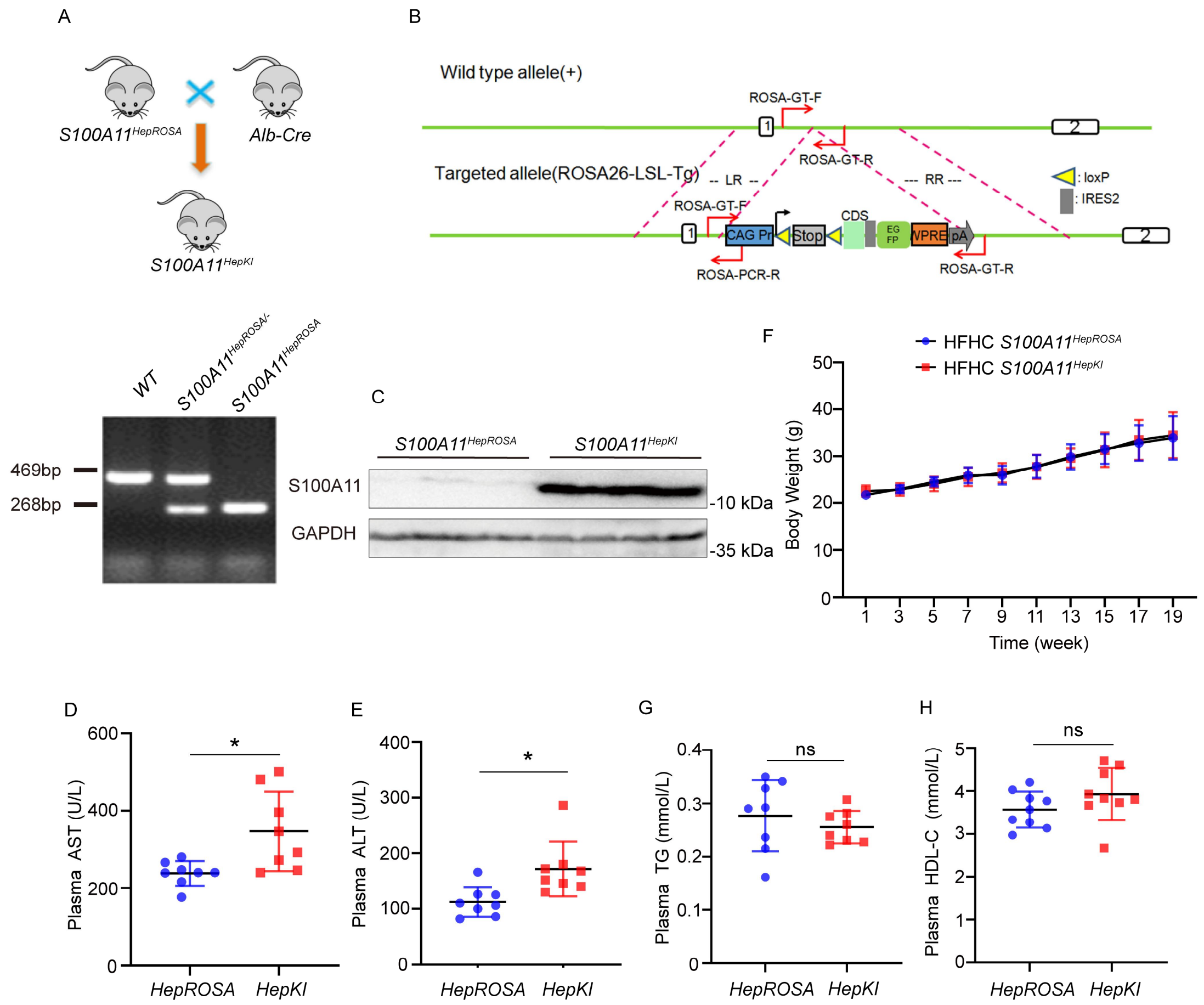

Figure S2. Generation and phenotypes of *S100A11* liver specific overexpressing mice

(A) Generation of hepatocyte-specific knock in mice (top) and PCR (polymerase chain reaction) analysis to confirm the genotypes of wild type (WT), *S100A11$^{HepROSA/-}$* and *S100A11^{HepROSA}* mice (bottom).

(B) Schematic diagram of wild type and knock in allele mice.

(C) Western blot analysis of S100A11 protein in the liver of *S100A11^{HepROSA}* and *S100A11^{HepKI}* mice. Data are presented of four individual animals (n=4) for each mice group.

(D-E) Plasma levels of AST (D) and ALT (E) of *S100A11^{ROSA}* and *S100A11^{HepKI}* mice fed on a HFHC diet (n≥8).

(F) Body weight of *S100A11^{ROSA}* and *S100A11^{HepKI}* mice fed on a HFHC diet. (n≥8).

(G-H) Plasma levels of TG (G) and HDL-c (H) of *S100A11^{ROSA}* and *S100A11^{HepKI}* mice

fed on a HFHC diet (n⩾8).

GAPDH was used as internal control for western blot analysis. Data are presented as mean ± SEM. Data were compared using Student's *t* test. *, $P < 0.05$.

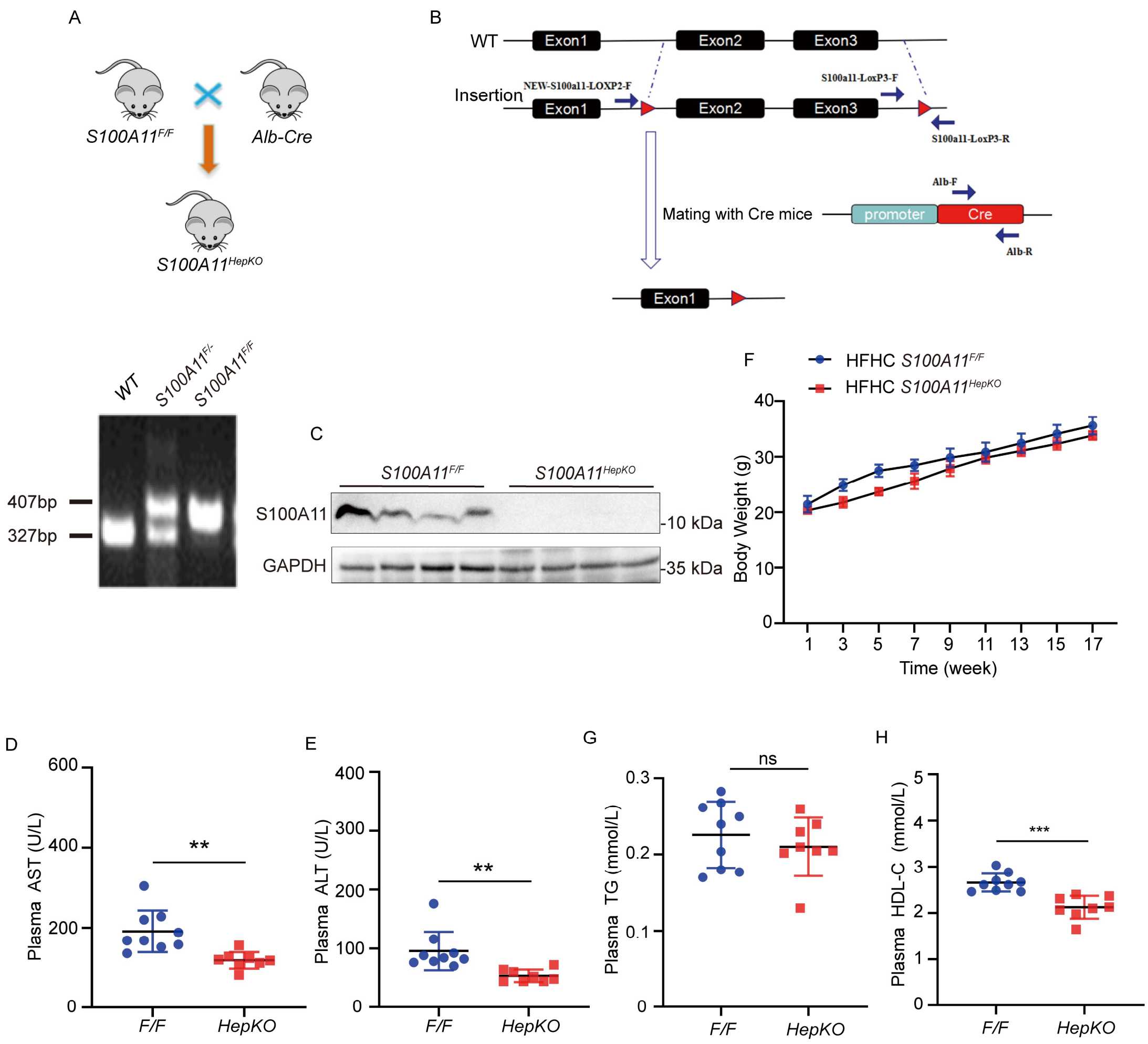

Figure S3. Generation and phenotypes of *S100A11* liver specific knockout mice

(A) Generation of hepatocyte-specific knockout mice (top) and PCR (polymerase chain reaction) analysis to confirm the genotypes of wild type (WT), *S100A11*$^{F/-}$ and *S100A11*$^{F/F}$ mice (bottom).

(B) Schematic diagram of wild type and mutant allele mice.

(C) Western blot analysis of S100A11 protein in the liver of *S100A11*$^{F/F}$ and *S100A11*^{HepKO} mice. Data are presented of four individual animals (n=4) for each mice group.

(D-E) Plasma levels of AST (D) and ALT (E) of *S100A11*$^{F/F}$ and *S100A11*^{HepKO} mice fed on a HFHC diet (n⩾8).

(F) Body weight of *S100A11*$^{F/F}$ and *S100A11*^{HepKO} mice fed on HFHC. (n⩾8).

(G-H) Plasma levels of TG (G) and HDL-c (H) of *S100A11*$^{F/F}$ and *S100A11*^{HepKO} mice fed on a HFHC diet (n⩾8).

GAPDH was used as internal control for western blot analysis. Data are presented as mean ± SEM. Data were compared using Student's *t* test. **, $P < 0.01$; ***, $P < 0.001$.

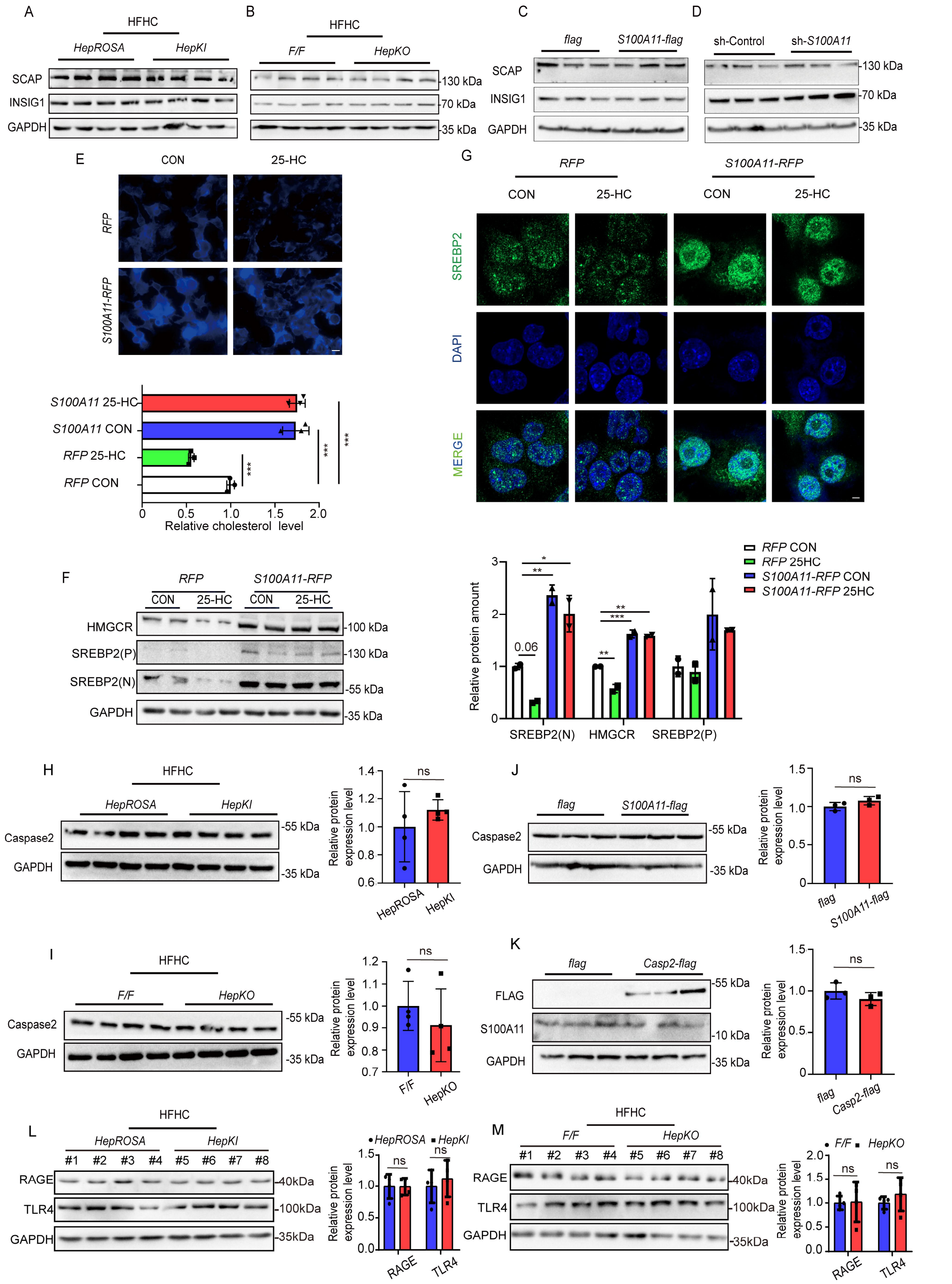

Figure S4. S100A11 mediating SREBP2 nuclear entry is independent of the SCAP-INSIG and Caspase2 pathway

(A-D) Western blot analysis of INSIG and SCAP proteins in the livers of *S100A11*^{HepKI} mice (A) and *S100A11*^{HepKO} mice (B) fed on a HFHC diet, along with *S100A11* overexpressing (C) and knockdown (D) Huh7 cells.

(E) Analysis of free cholesterol levels by Filipin staining in wild type (*RFP*) or *S100A11* overexpression (*S100A11-RFP*) Hepa1-6 cells treated with 25-hydroxycholesterol (25-HC) (n=3) . Scale bars: 20 μm. Quantification was shown below.

(F) Western blot analysis of SREBP2 and HMGCR proteins in wild type (*RFP*) or *S100A11* overexpression (*S100A11-RFP*) Hepa1-6 cells treated with 25-HC. Quantification was shown on the right.

(G) Immunofluorescence analysis of SREBP2 nuclear translocation in wild type (*RFP*) or *S100A11* overexpression (*S100A11-RFP*) Hepa1-6 cells treated with 25-HC. Scale bars: 5 μm.

(H-J) Western blot analysis of Caspase2 protein in the livers of $S100A11^{HepKI}$ mice (H) and $S100A11^{HepKO}$ mice (I) fed on a HFHC diet (n=4), along with *S100A11* overexpressing (J) Huh7 cells (n=3). Quantification was shown on the right.

(K) Western blot analysis of S100A11 protein in the *Caspase2* overexpressing (*Casp2-flag*) Huh7 cells. Quantification was shown on the right.

(L-M) Western blot analysis of RAGE and TLR4 proteins in the livers of $S100A11^{HepKI}$ mice (L) and $S100A11^{HepKO}$ mice (M) fed on HFHC (n=4). Quantification was shown on the right.

GAPDH was used as an internal control for all western blot analyses. Data are presented as mean ± SEM. Data were compared using one-way ANOVA with Dunnett's test(E-F) or Student's *t* test (H-M). *, $P < 0.05$; **, $P < 0.01$; ***, $P < 0.001$.

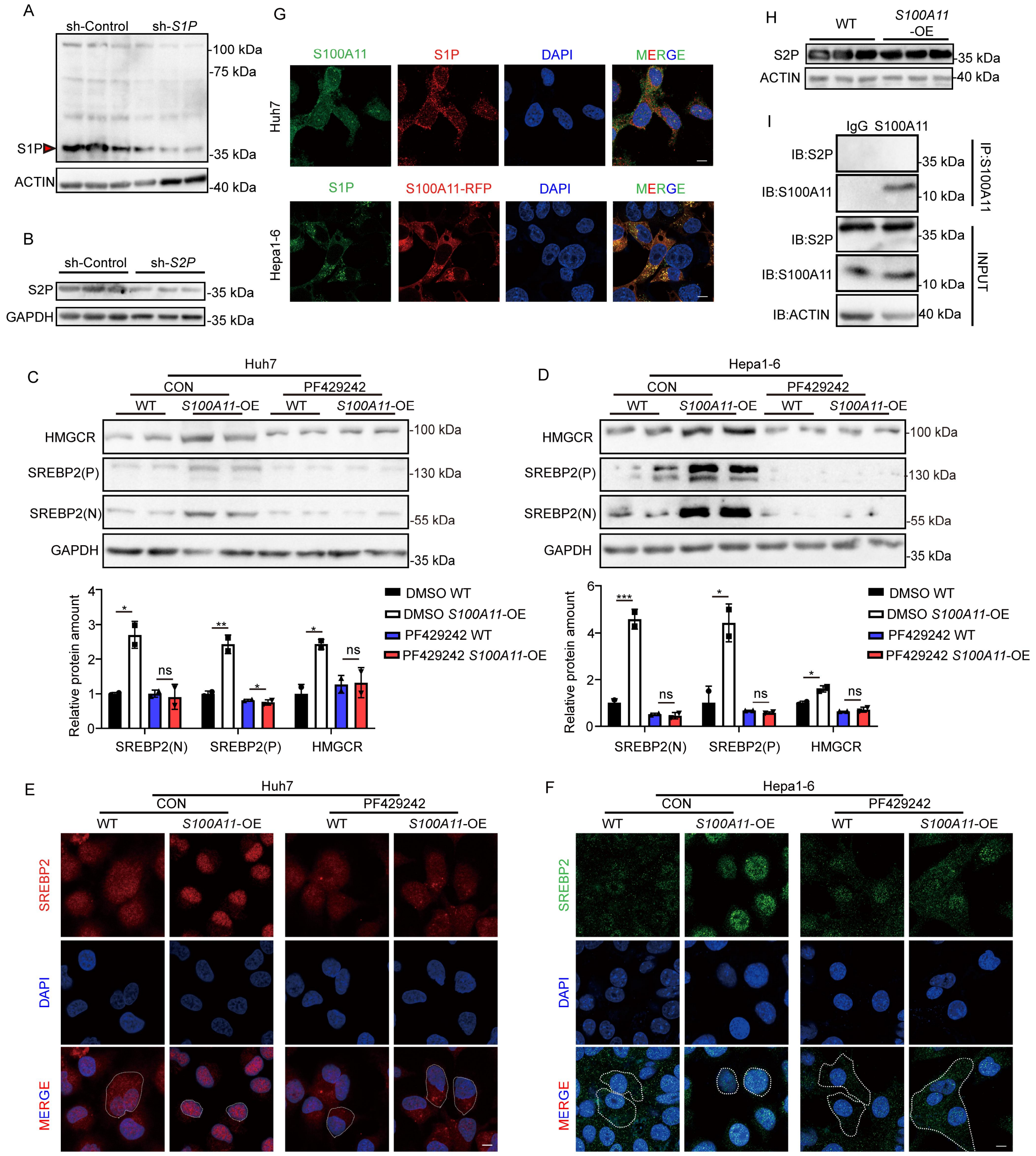

A
sh-Control
sh-S1P
S1P
ACTIN
100 kDa
75 kDa
35 kDa
40 kDa
B
sh-Control
sh-S2P
S2P
GAPDH
35 kDa
G
S100A11
S1P
DAPI
MERGE
Huh7
S100A11-RFP
Hepa1-6
H
WT
S100A11 -OE
S2P
ACTIN
I
IgG
IB:S2P
IB:S100A11
IB:ACTIN
IP:S100A11
INPUT
10 kDa
C
Huh7
CON
PF429242
S100A11-OE
HMGCR
SREBP2(P)
SREBP2(N)
130 kDa
55 kDa
Relative protein amount
DMSO WT
DMSO S100A11-OE
PF429242 WT
PF429242 S100A11-OE
ns
D
Hepa1-6
E
SREBP2
F

(G) Co-localization of S100A11 and S1P in Huh7 (top) and Hepa1-6 (bottom) cells. Scale bar: 10 μm.

(H) Western blot analysis of S2P protein in wild type (WT) or *S100A11* overexpression (*S100A11*-OE) Huh7 cells.

(I) Co-immunoprecipitation (Co-IP) analysis of S100A11 and S2P interaction in 293T cells.

GAPDH or ACTIN was used as an internal control for the western blot analysis. Data are presented as mean ± SEM. Data were compared using one-way ANOVA with Tukey's HSD test. *, $P < 0.05$; **, $P < 0.01$; ***, $P < 0.001$.

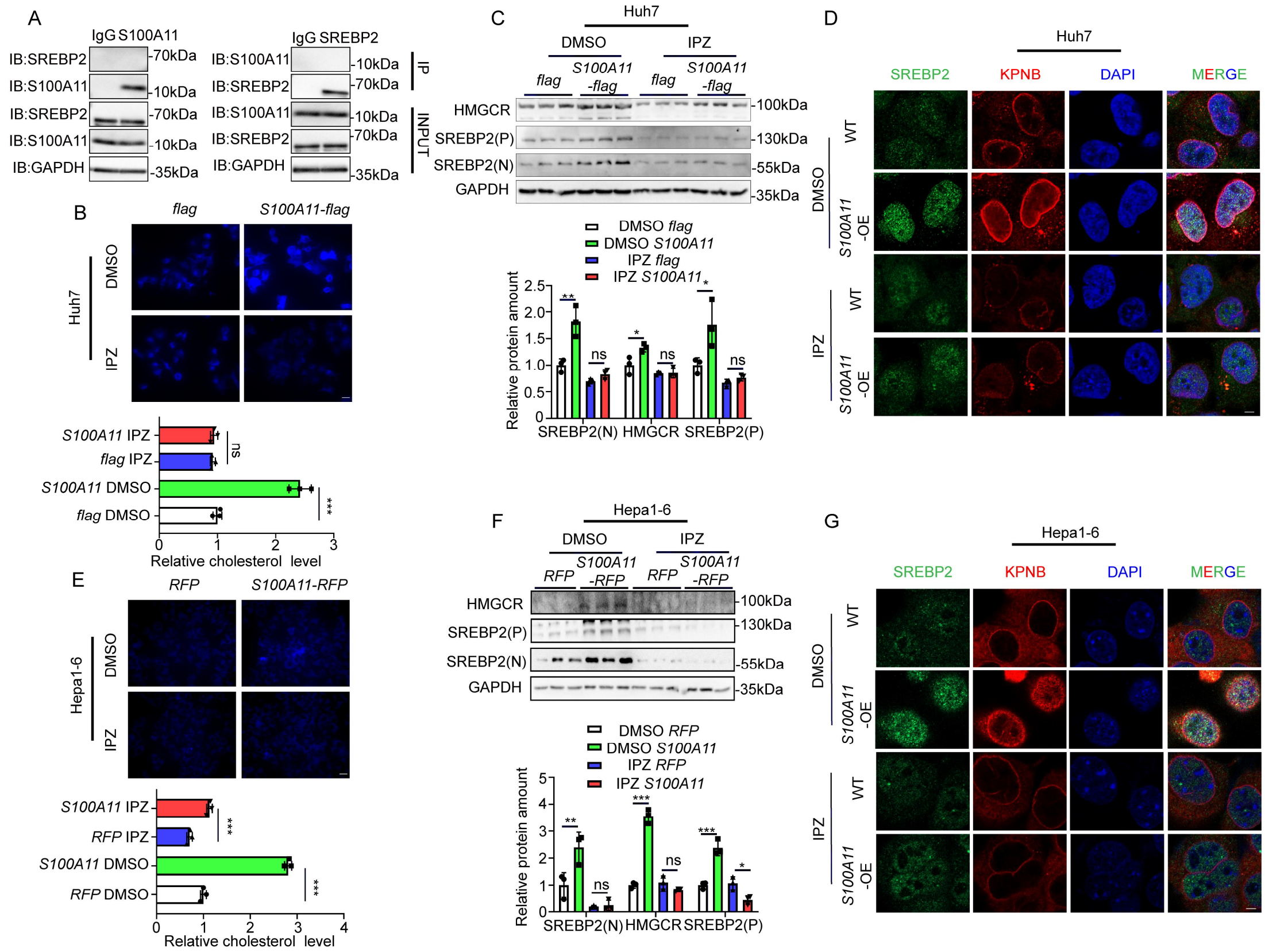

Figure S6. S100A11 promotes SREBP2 nuclear translocation via KPNB

(A) Co-IP analysis of the interaction between S100A11 and SREBP2 in 293T cells.

(B and E) Analysis of free cholesterol levels by Filipin staining in Huh7 (B) and Hepa1-6 (E) cells following IPZ treatment (n=3). Scale bars: 20 μm. Quantification was shown below.

(C and F) Western blot analysis of SREBP2 and HMGCR proteins in Huh7 (C) and Hepa1-6 (F) cells following IPZ treatment (n=3). Quantification was shown below.

(D and G) Immunofluorescence analysis of the co-localization of SREBP2 and KPNB in Huh7 (D) and Hepa1-6 (G) cells following IPZ treatment. Scale bars: 5 μm.

GAPDH was used as an internal control for all western blot analyses. Data are presented as mean ± SEM. Data were compared using one-way ANOVA with Tukey's HSD test. *, $P < 0.05$; **, $P < 0.01$; ***, $P < 0.001$.

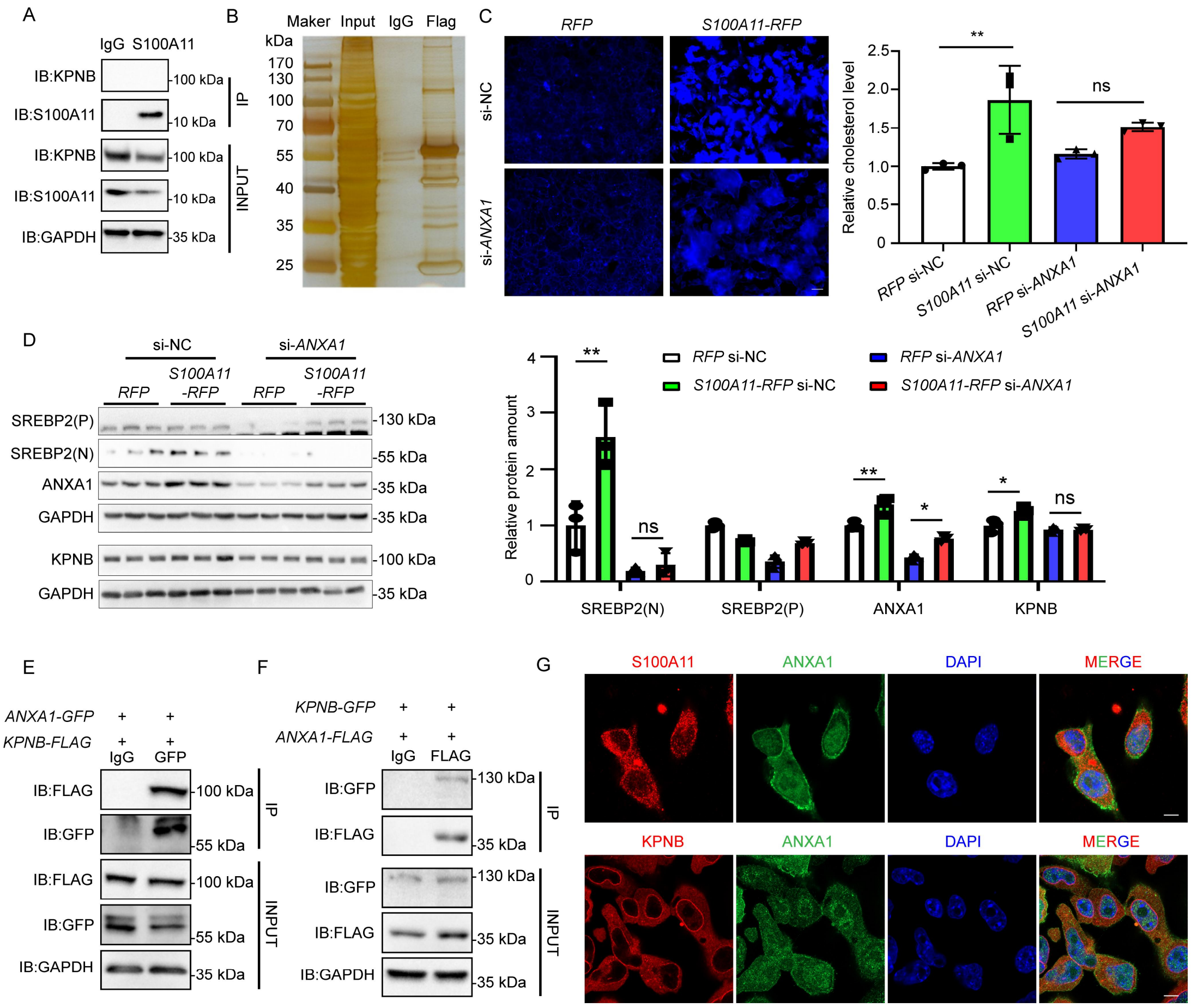

Figure S7. A S100A11-ANXA1-KPNB axis promotes SREBP2 nuclear entry

(A) Co-IP analysis of S100A11 and KPNB interaction in 293T cells.

(B) A silver-stained gel of S100A11 interacting proteins by Co-IP analysis.

(C) Analysis of free cholesterol levels by Filipin staining in Hepa1-6 cells following knockdown by siRNA (n=3). Scale bars: 20 μm. Quantification was shown right.

(D) Western blot analysis of SREBP2, ANXA1, and KPNB proteins in Hepa1-6 cells following knockdown by siRNA (n=3). Quantification was shown right.

(E-F) Co-IP analysis of the interaction between ANXA1 and KPNB (E), and KPNB and ANXA1 (F) in 293T cells. The indicated recombinant plasmids were co-expressed in 293T cells for 48 hr, and the cell lysates were analyzed by Co-IP assay.

(G) Cellular co-localization analysis of ANXA1 with S100A11 (top) or KPNB (bottom) in Hepa1-6 cells. Scale bars: 10 μm.

GAPDH was used as an internal control for all western blot analyses. Data are presented

as mean ± SEM. Data were compared using one-way ANOVA with Tukey's HSD test. *, $P < 0.05$; **, $P < 0.01$; ***, $P < 0.001$.

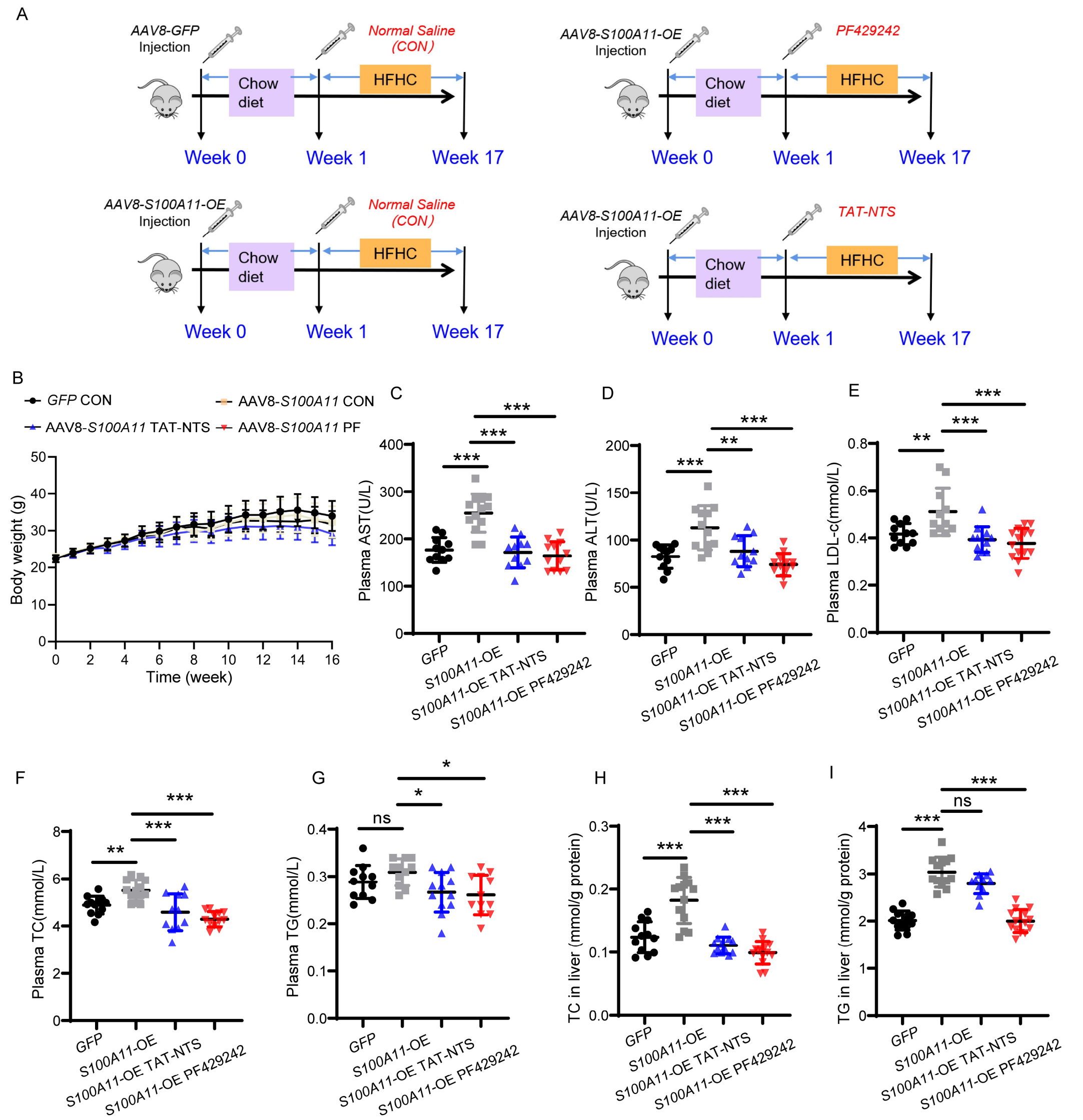

Figure S8. In vivo inhibition of SREBP2 signaling alleviates S100A11 promoted plasma and hepatic symptoms

(A) Schematic diagram of the AAV8-*S100A11* mice experiment following a HFHC diet - treated with or without TAT-NTS or PF429242.

(B) Body weight in the indicated mice fed on a HFHC diet with or without PF429242 and TAT-NTS treatment (n⩾10).

(C-D) Plasma levels of AST (C) and ALT (D) in the indicated mice fed on a HFHC diet with or without PF429242 and TAT-NTS treatment (n≥10).

(E-G) Plasma levels of LDL-c (E), TC(F), and TG(G) in the indicated mice fed on a HFHC diet with or without PF429242 and TAT-NTS treatment (n≥10).

(H-I) The liver TC (H) and TG (I) levels in the indicated mice fed on a HFHC diet with or without PF429242 and TAT-NTS treatment (n≥10).

Data are presented as mean ± SEM. Data were compared using one-way ANOVA with Dunnett's test. *, $P < 0.05$; **, $P < 0.01$; ***, $P < 0.001$.

Supplemental tables

Table S1

Sources and screening criteria for each database

Source	Organism	Treat	log2(Fold_ change)	p-value
GSE38872	Mice	WD	≥1 ； ≤-1	
GSE30651	Mice	HCD	≥1 ； ≤-1	
GSE51432	Mice	HFHC	≥1 ； ≤-1	
GSE93819	Mice	HFHC	≥1 ； ≤-1	
GSE142346	Tibetan pig	HFHC	≥1 ； ≤-1	≤0.05
GSE227346	Papio anubis	HFHC	≥0.8 ； ≤-0.8	
PMID-33075563	Tree threw	HFHC	≥1 ； ≤-1	
PMID-29692426	Rabbit	HCD	≥1 ； ≤-1	

Table S2

Conjoint analysis among the 4 databases shows 7 commonly changed genes.

	Gene name
1	*S100A11*
2	*ANXA2*
3	*LYZ2*
4	*LGALS3*
5	*CLEC7A*
6	*LPL*
7	*SERPINA12*

Table S3

The interacting proteins with S100A11 identified by IP-MS

Number	Description	Protein Name	Protein Accession Number
1	Annexin A1	Anxa1	P10107

2	Protein arginine N-methyltransferase 5	Prmt5	A0A0R4J049
3	Comp protein (Fragment)	Comp	A2VCQ0
4	Fructose-bisphosphate aldolase	Aldoart1	A6ZI46
5	Laminin receptor (Fragment)	Rpsa	B2CY77
6	Solute carrier family 39 (Metal ion transporter), member 13, isoform CRA_a	Slc39a13	B2RQ45
7	Serine/arginine repetitive matrix protein 1 (Fragment)	Srrm1	F6T4M4
8	Voltage-dependent anion-selective channel protein 3	Vdac3	J3QMG3
9	Vimentin	Vim	P20152
10	ADP/ATP translocase 1	Slc25a4	P48962
11	Elongation factor 2	Eef2	P58252
12	Poly(rC)-binding protein 1	Pcbp1	P60335
13	Proteasome subunit beta type-4	Psmb4	P99026
14	UDP-glucose 6-dehydrogenase	Ugdh	Q3TJ71
15	RuvB-like helicase	Ruvbl1	Q3U1C2
16	Keratin, type II cytoskeletal 2 oral	Krt76	Q3UV17
17	Leucine-rich repeat flightless-interacting protein 1	Lrrfip1	Q3UZ39
18	Monofunctional C1-tetrahydrofolate synthase, mitochondrial	Mthfd1l	Q3V3R1
19	Igk protein	Igk	Q58EU8
20	60S acidic ribosomal protein P0	Rplp0	Q5FWB6
21	Voltage-dependent anion-selective channel protein 1	Vdac1	Q60932
22	E3 ubiquitin-protein ligase TRIM21	Trim21	Q62191
23	AHNAK (Fragment)	Ahnak	Q6UL10
24	Heat shock protein 84b	Hsp90ab1	Q71LX8
25	Filamin-A	Flna	Q8BTM8
26	Leucine-rich repeat transmembrane neuronal protein 3	Lrrtm3	Q8BZ81
27	Ras-related protein Rab-5C	Rab5c	Q8C266
28	Isocitrate dehydrogenase [NADP]	Idh1	Q8C338
29	Histone deacetylase	Hdac6	Q8CGC3

Table S4

Information of antibodies used in this study

Antibody	Supplier	Catalog number
S100A11	Proteintech	10237-1-AP
S100A11	Proteintech	60024-1-IG
SREBP2	Abcam	Ab30682
GFP	Abcam	ab290
FLAG	Sigma-Aldrich	F3165
KPNB	Abcam	Ab2811
KPNB	CST	60769S
HMGCR	Proteintech	13533-1AP
HMGCR	Abcam	Ab174830
LDLR	Proteintech	10785-1-AP
F4/80	CST	70076S
ANXA1	Proteintech	21990-1-AP
S1P	Santa Cruz	sc-271916
NLRP3	CST	15101S
IL1B	CST	12242
Caspase1	CST	4199
Caspase1	CST	89932
Caspase2	Proteintech	10436-1-AP
BIP	Proteintech	66574-1-LG
GAPDH	Proteintech	60004-1-Ig
ACTIN	Proteintech	66009-1-IG
S2P	Santa Cruz	sc-293341

Table S5

Information on Primers Used in This Study

Primer symbols	Primer sequences (5'-3')	
	Forward	Reverse
S100A11	ATGGCAAAAATCTCCAG CCCTAC	CGCTCAGGTCCGCTTCTG GGAAG
S100A11(K36I)	CTCTCCATCACAGAGTTC CTAAG	GGAACTCTGTGATGGAGA GAGTGTAG
S100A11(D61G)	GTCCTTGGCCGCATGAT GAAGAAAC	CTTCATCATGCGGCCAAG GACACCAGGGTC
S100A11(N82G)	GAATTTCTTGGCCTGATT GGTGGCCTAGC	CCAATCAGGCCAAGAAA TTCTGAG
S100A11(G86T)	GATTGGTACCCTAGCTA TGGCTTGCCATG	GCCATAGCTAGGGTACCA ATCAGATTAAGAAATTC
S100A11(N)	ATGGCAAAAATCTCCAG CCCTAC	CTTCTGGAAGACAGCAAT CAGGGAC

S100A11(C)	ATGCTAGCTATGGCTTGCCATG	GGTCCGCTTCTGGGAAGGGA
S100A11(ΔN)	ATGTATGCTGGAAAGGATGGT	CGCTCAGGTCCGCTTCTGGGAAG
S100A11(ΔC)	ATGGCAAAAATCTCCAGCCCTAC	GCCACCAATCAGATTAAGAAA
KPNB	ATGGAGCTCATAACCATCCTCG	AGCCTGGTTCTTCAGTTTCCTC
ANXA1	ATGGCAATGGTATCAGAATT	GTTTCCTCCACAAAGAGCCA
ANXA1(N)	ATGGCAATGGTATCAGAATT	ATTGAAGGTAGGATAGGGGC
ANXA1(C1)	CCATCCTCGGATGTCGCTGC	AGTTTTTAGCAGAGCTAAAA
ANXA1(C2)	CCAGCGCAATTTGATGCTGA	ATTCACACCAAAGTCCTCAG
ANXA1(C3)	GAAGACTTGGCTGATTCAGA	GCTTGTGGCGCACTTCACGA
ANXA1(C4)	AAACCAGCTTTCTTTGCAGA	GTTTCCTCCACAAAGAGCCA
CASPASE2	ATGCATCCTCATCATCAGGAAA	TCATAGAGCAAGAGAGGCGGTG
