# Supplementary material for: S100A11 regulates hepatic cholesterol metabolism and promotes steatohepatitis via noncanonical SREBP2 signaling
Source: J Clin Invest. 2026 Aug 4;136(19):e201406. doi: 10.1172/JCI201406 (PMC13626821; doi:10.1172/JCI201406)

Full unedited blot/gel for Figure 1F.

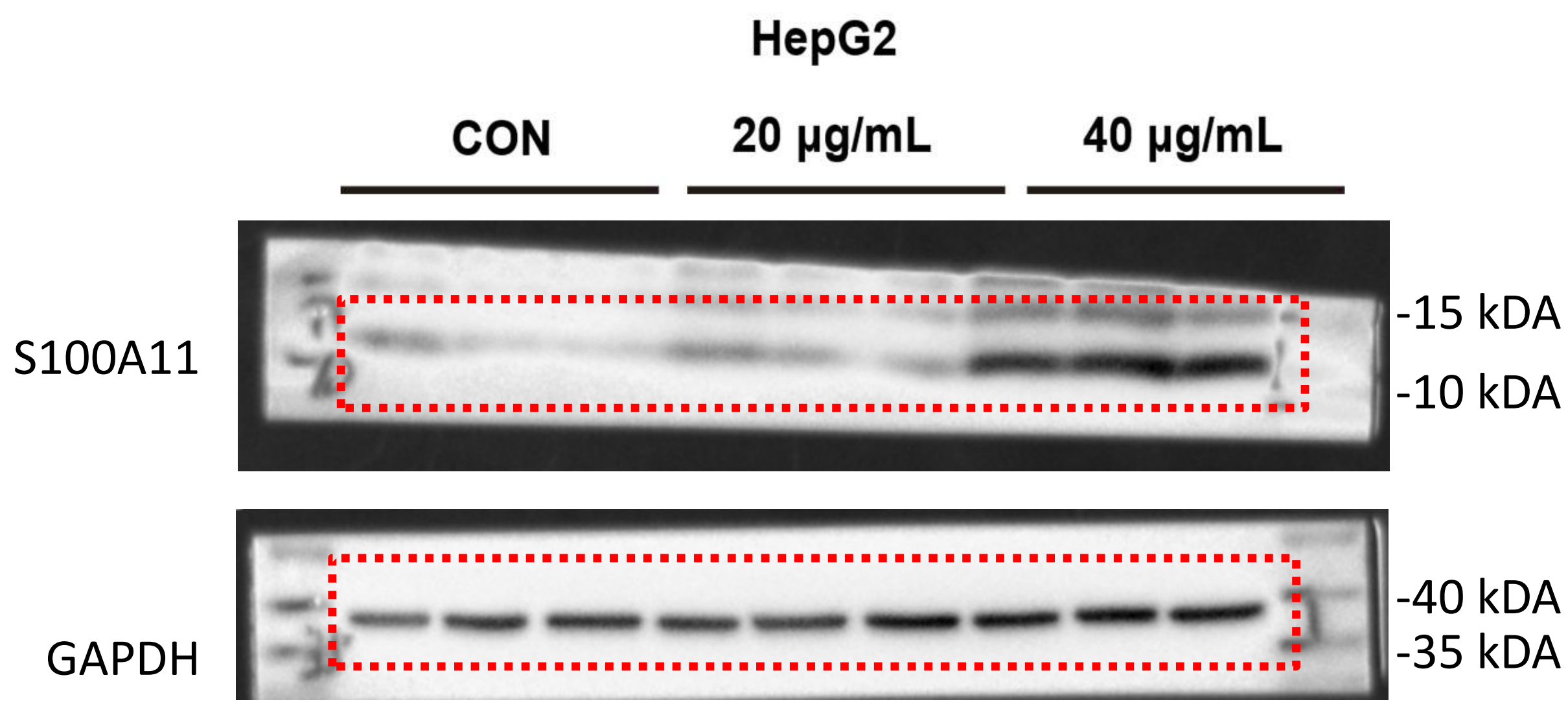

Full unedited blot/gel for Figure 1G.

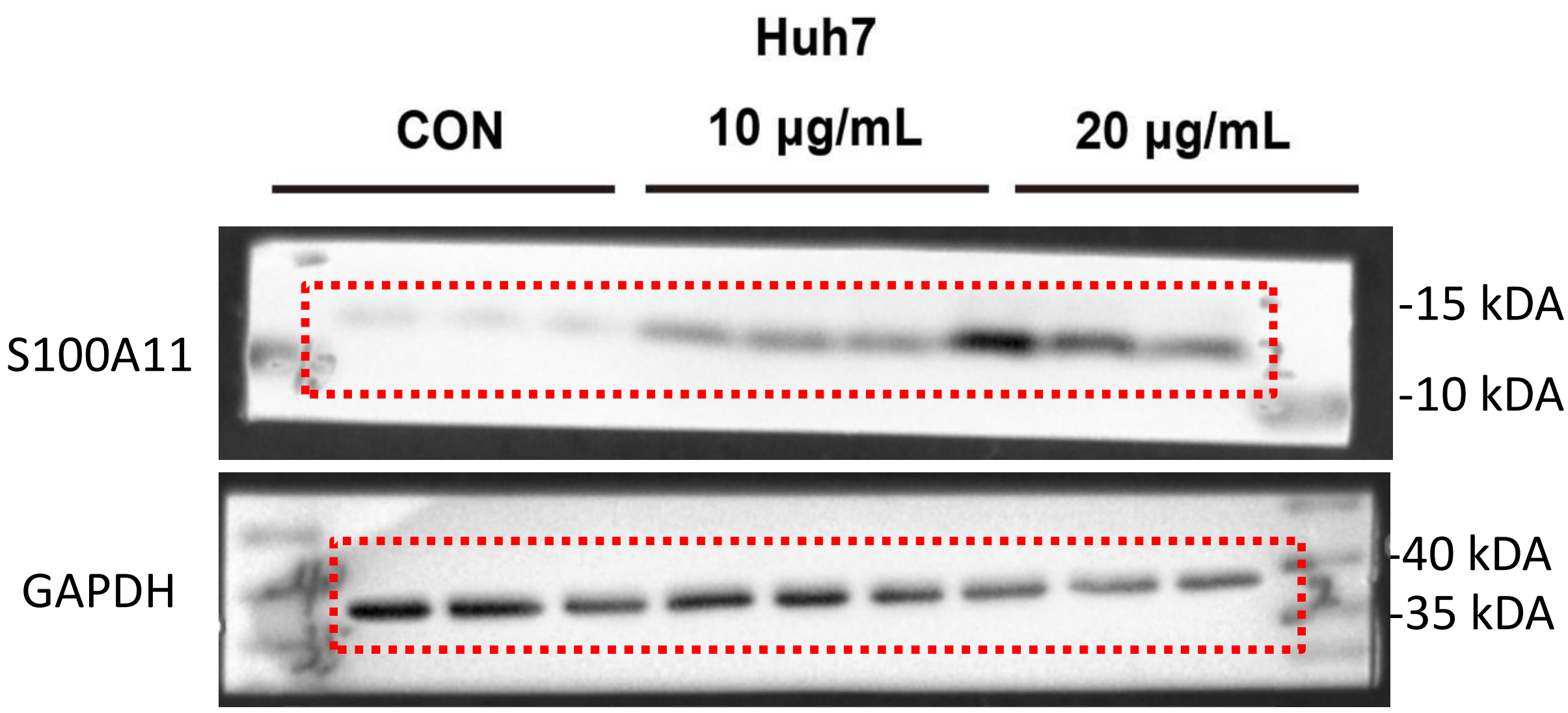

Full unedited blot/gel for Figure 1H.

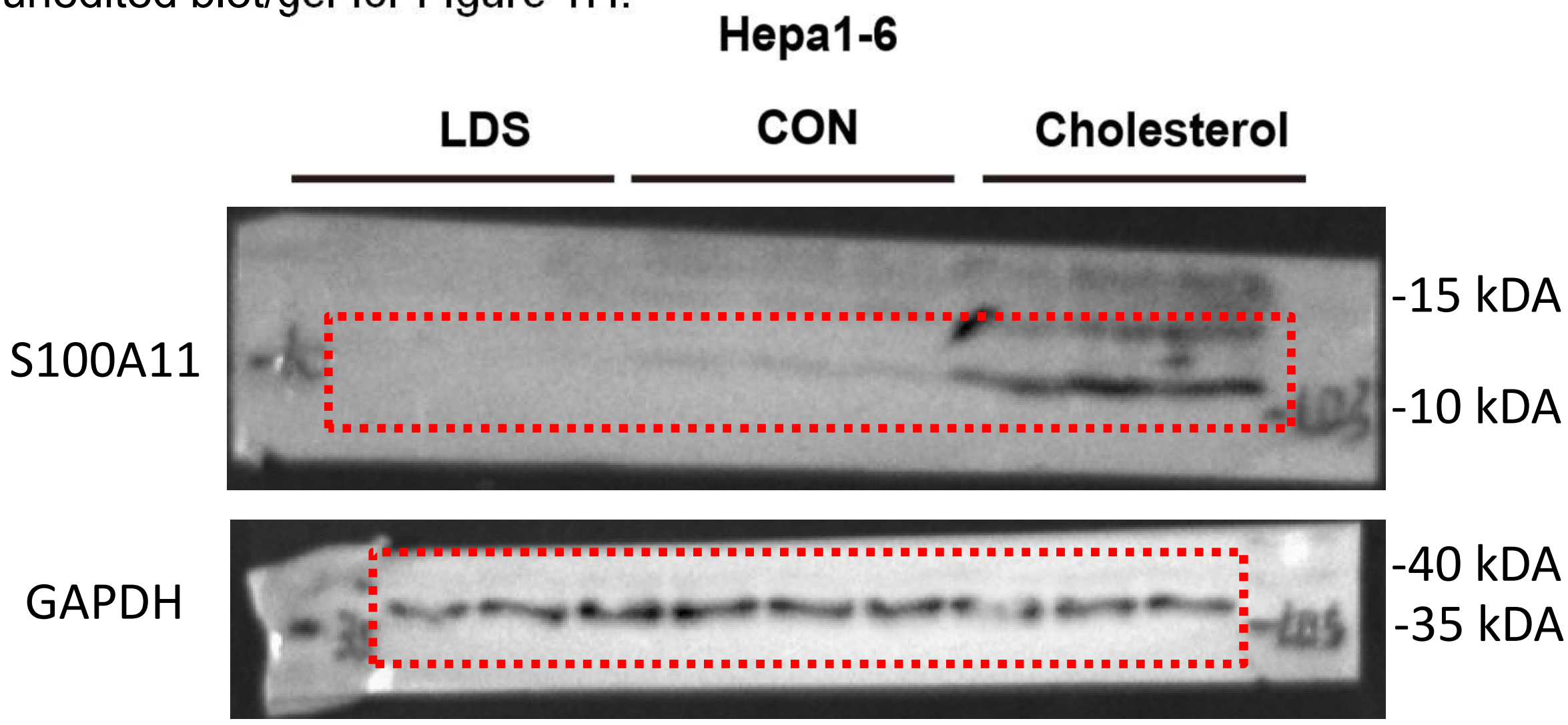

Full unedited blot/gel for Figure 1J.

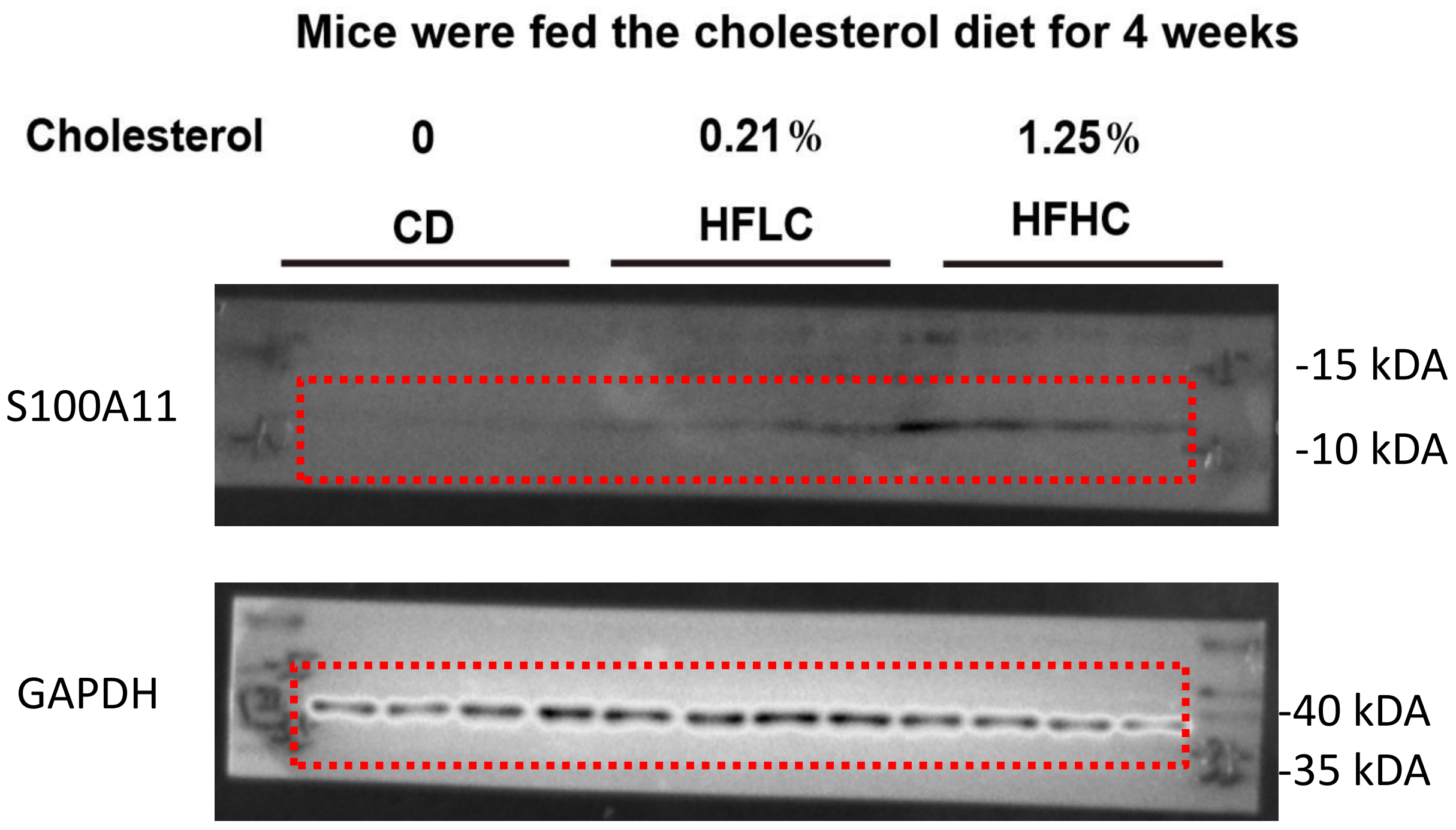

Full unedited blot/gel for Figure 1K.

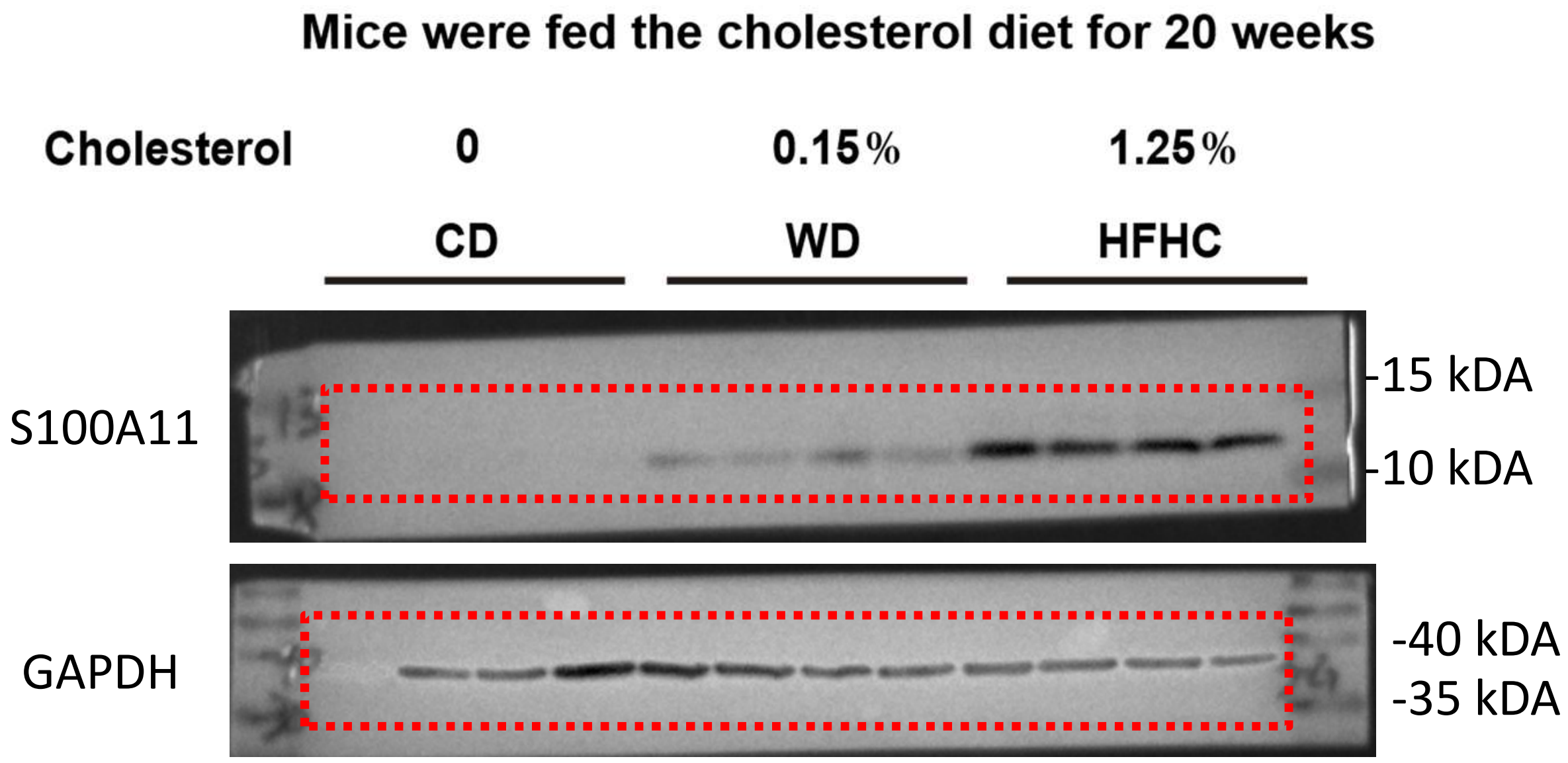

Full unedited blot/gel for Figure 1L.

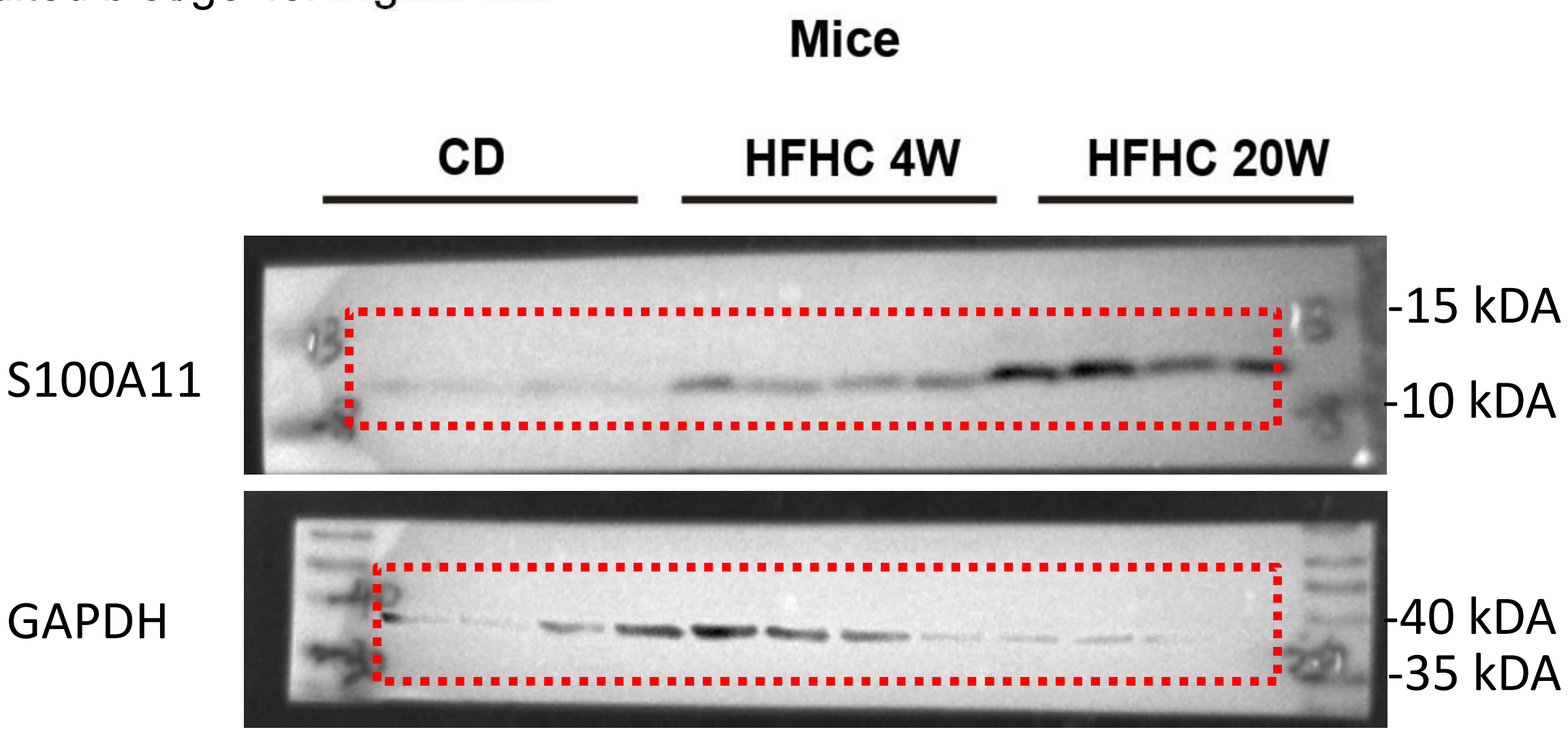

Full unedited blot/gel for Figure 2F.

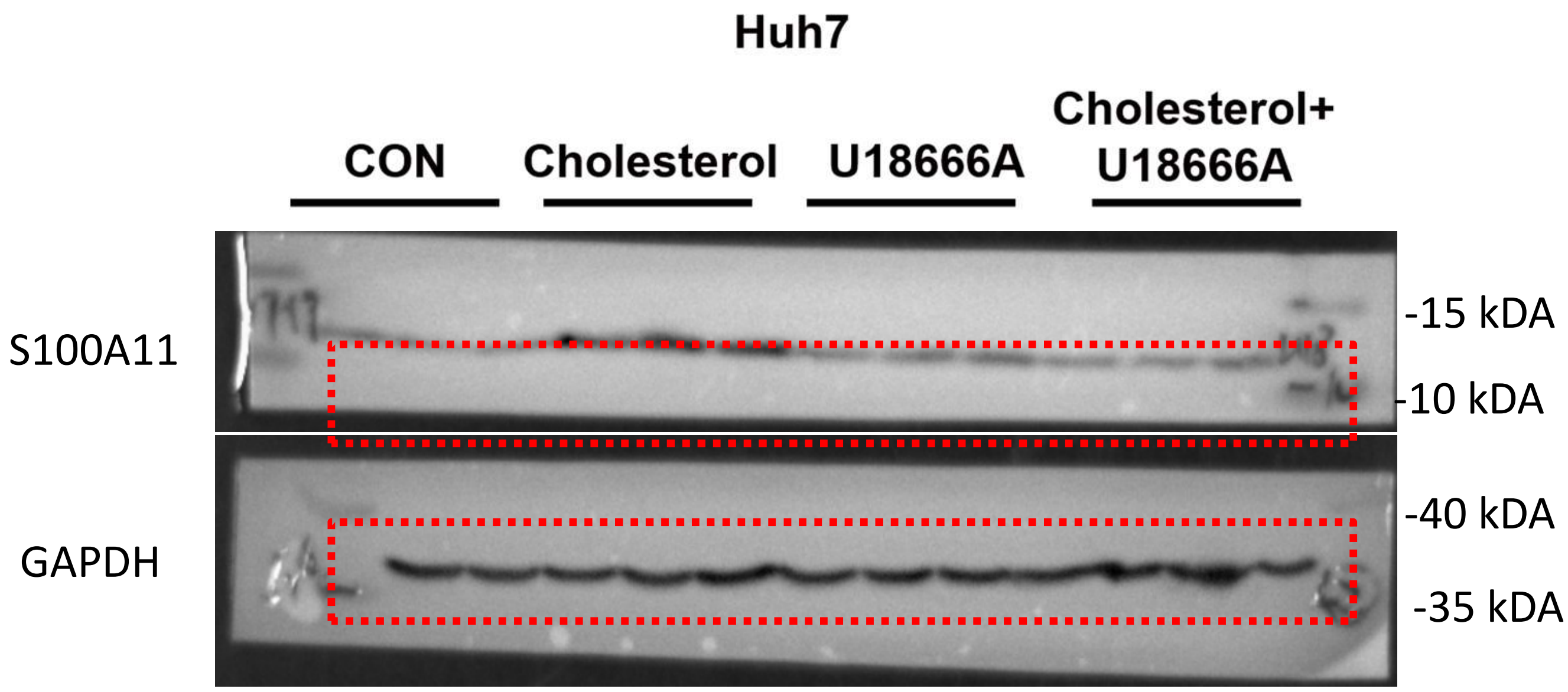

Full unedited blot/gel for Figure 2G.

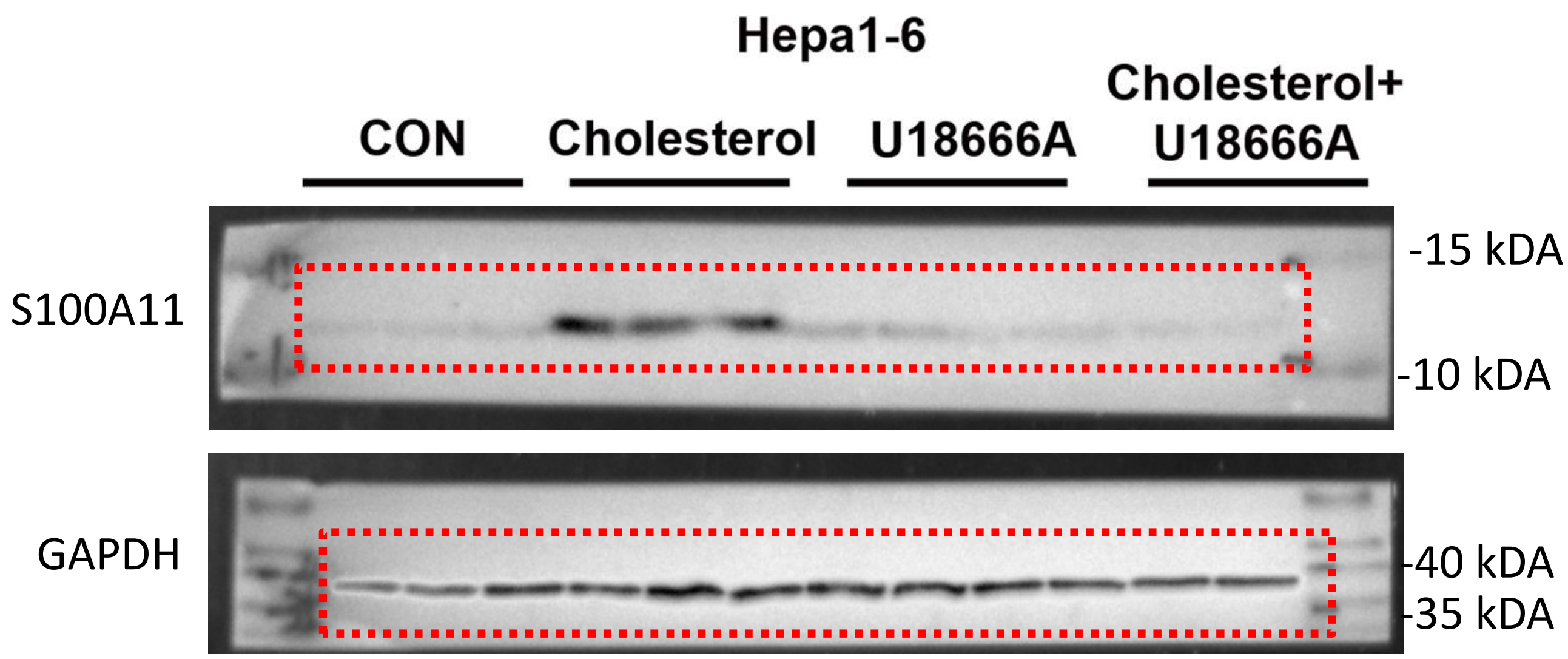

Full unedited blot/gel for Figure 2I.

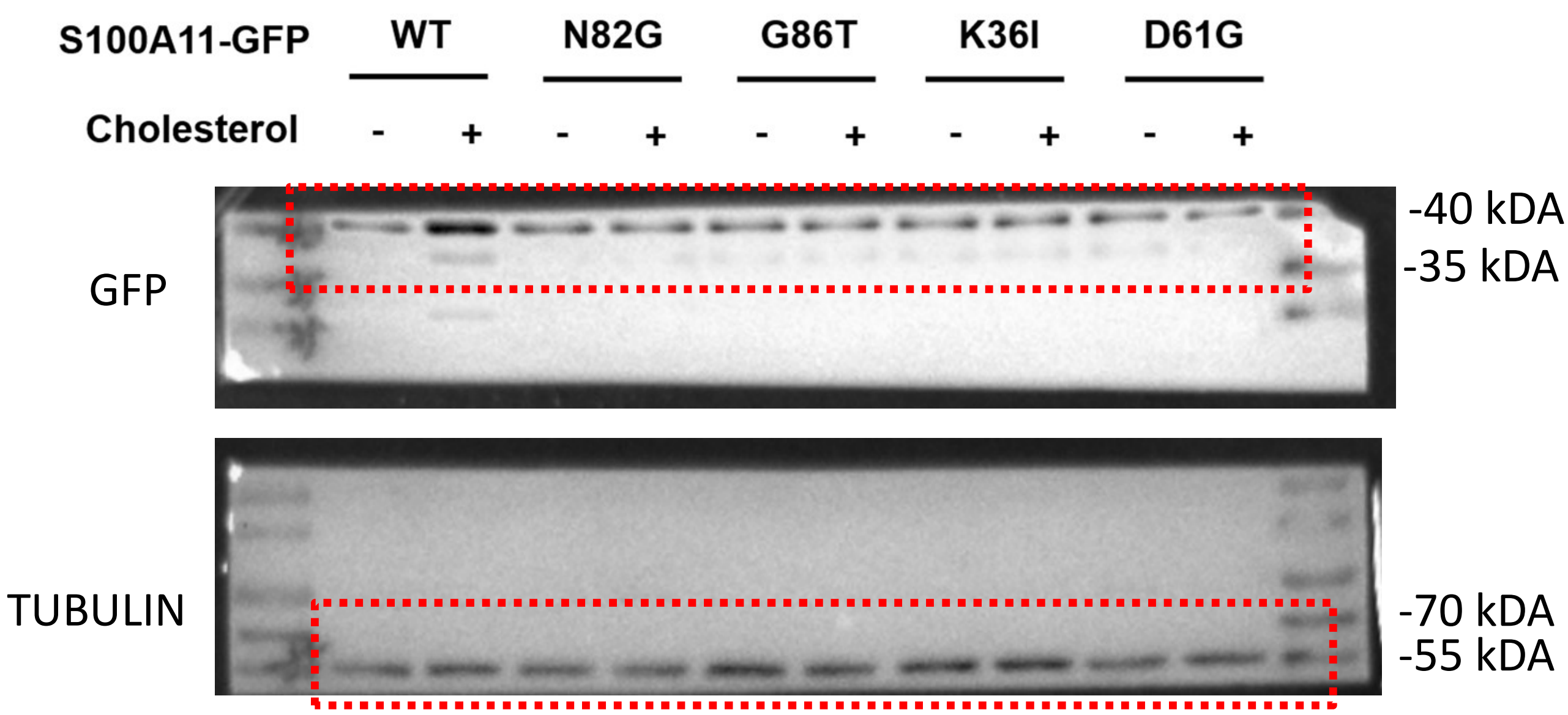

Full unedited blot/gel for Figure 3A.

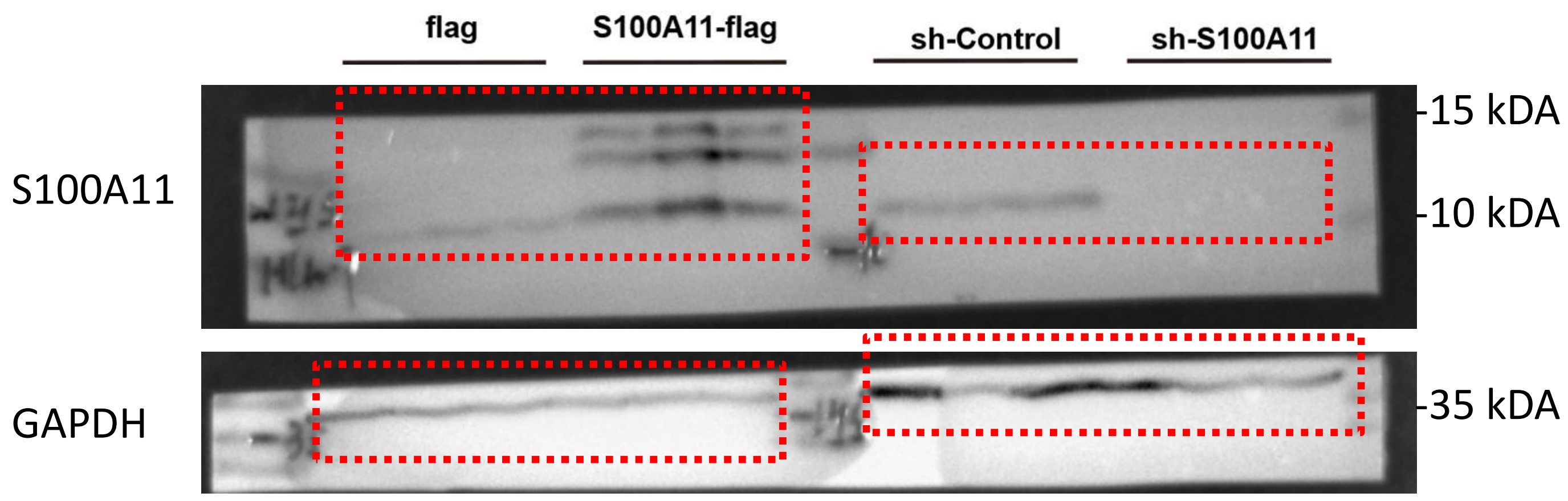

Full unedited blot/gel for Figure 3B.

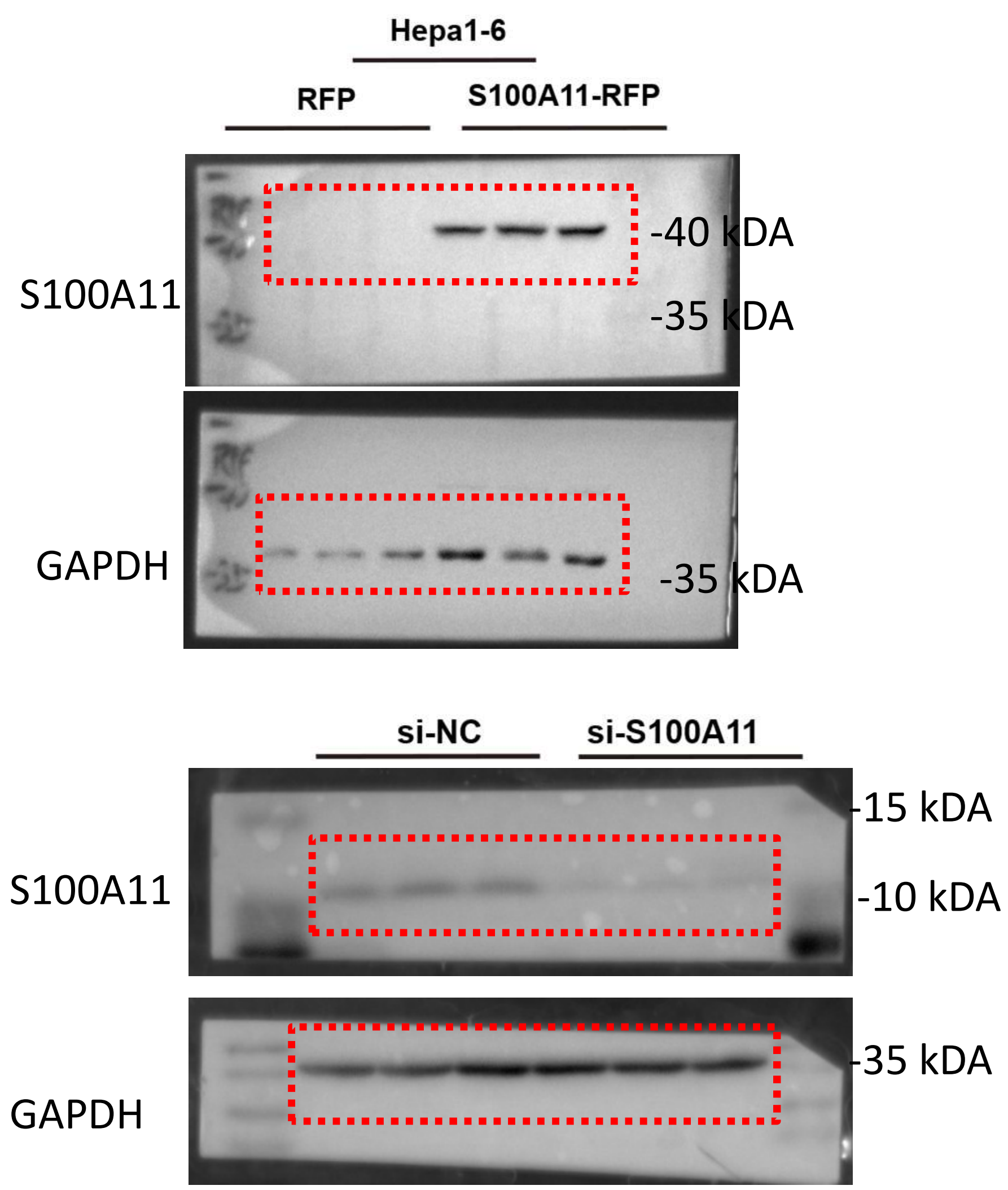

Full unedited blot/gel for Figure 3G and I

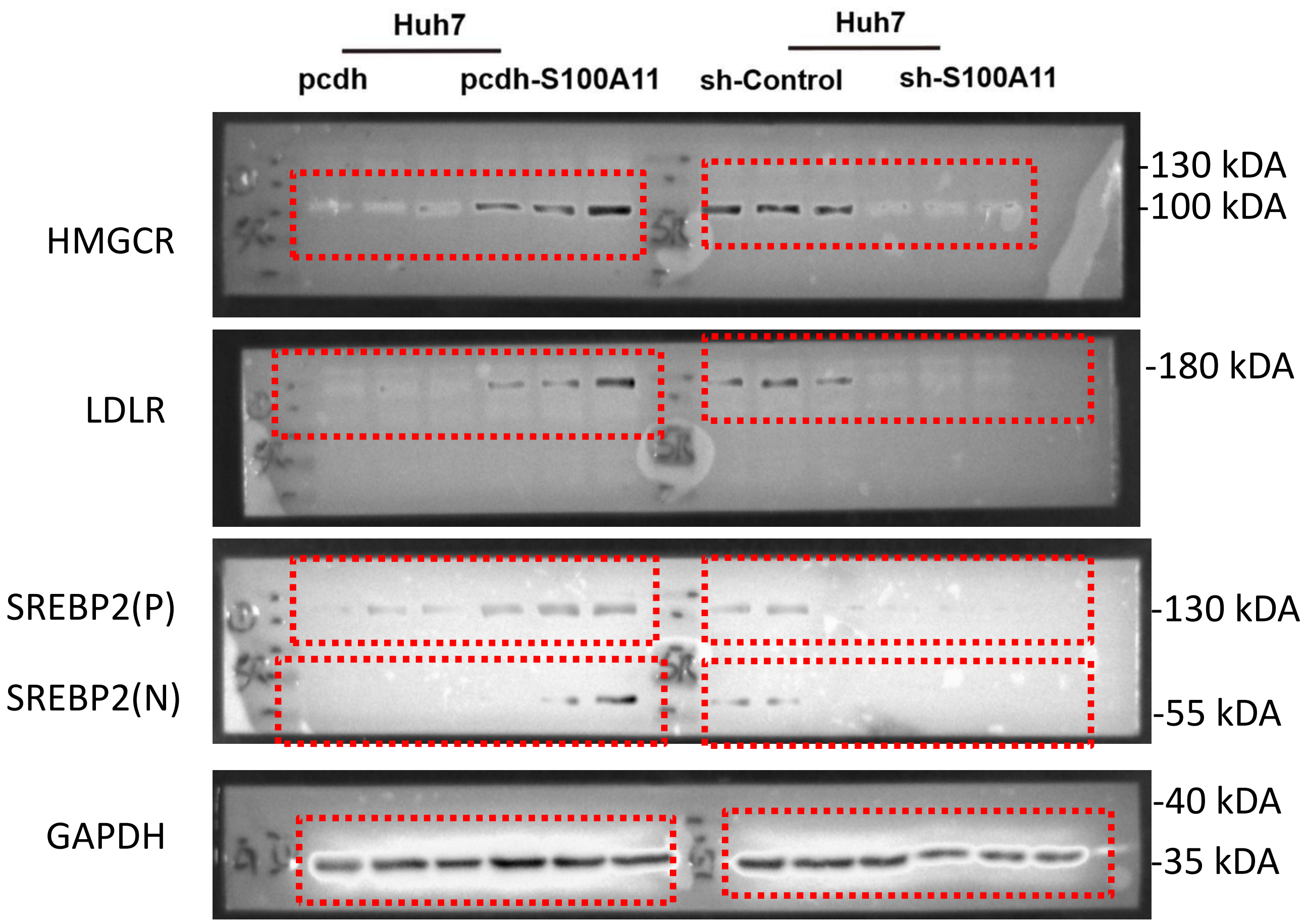

Full unedited blot/gel for Figure 3H and J

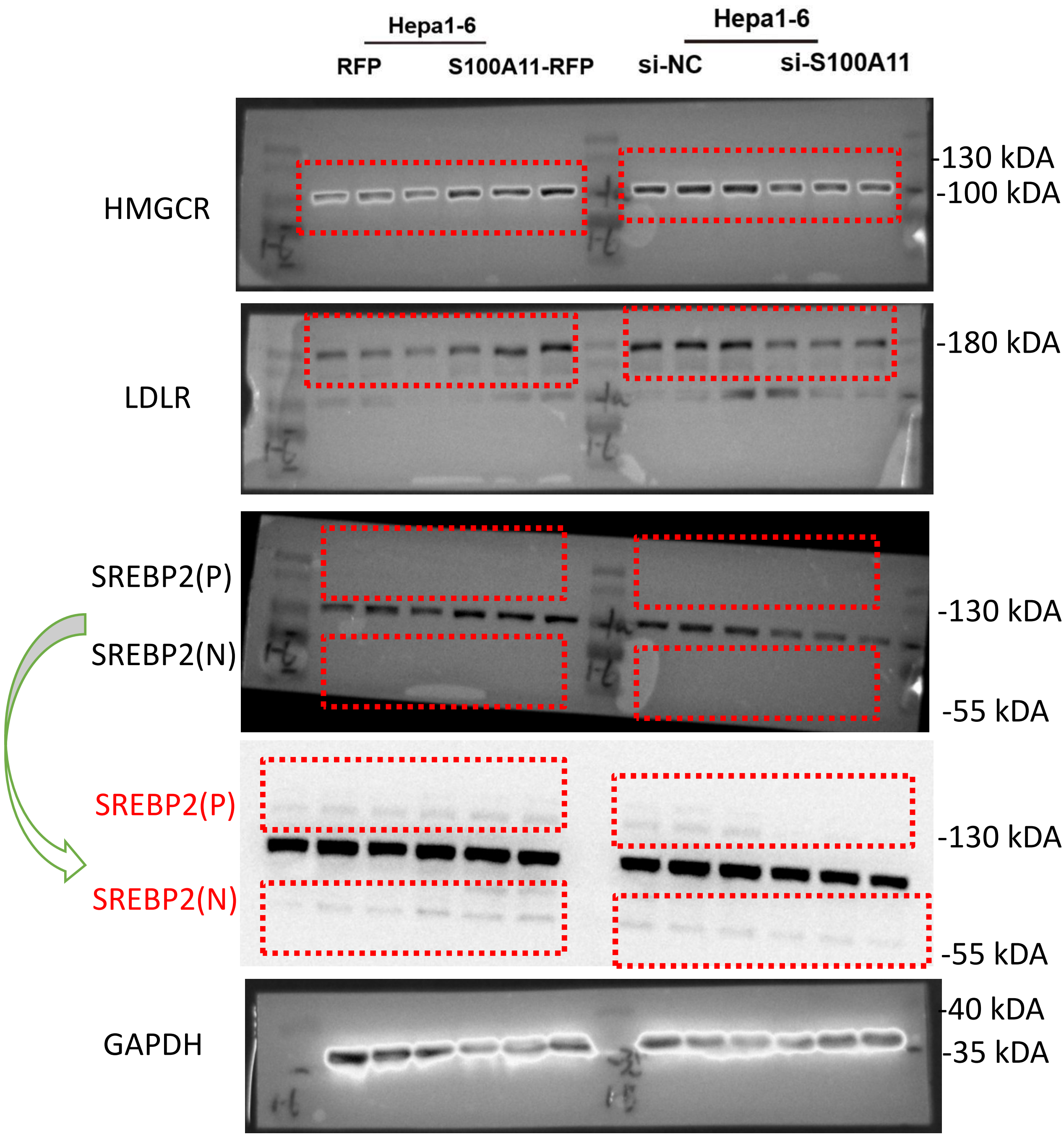

Full unedited blot/gel for Figure 4H

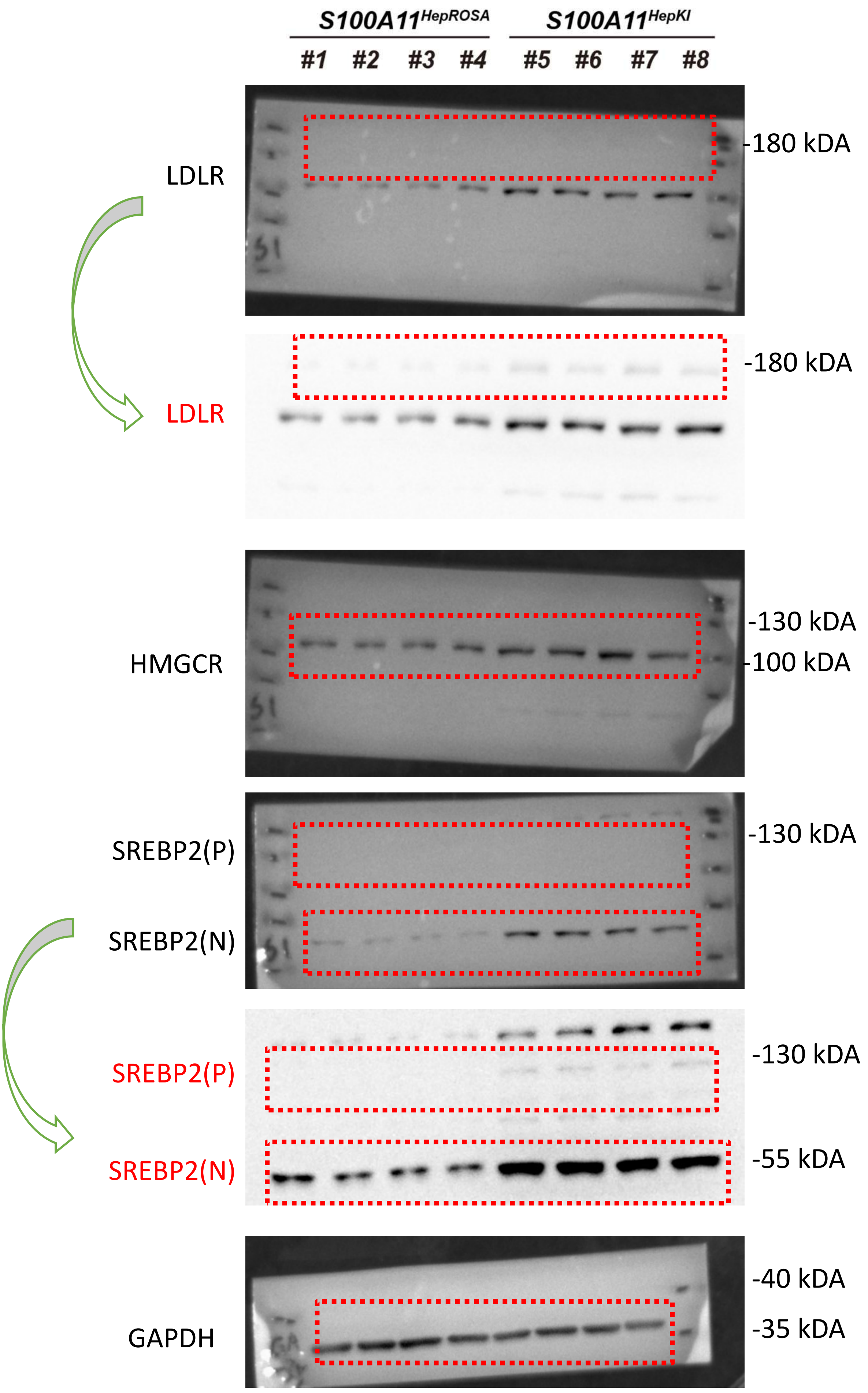

Full unedited blot/gel for Figure 4K

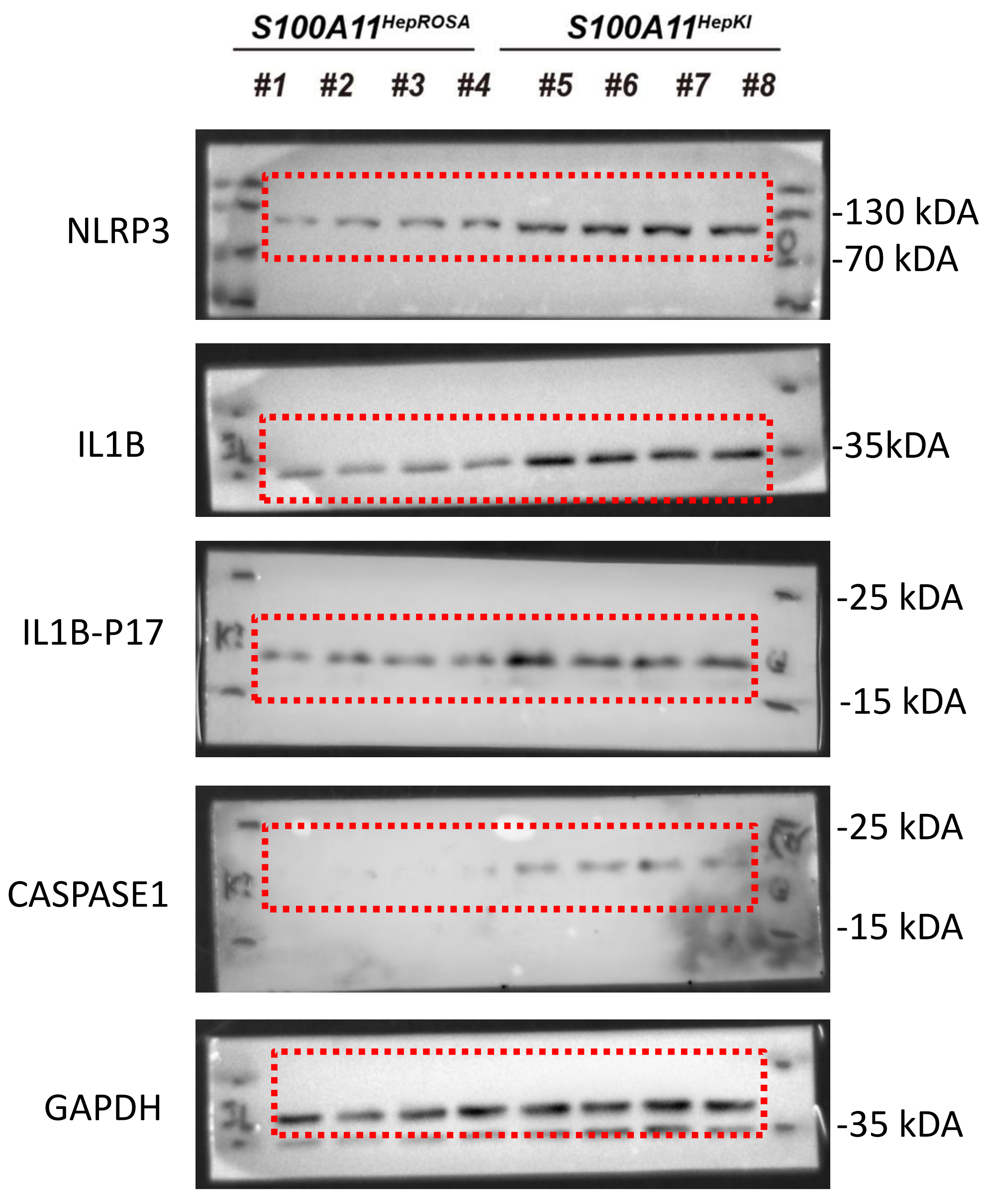

Full unedited blot/gel for Figure 5H

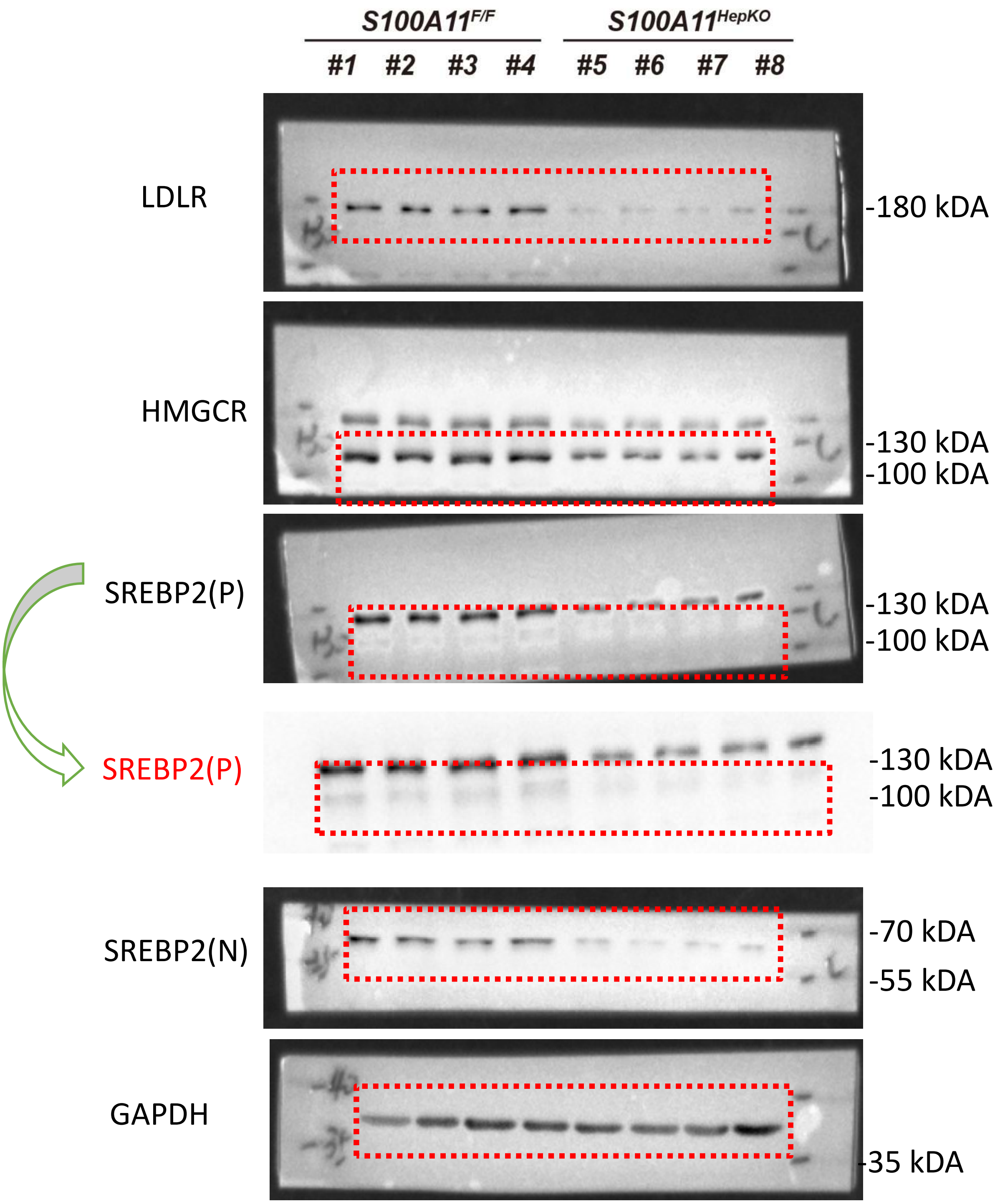

Full unedited blot/gel for Figure 5J

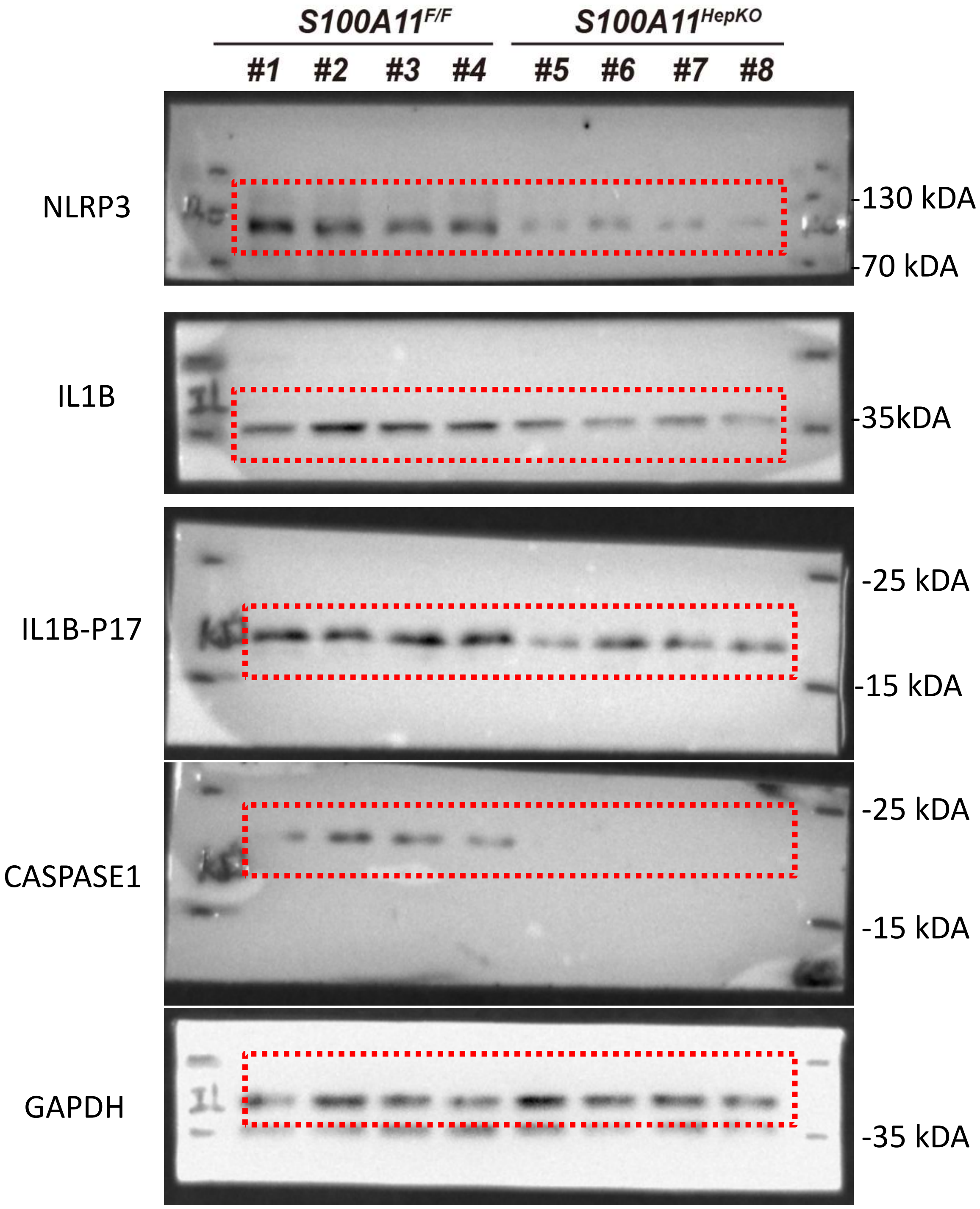

Full unedited blot/gel for Figure 6A

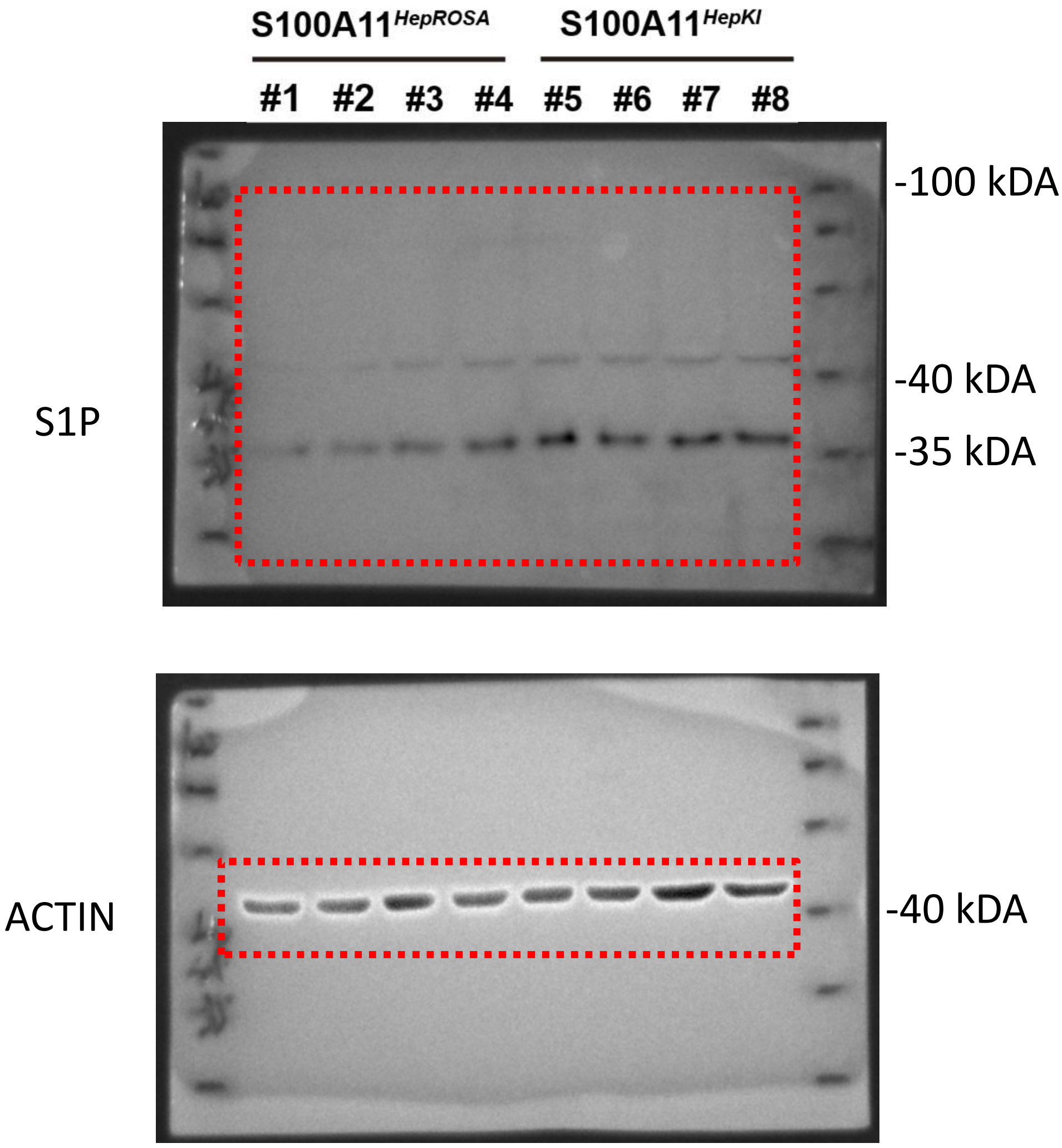

Full unedited blot/gel for Figure 6B

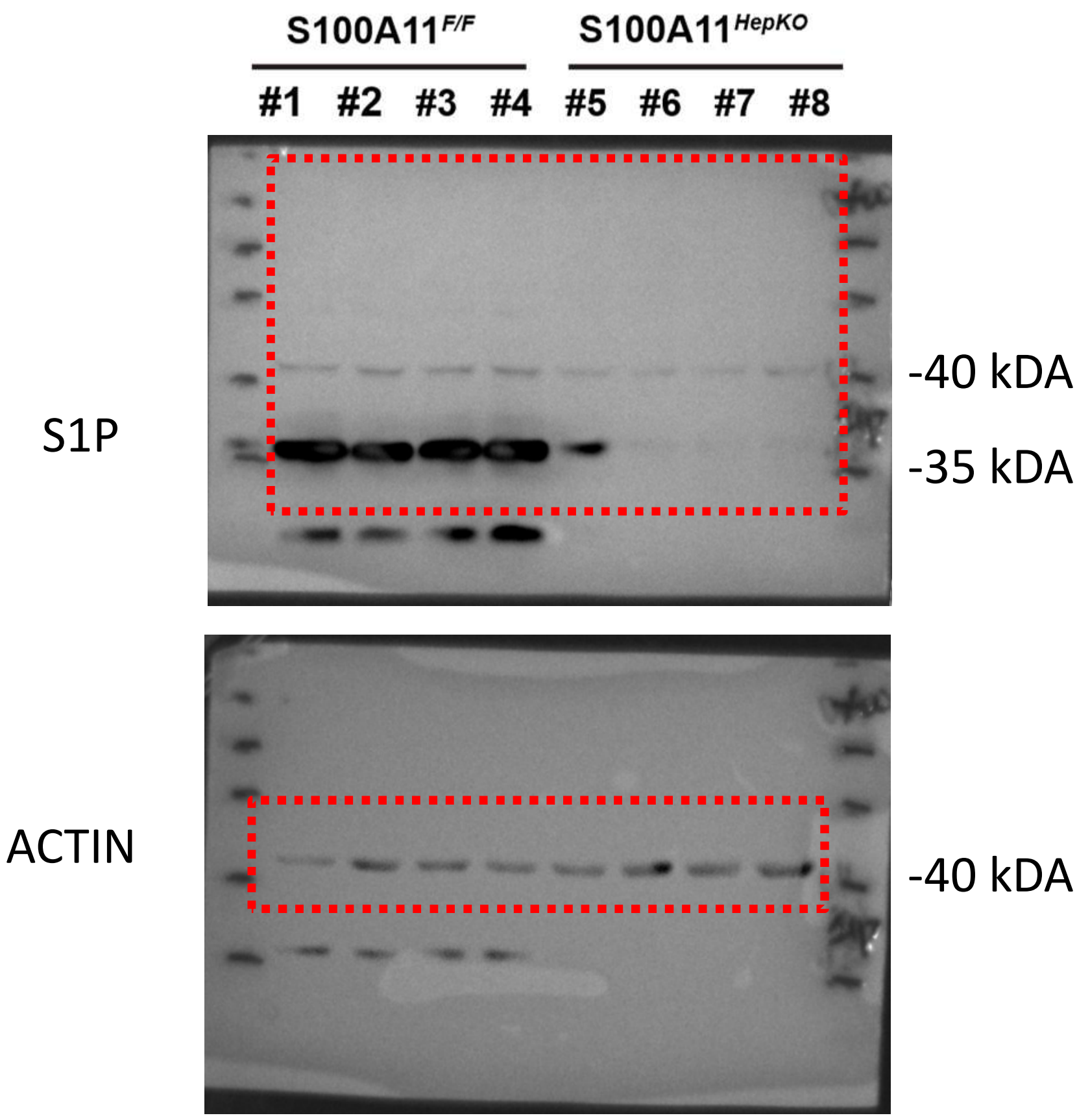

Full unedited blot/gel for Figure 6C

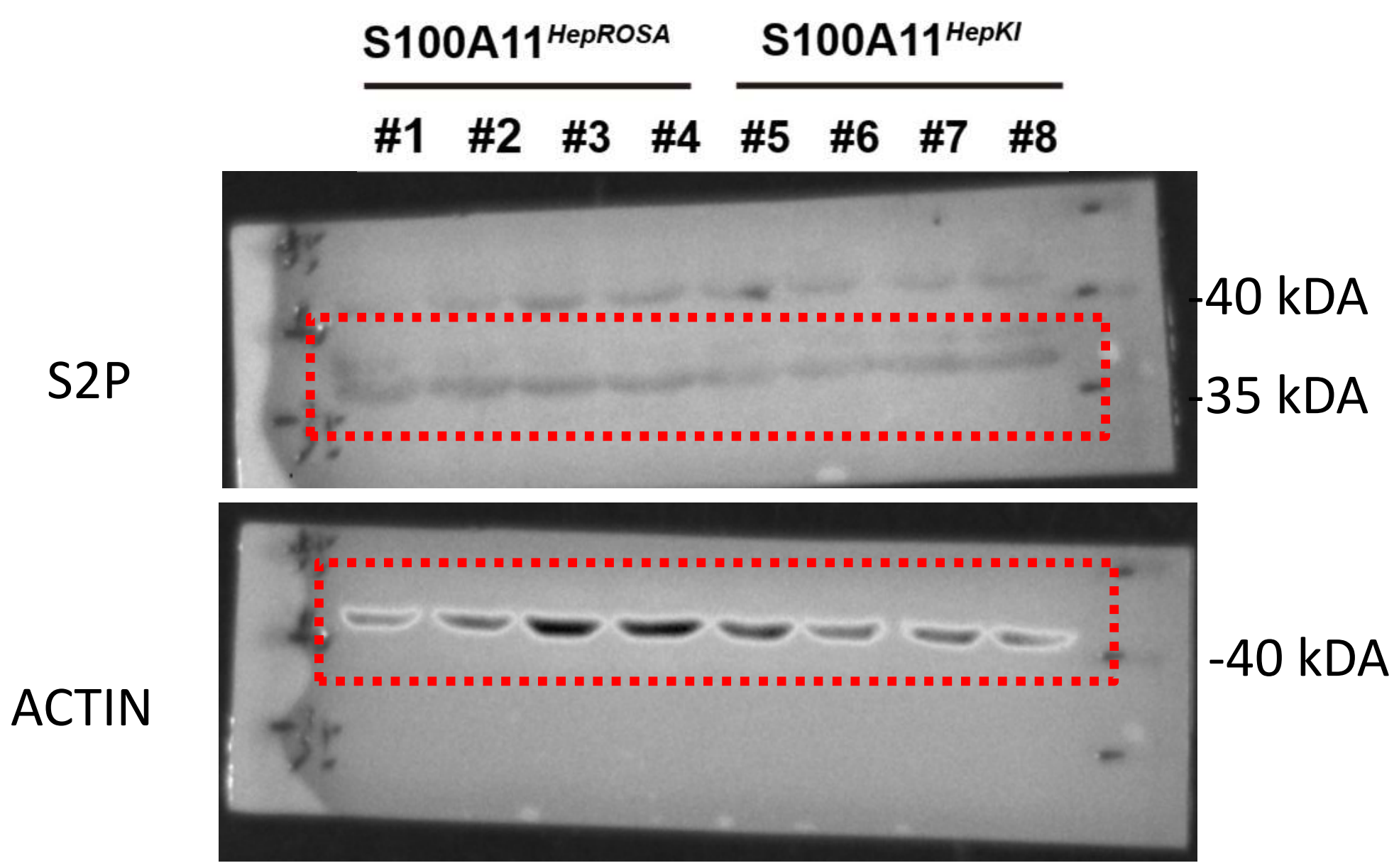

Full unedited blot/gel for Figure 6D

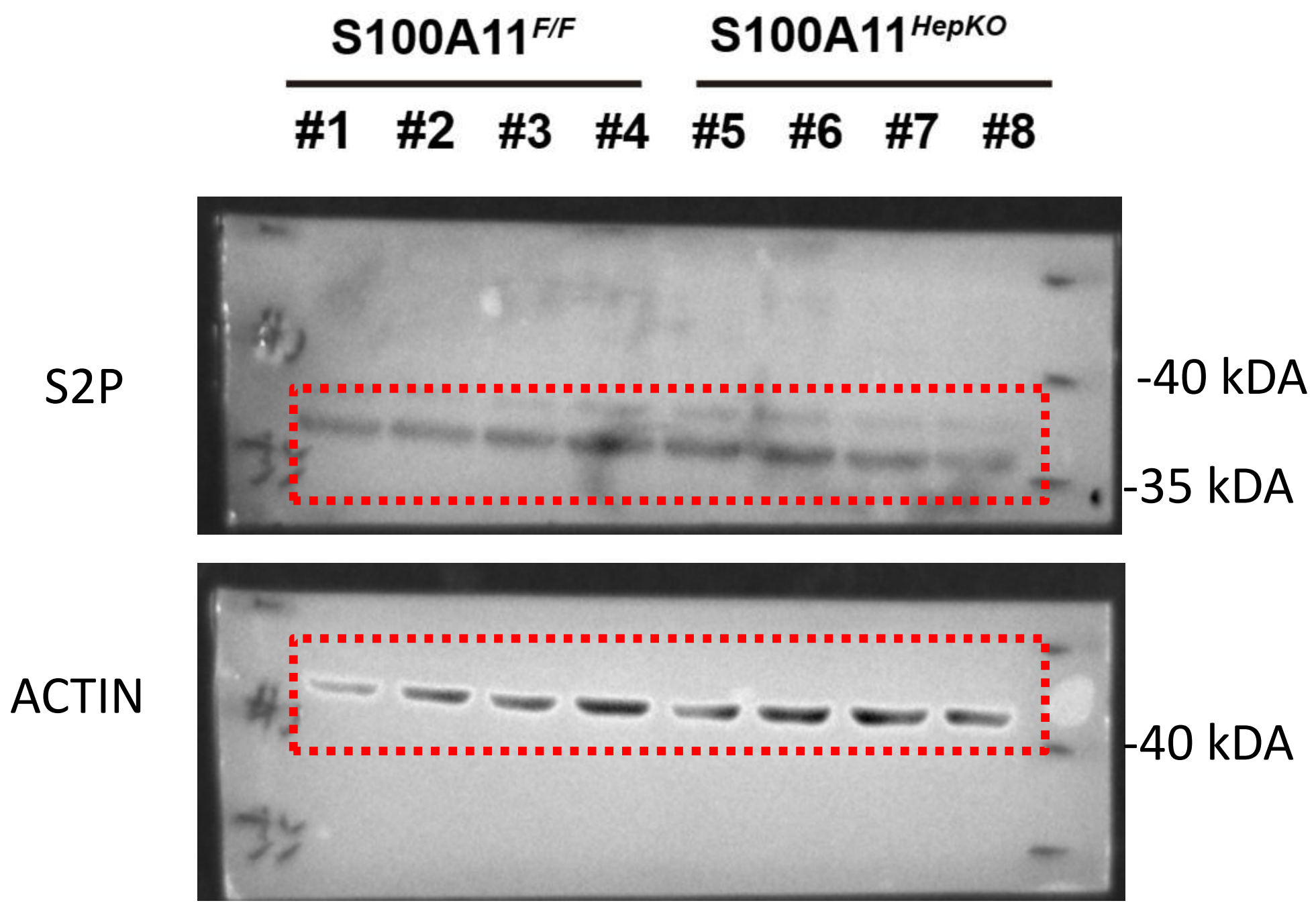

Full unedited blot/gel for Figure 6F

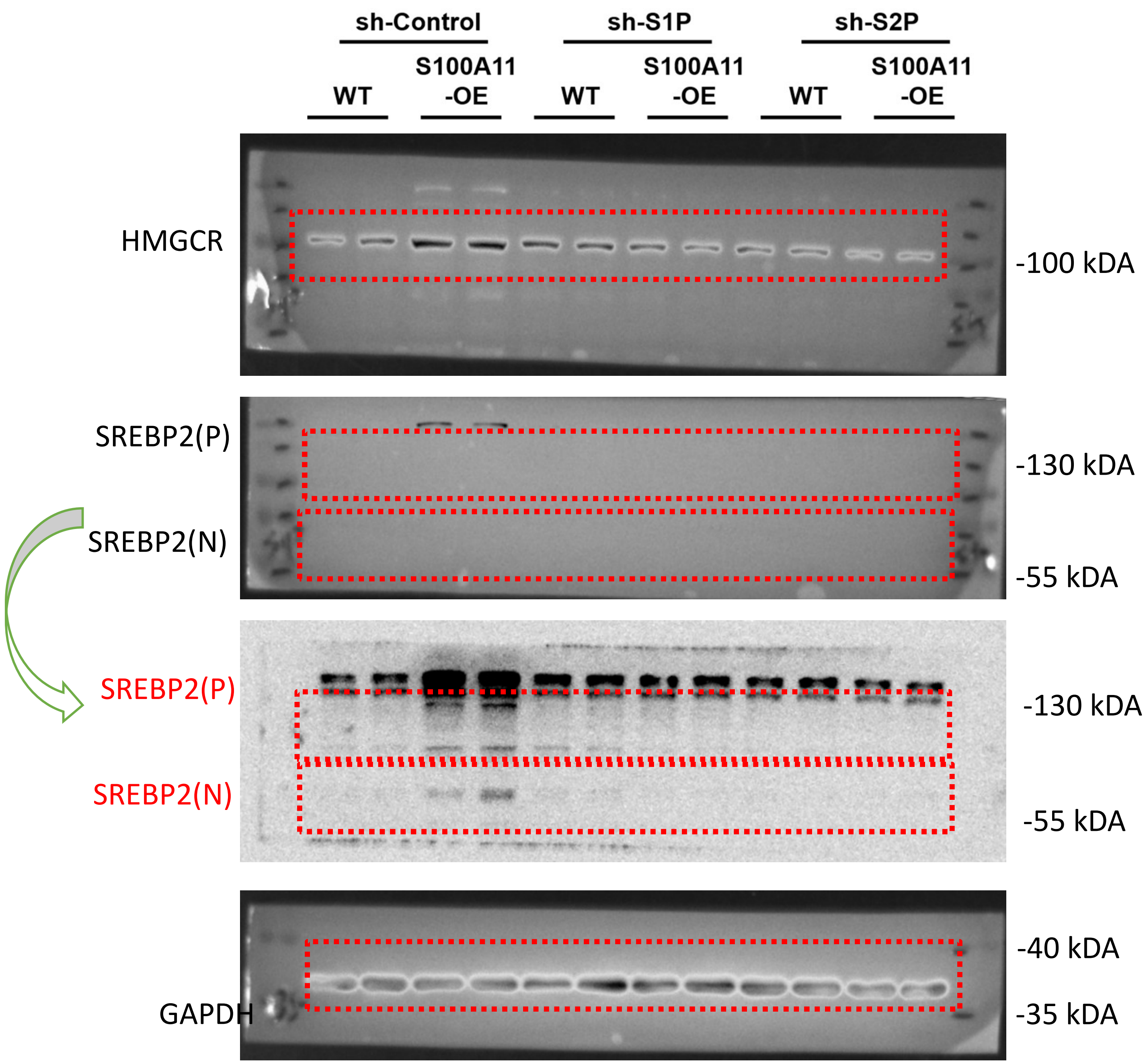

Full unedited blot/gel for Figure 6H

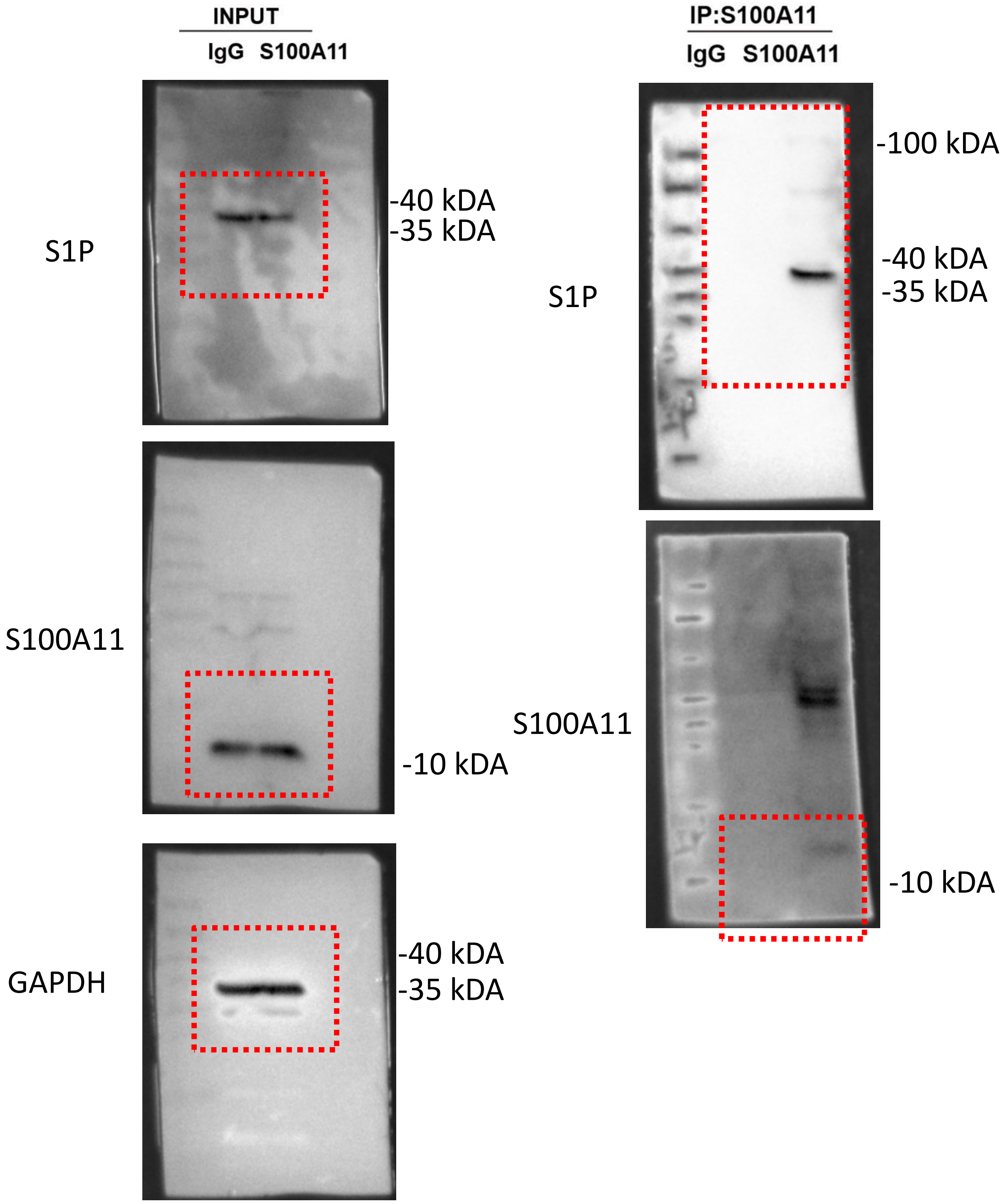

Full unedited blot/gel for Figure 6I

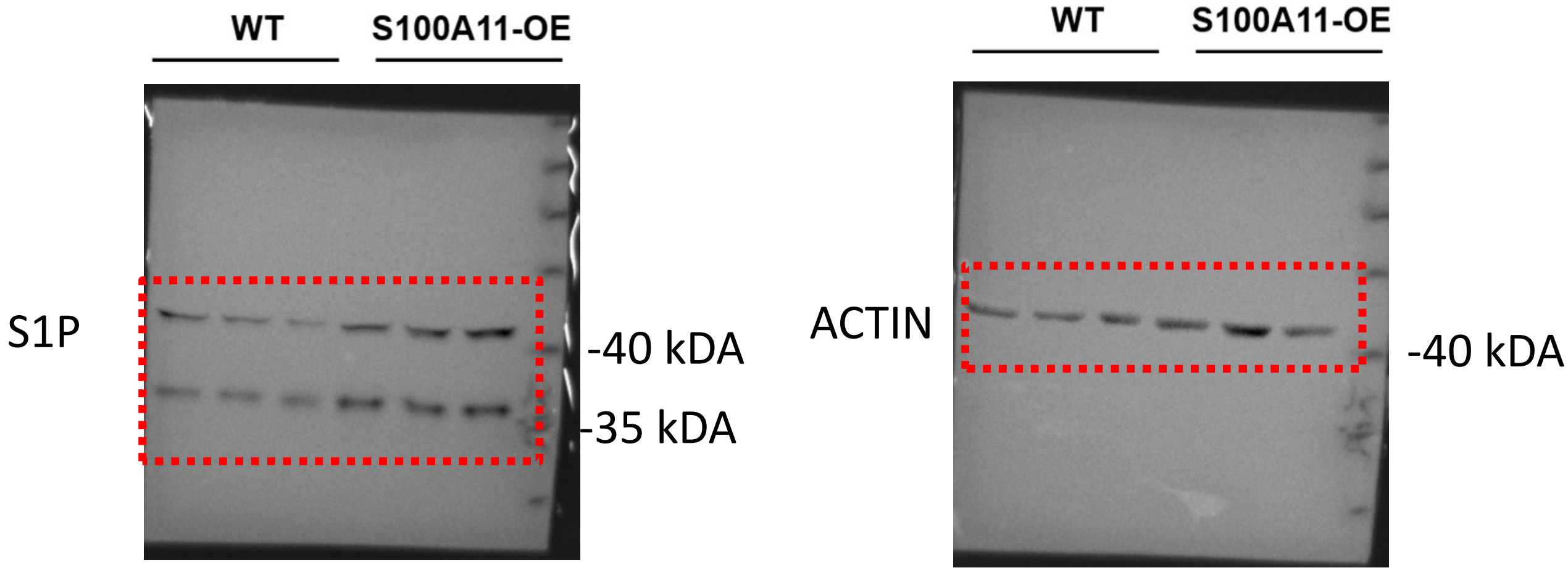

Full unedited blot/gel for Figure 6J

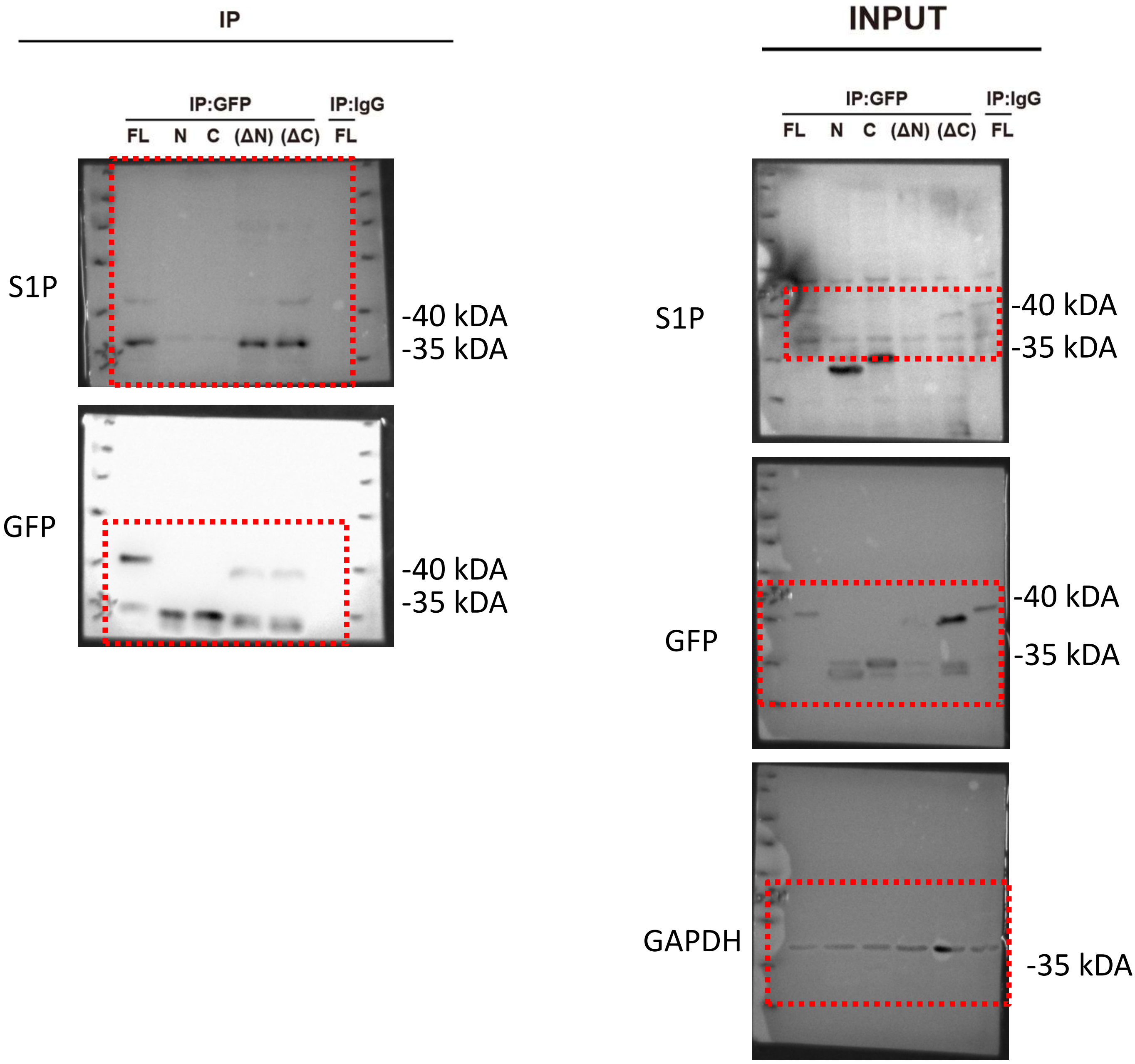

Full unedited blot/gel for Figure 6L

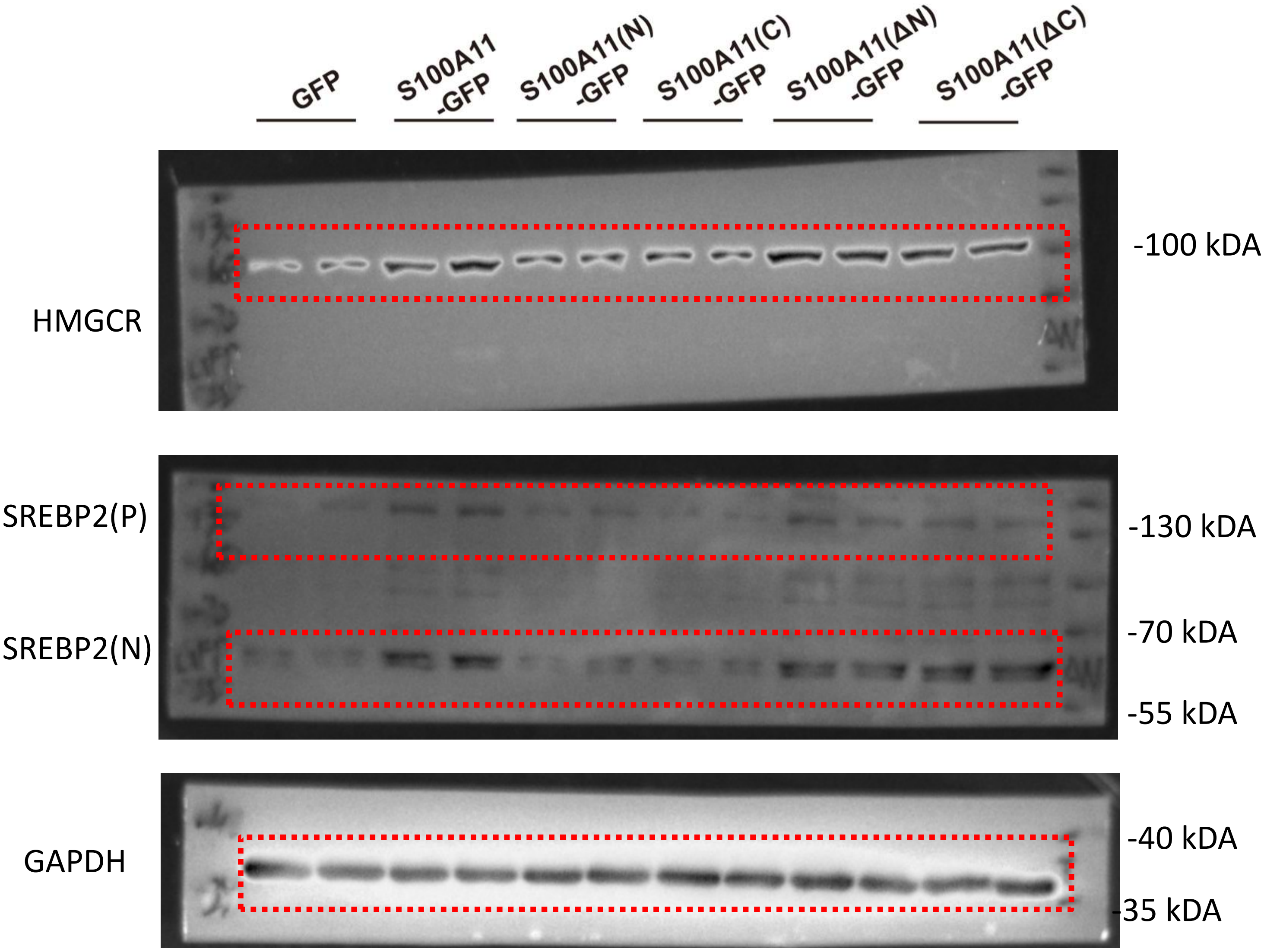

Full unedited blot/gel for Figure 7A

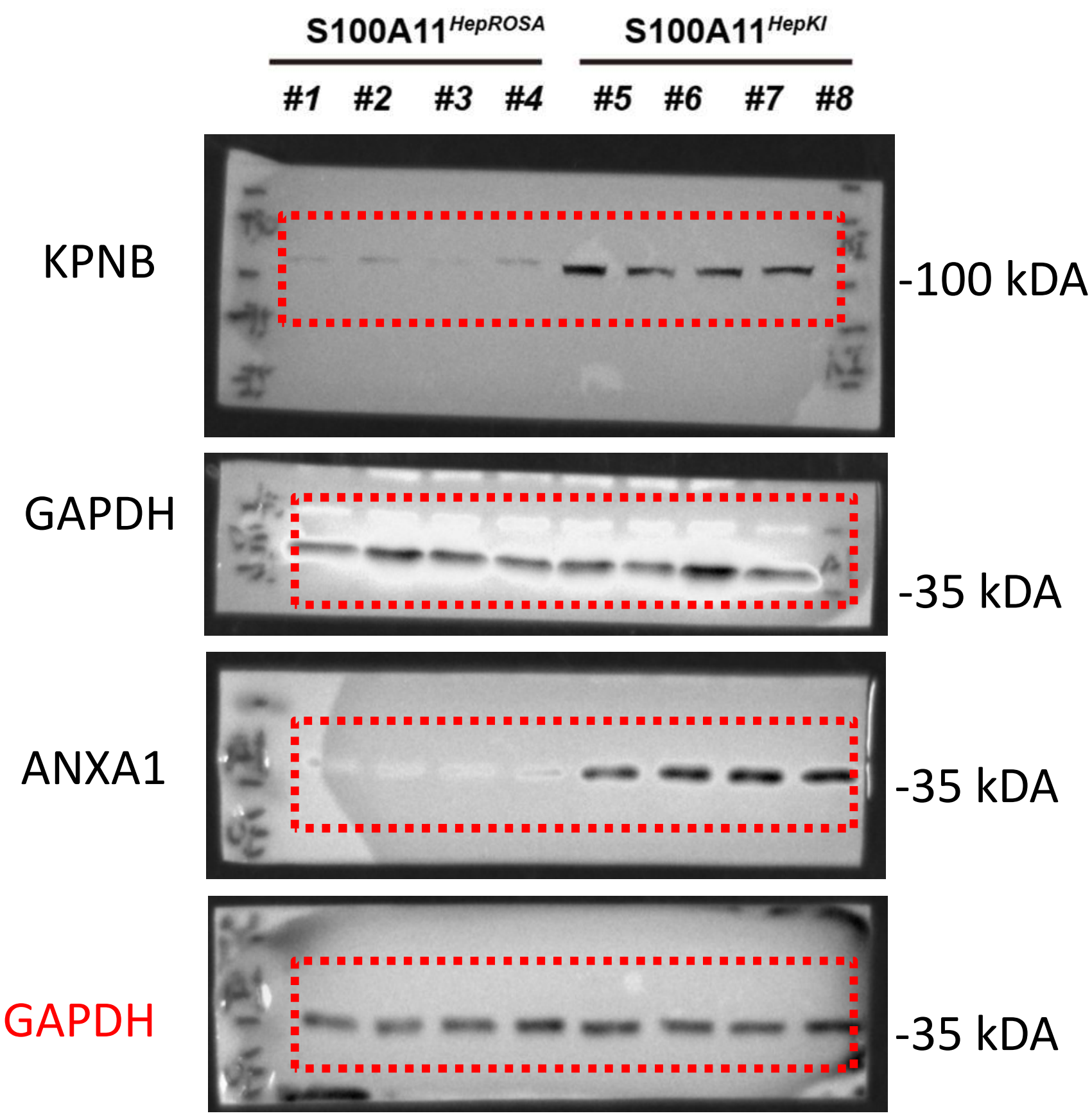

Full unedited blot/gel for Figure 7B

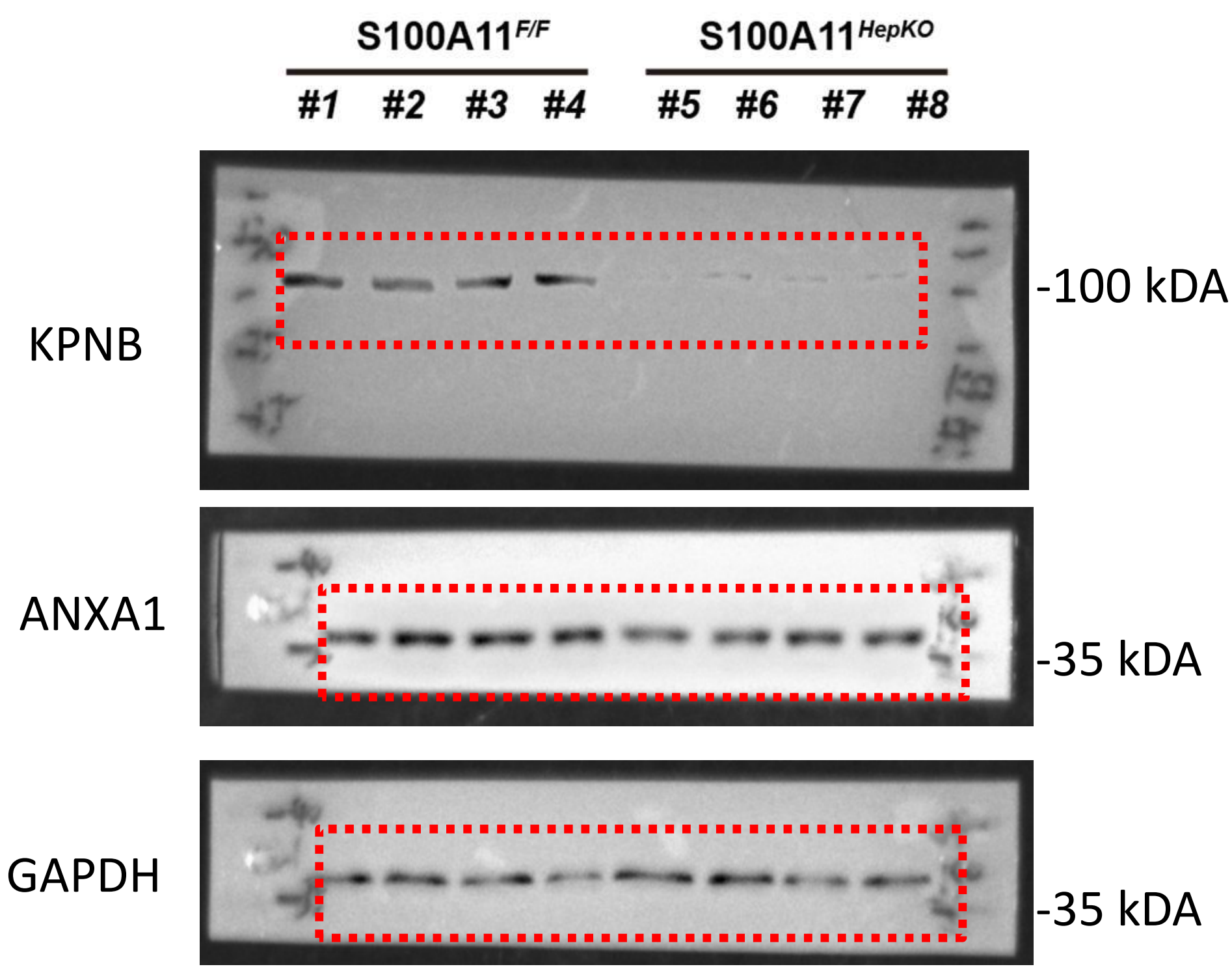

Full unedited blot/gel for Figure 7C

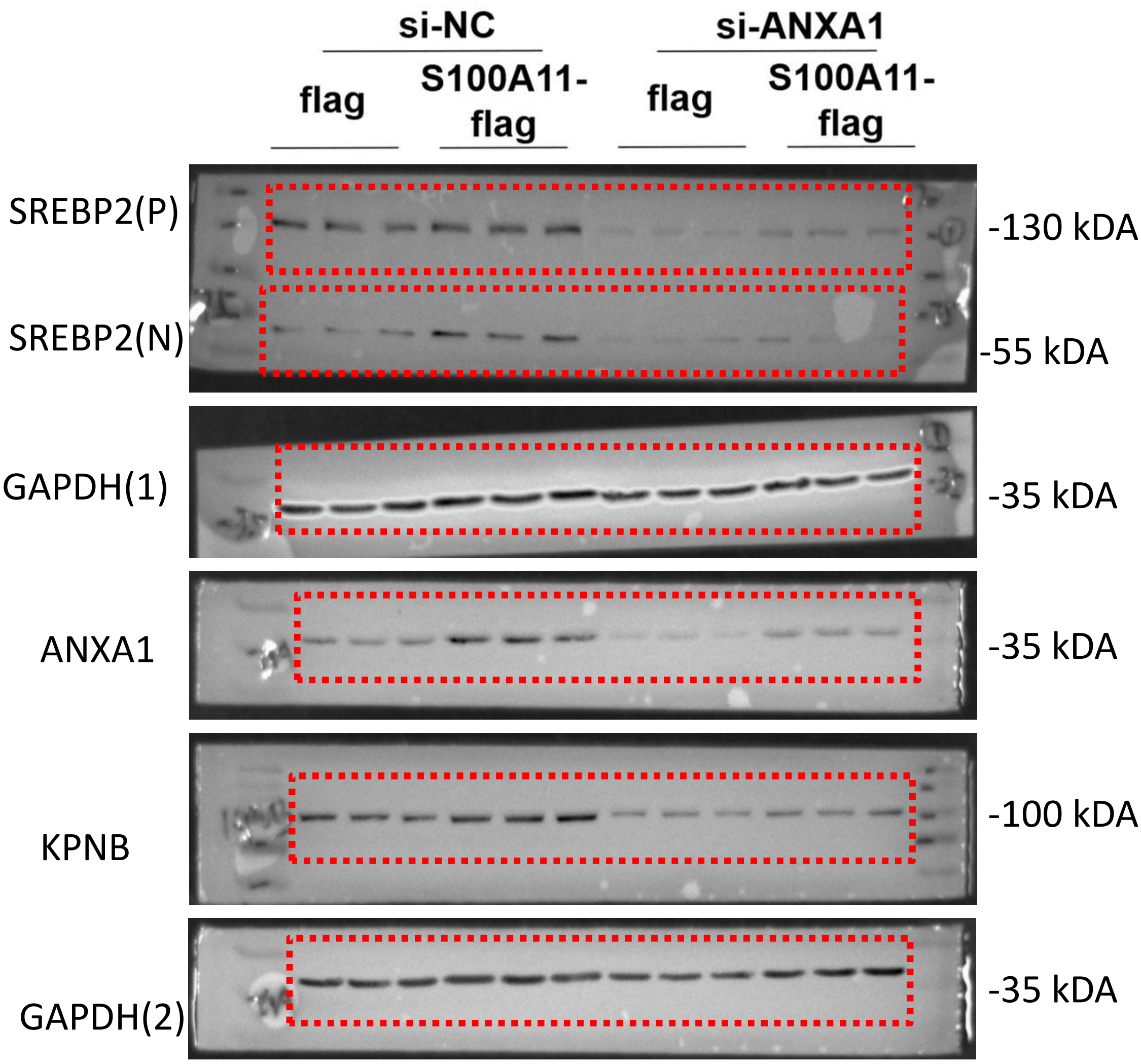

Full unedited blot/gel for Figure 7E

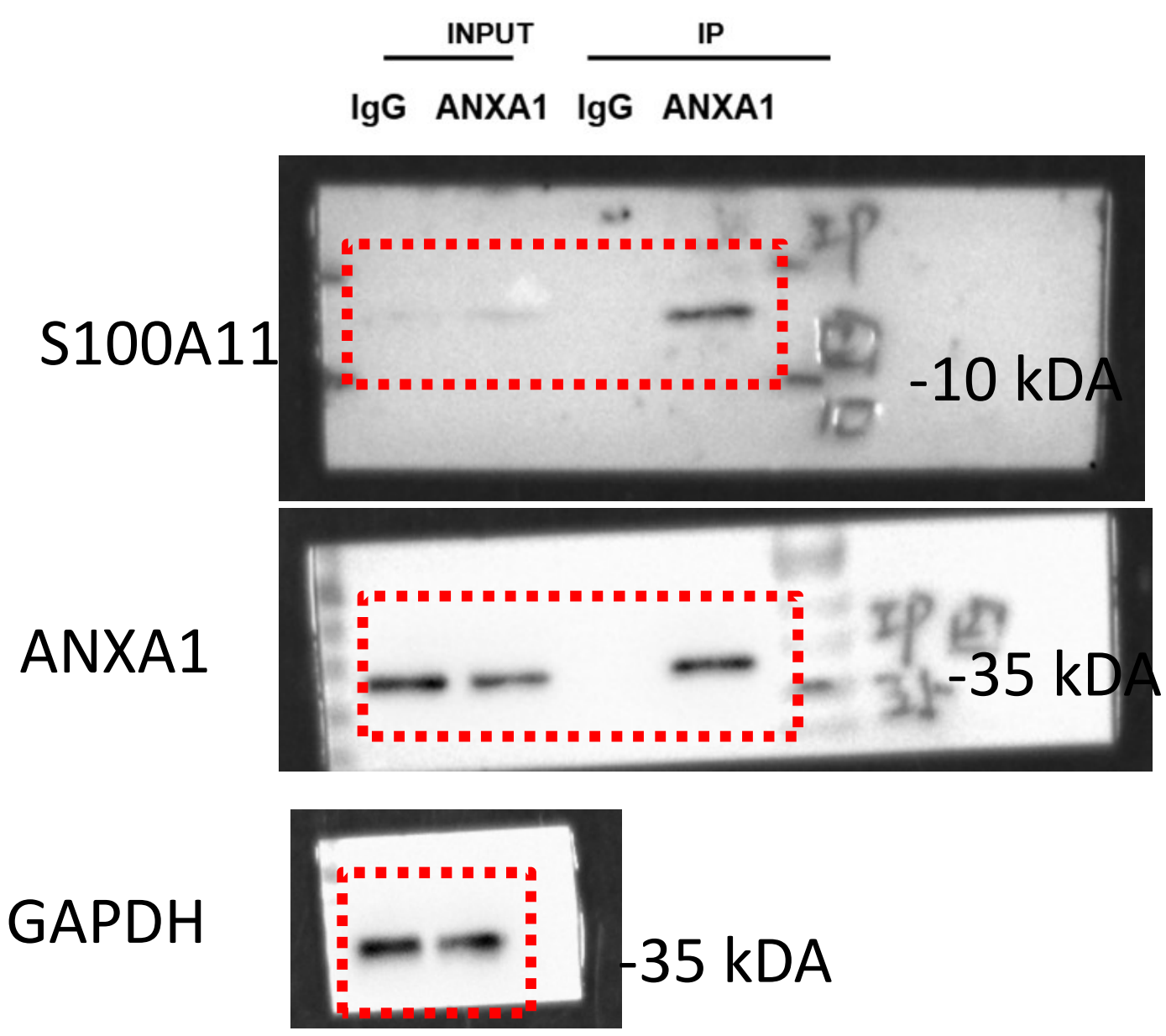

Full unedited blot/gel for Figure 7F

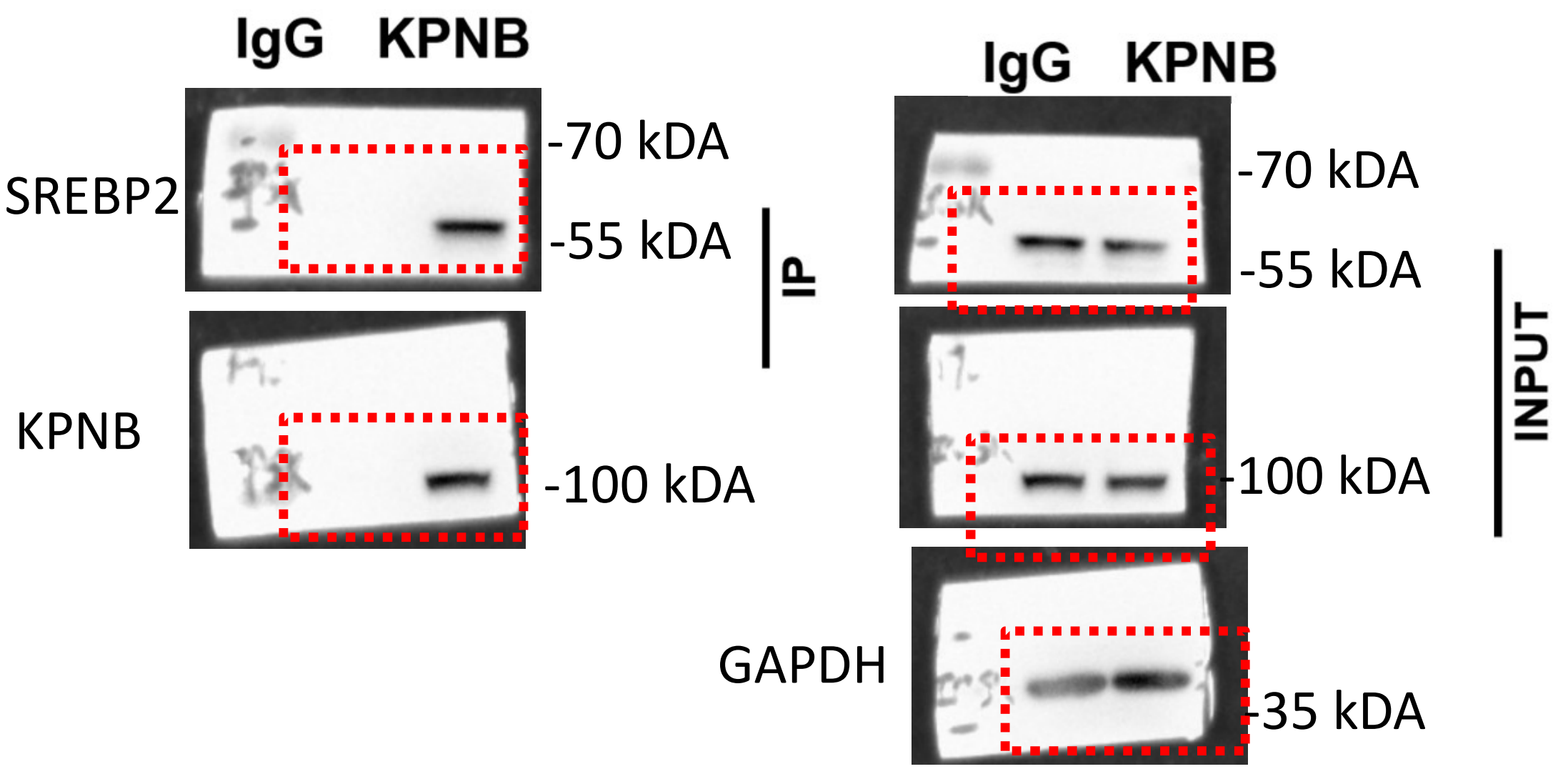

Full unedited blot/gel for Figure 7G

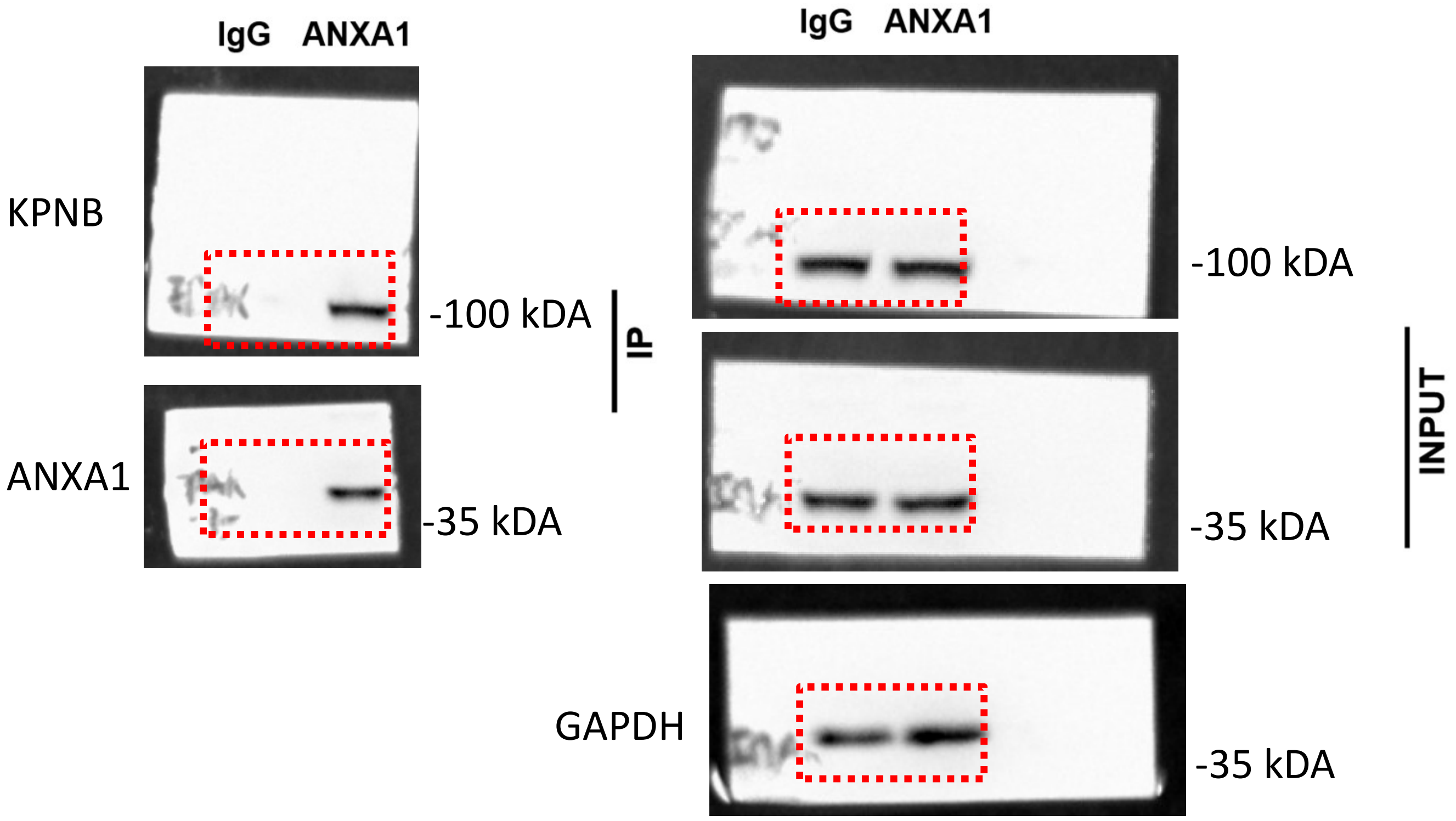

Full unedited blot/gel for Figure 7H

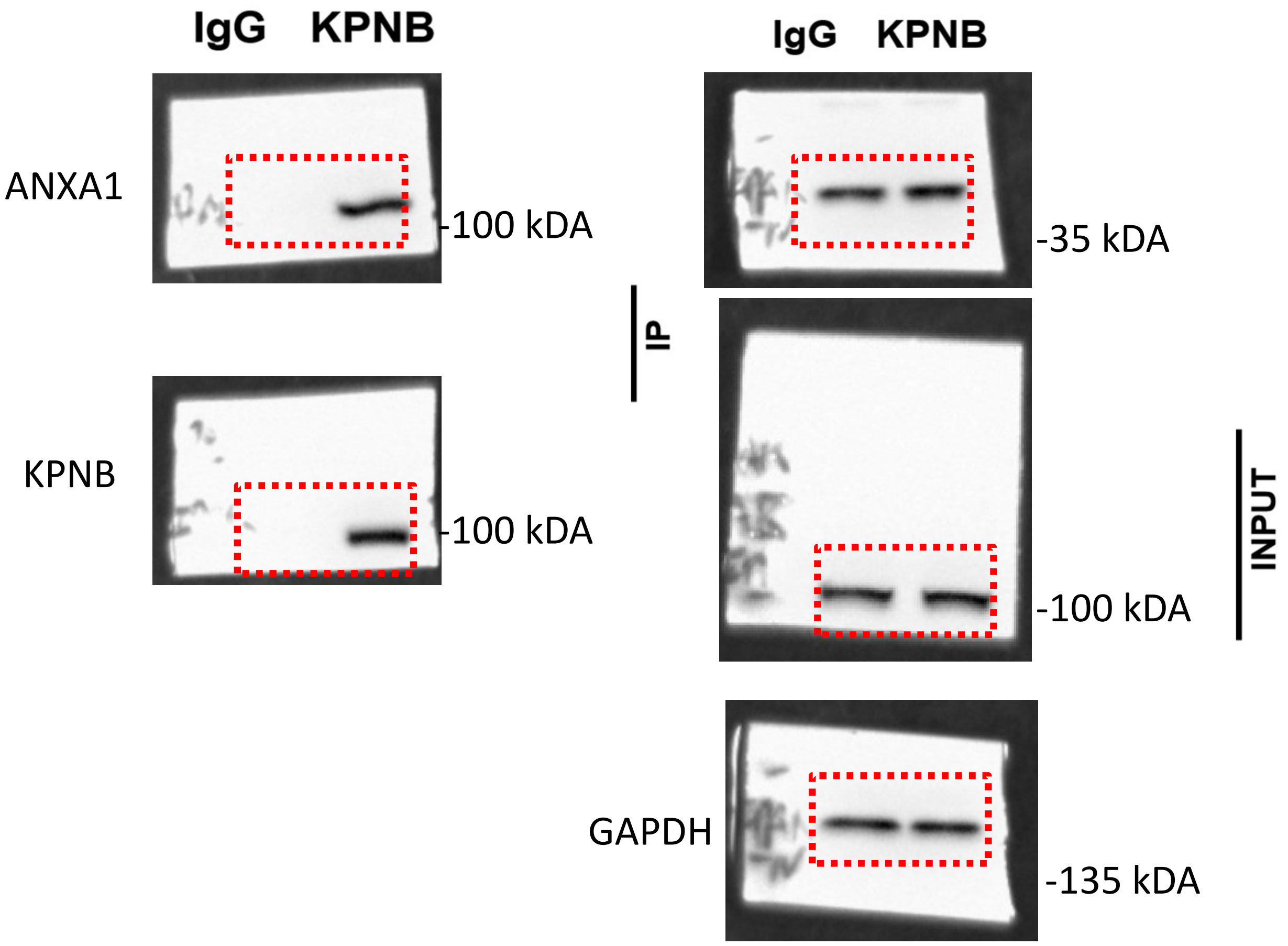

Full unedited blot/gel for Figure 7J

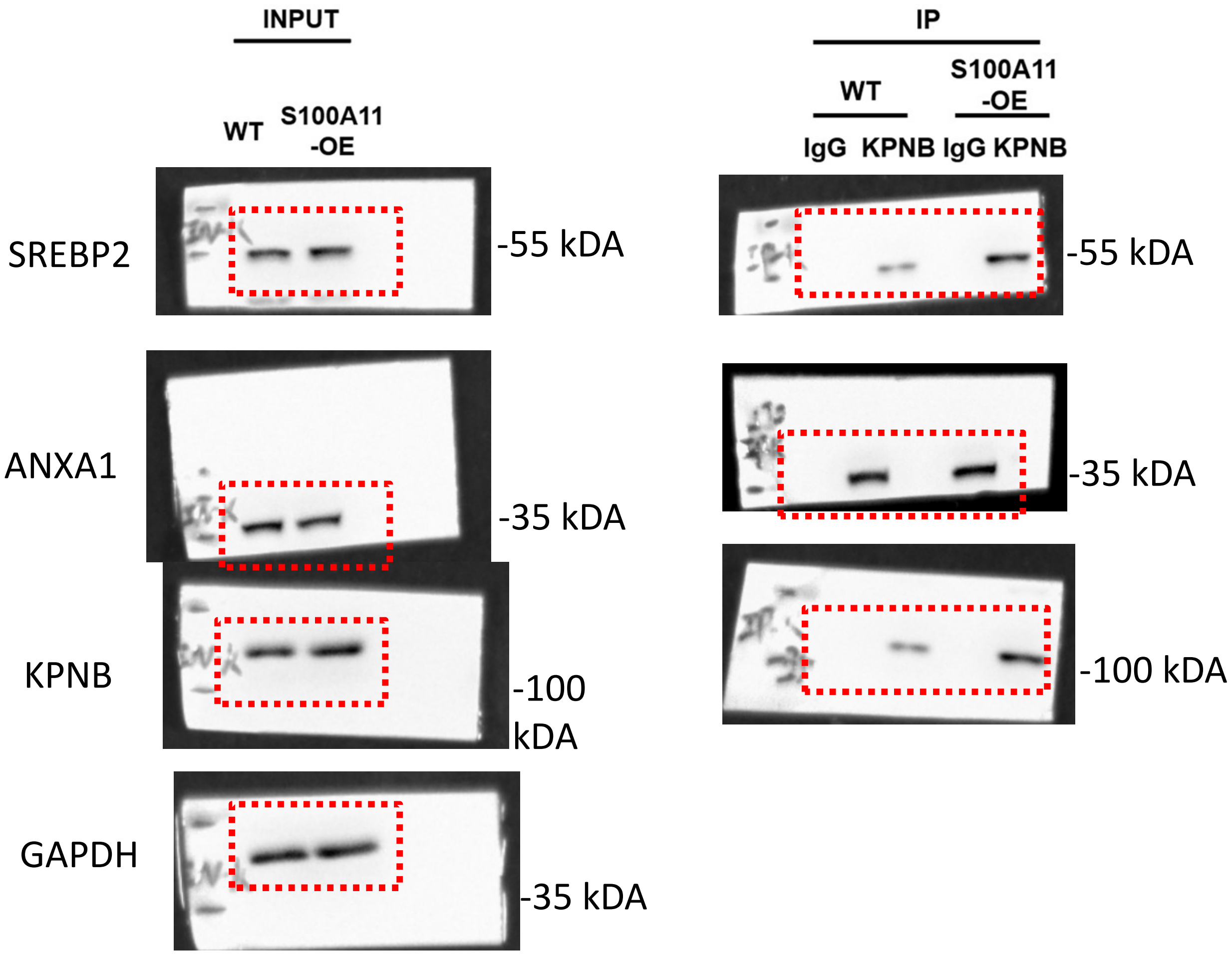

Full unedited blot/gel for Figure 7K

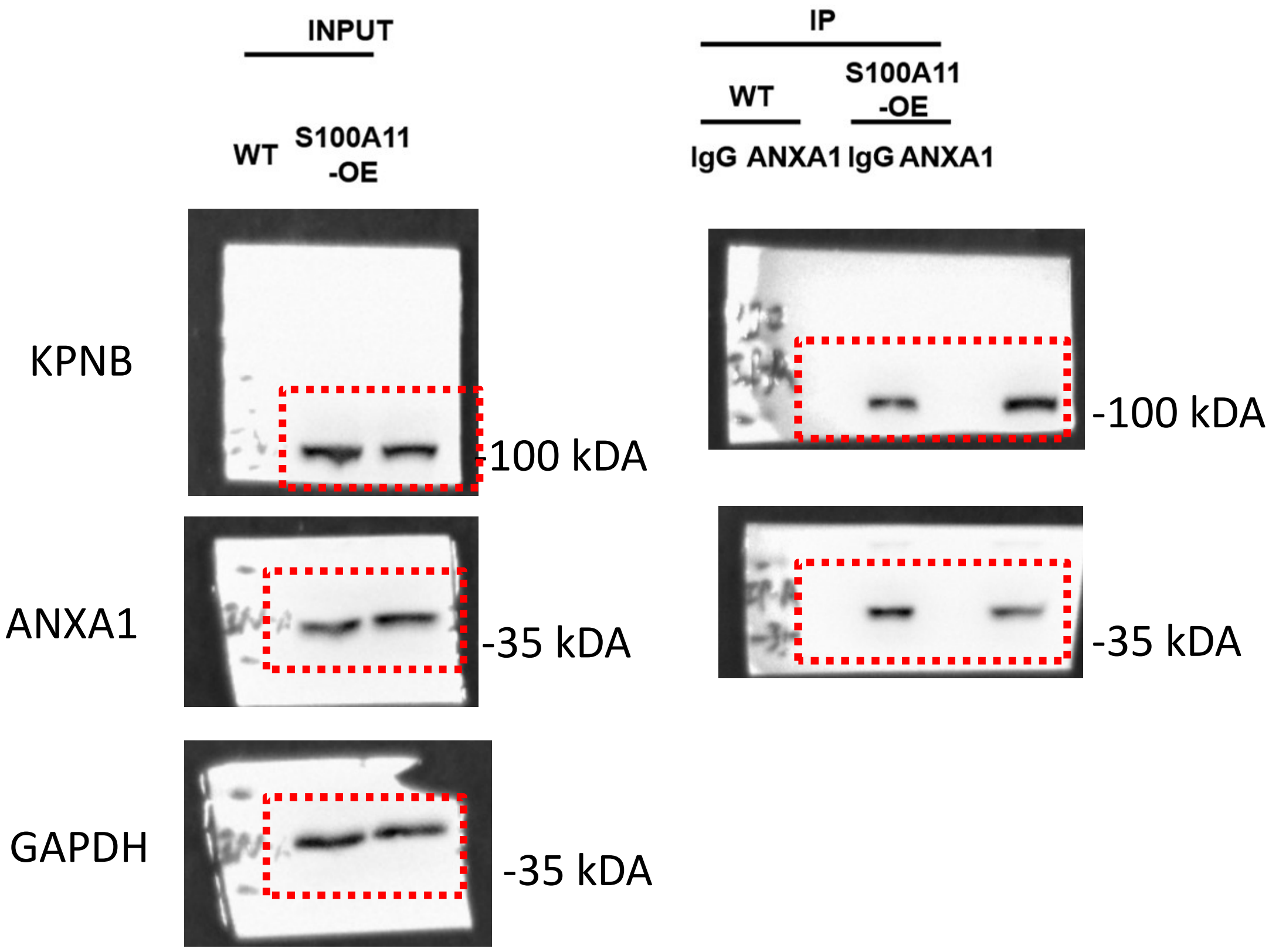

Full unedited blot/gel for Figure 7M

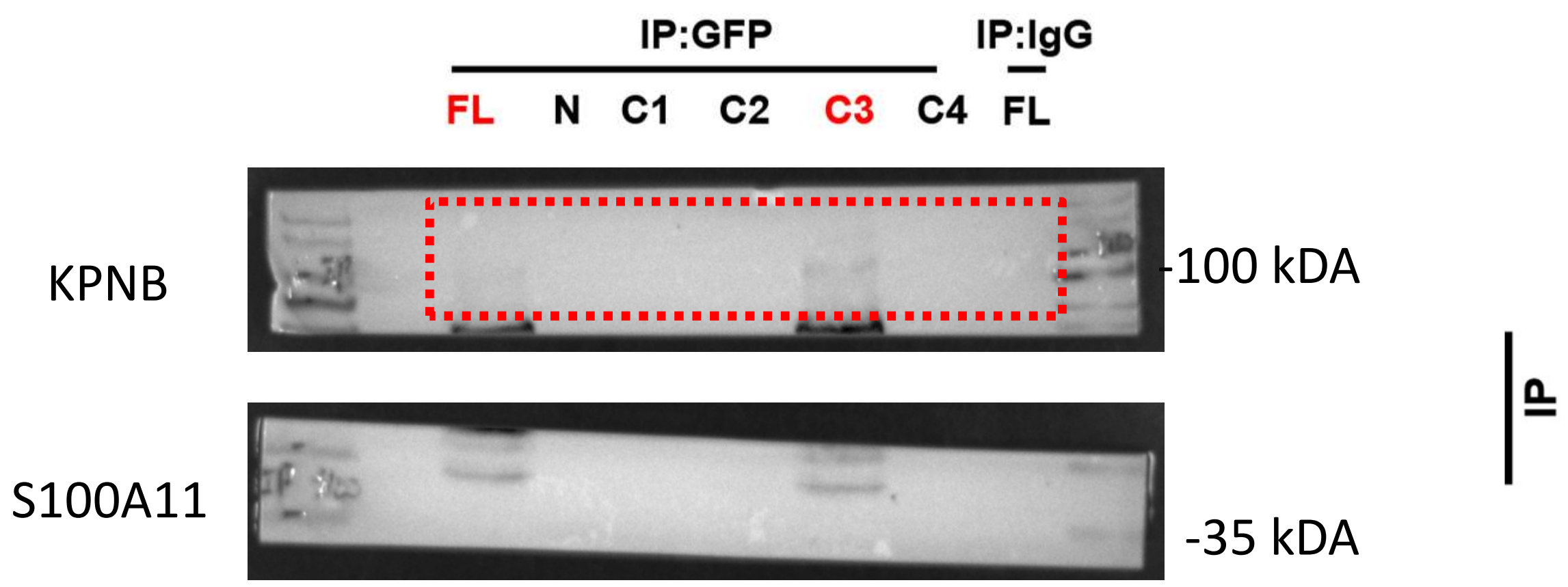

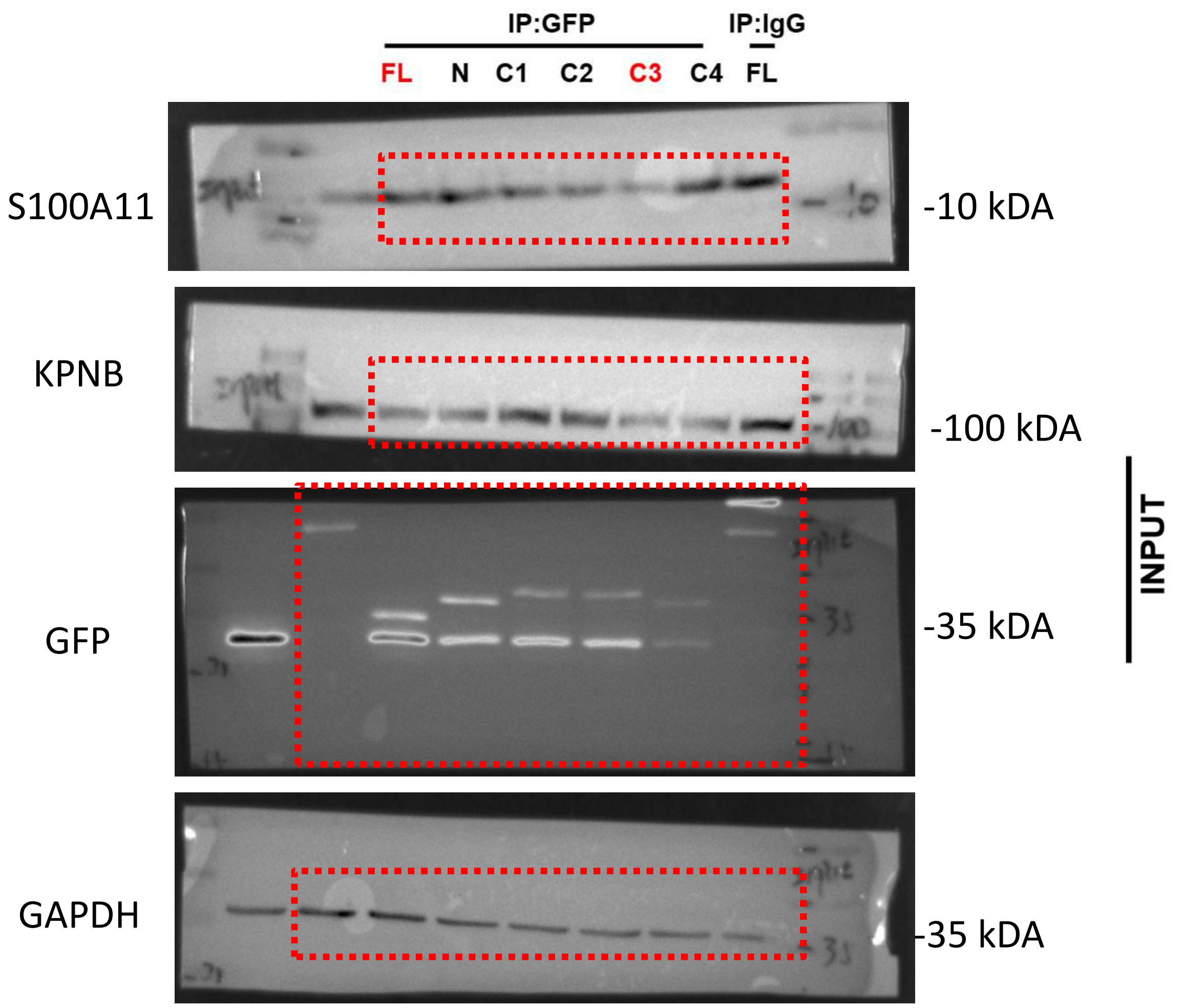

Full unedited blot/gel for Figure 8A

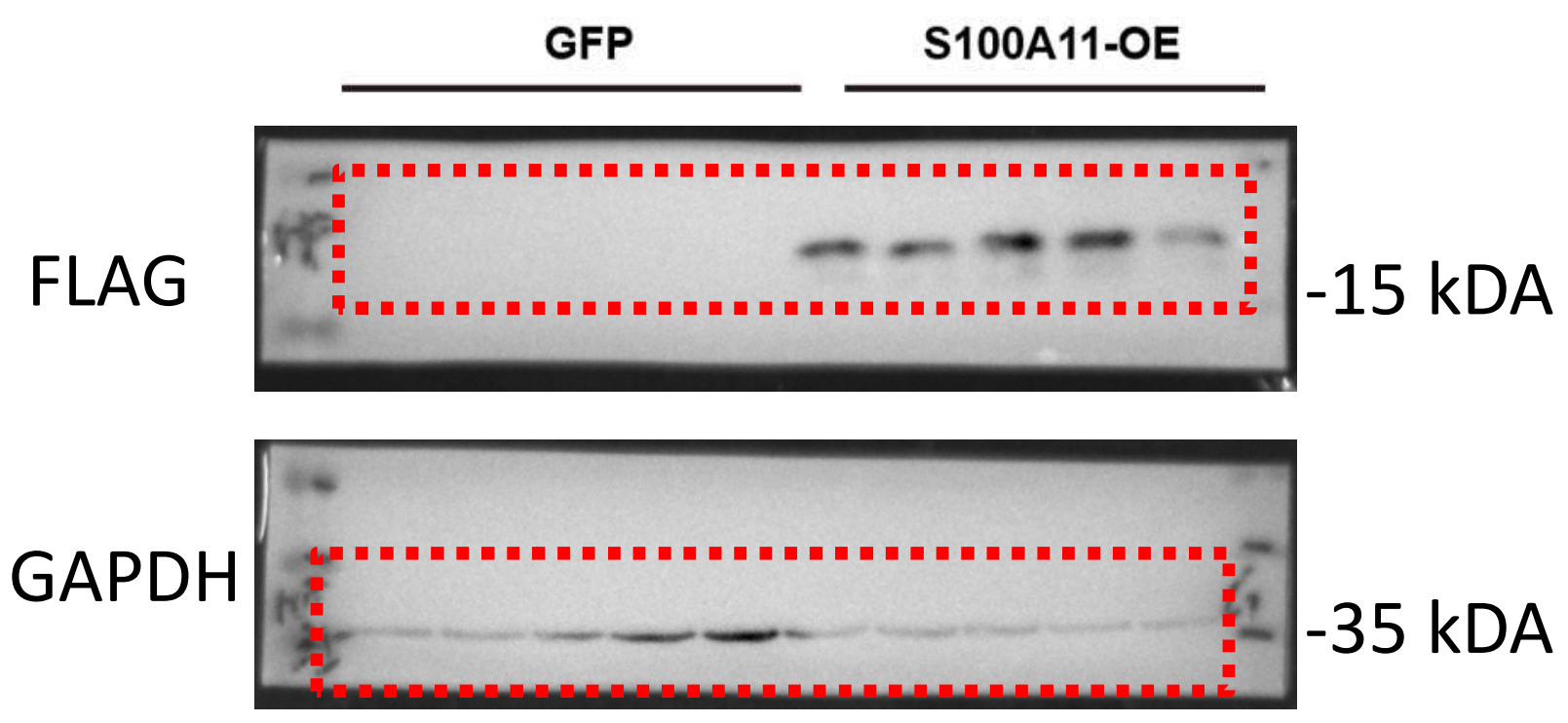

Full unedited blot/gel for Figure 8D

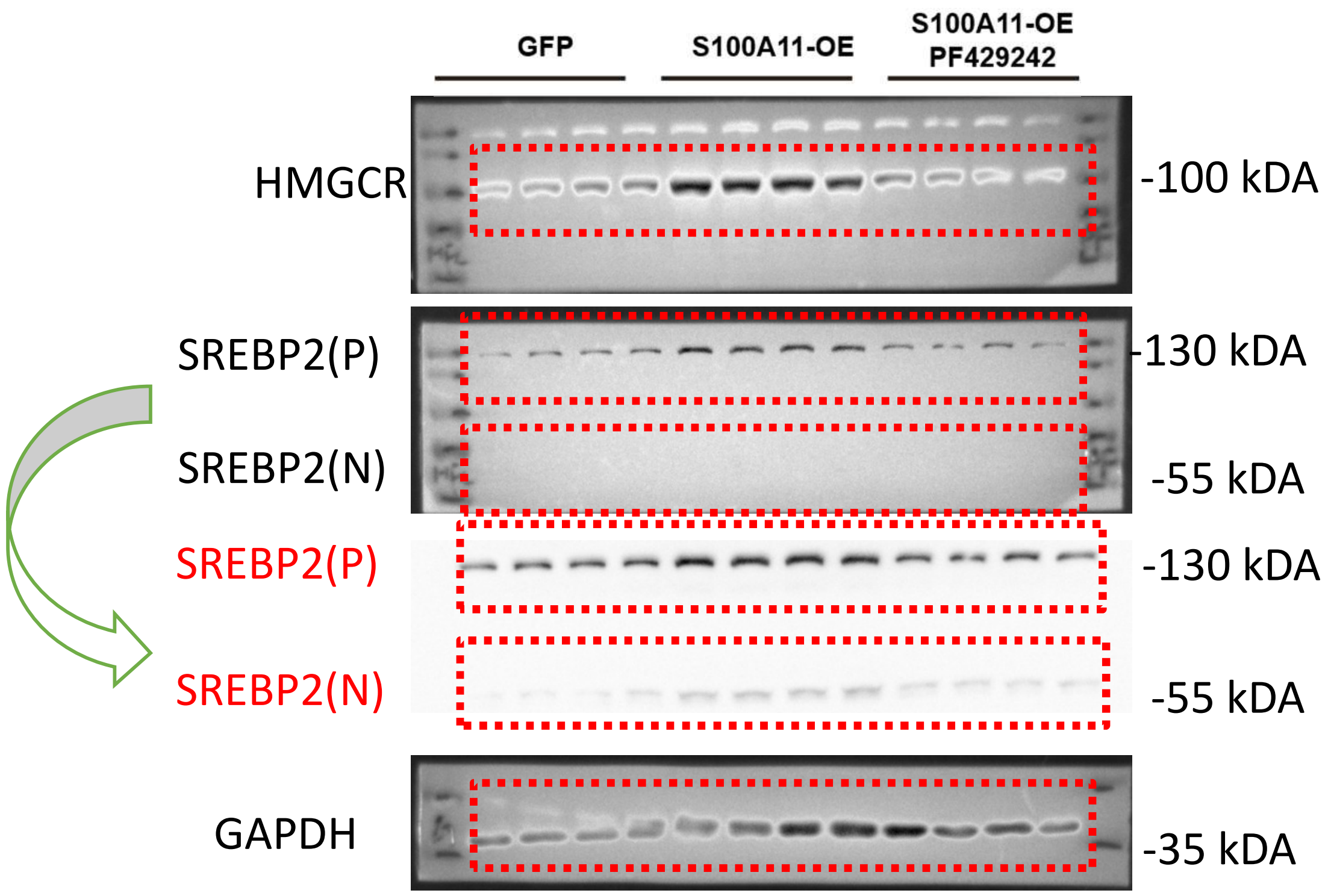

Full unedited blot/gel for Figure 8E

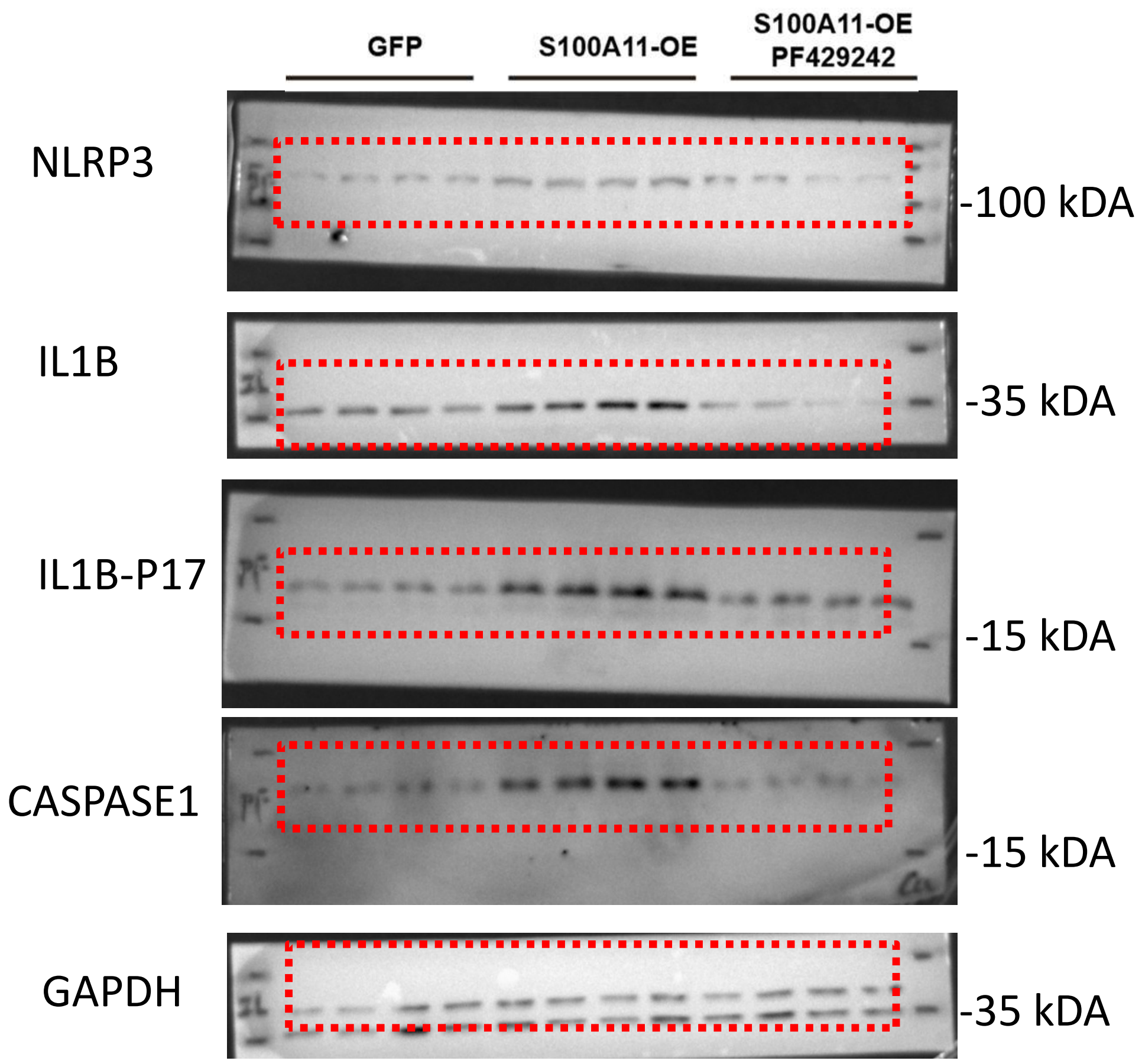

Full unedited blot/gel for Figure 8F

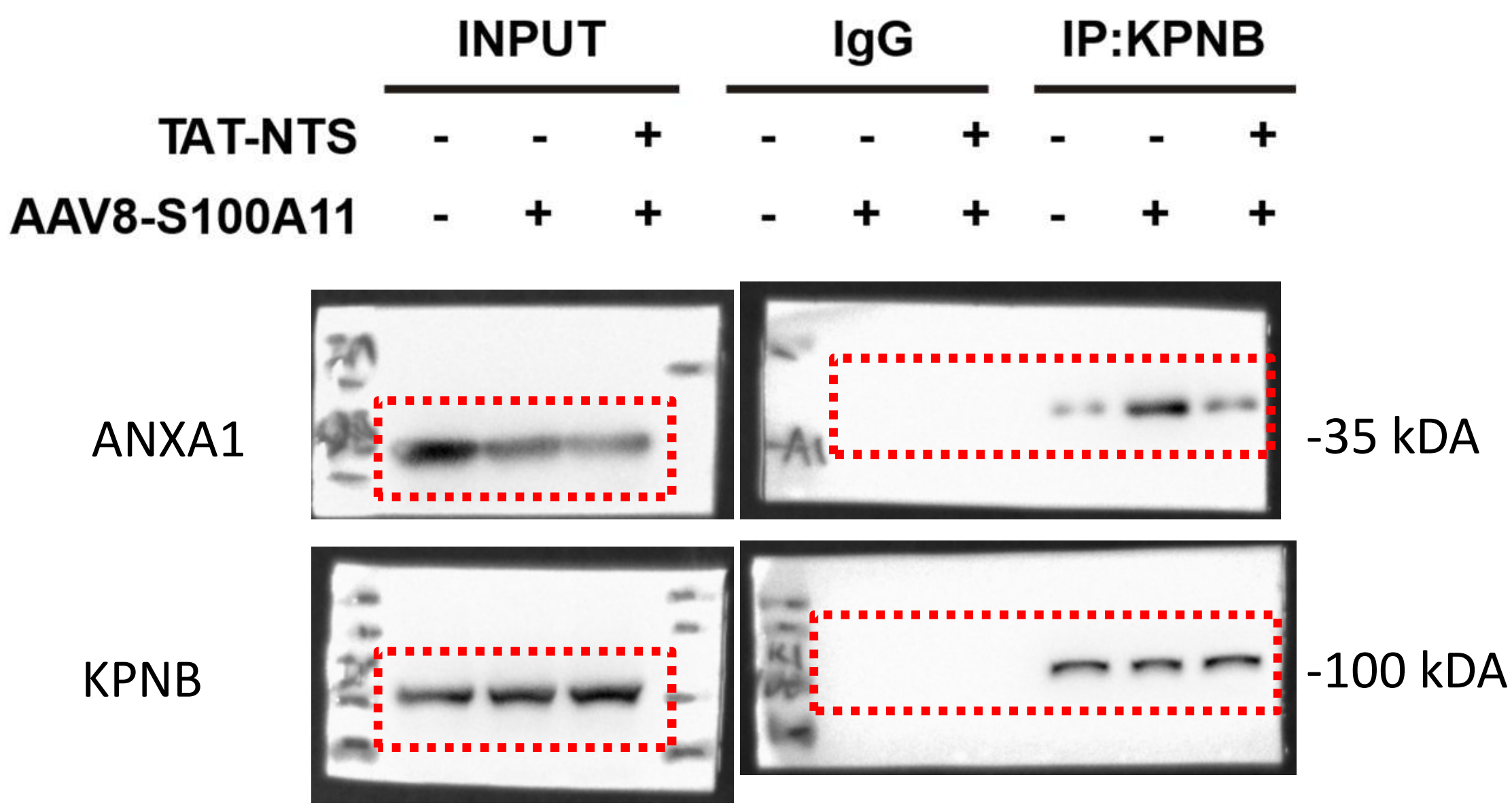

Full unedited blot/gel for Figure 8F

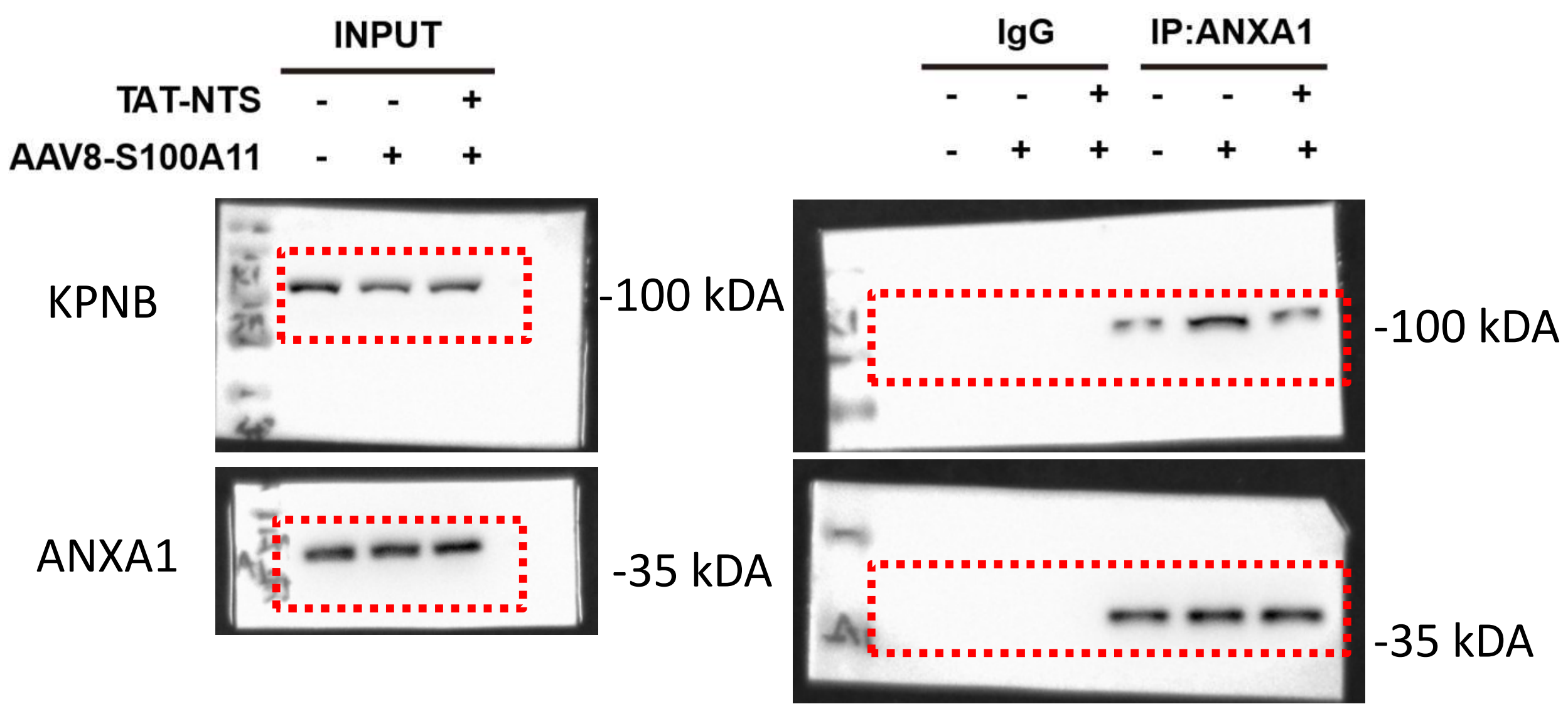

Full unedited blot/gel for Figure 8I

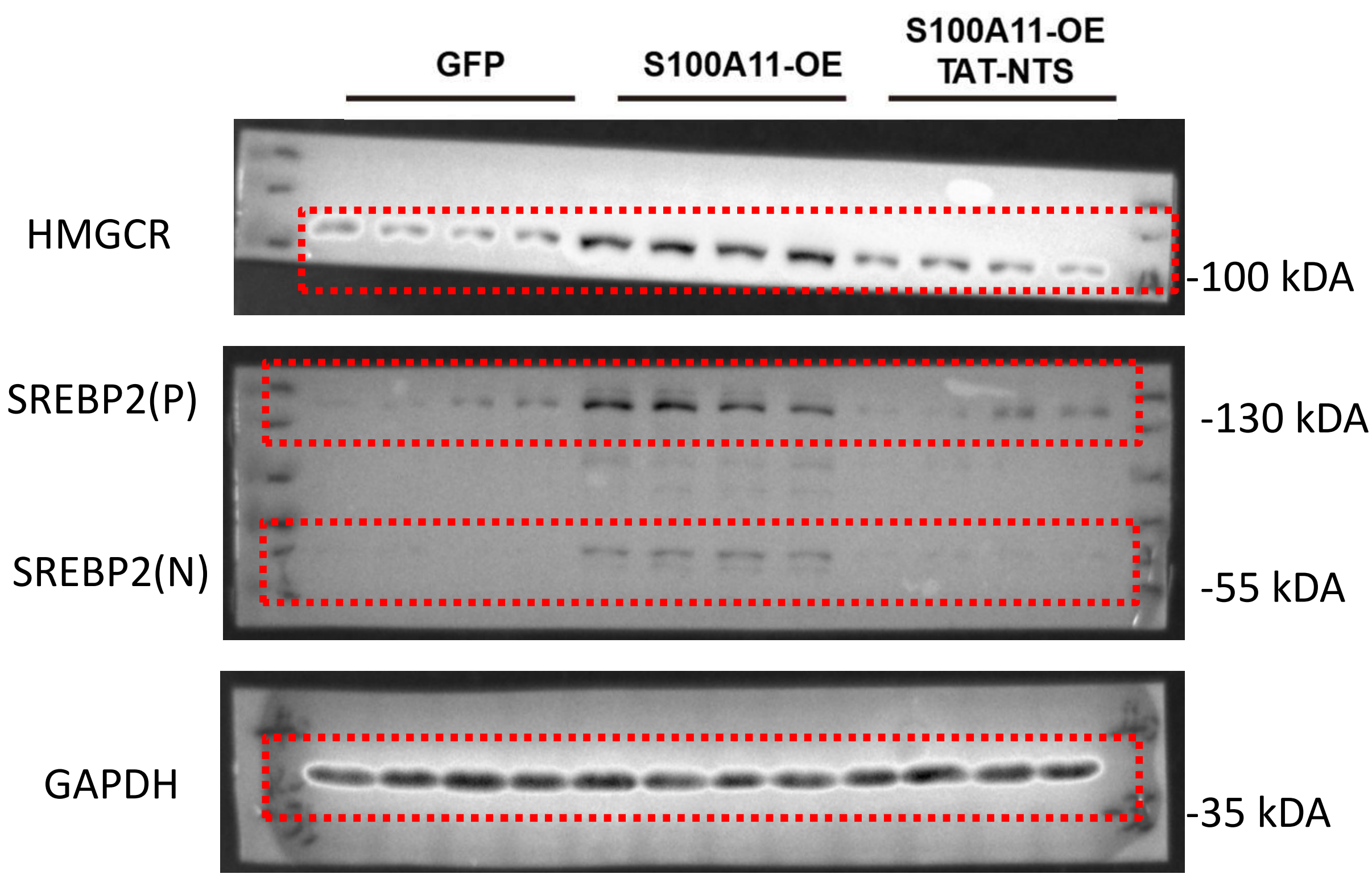

Full unedited blot/gel for Figure 8J

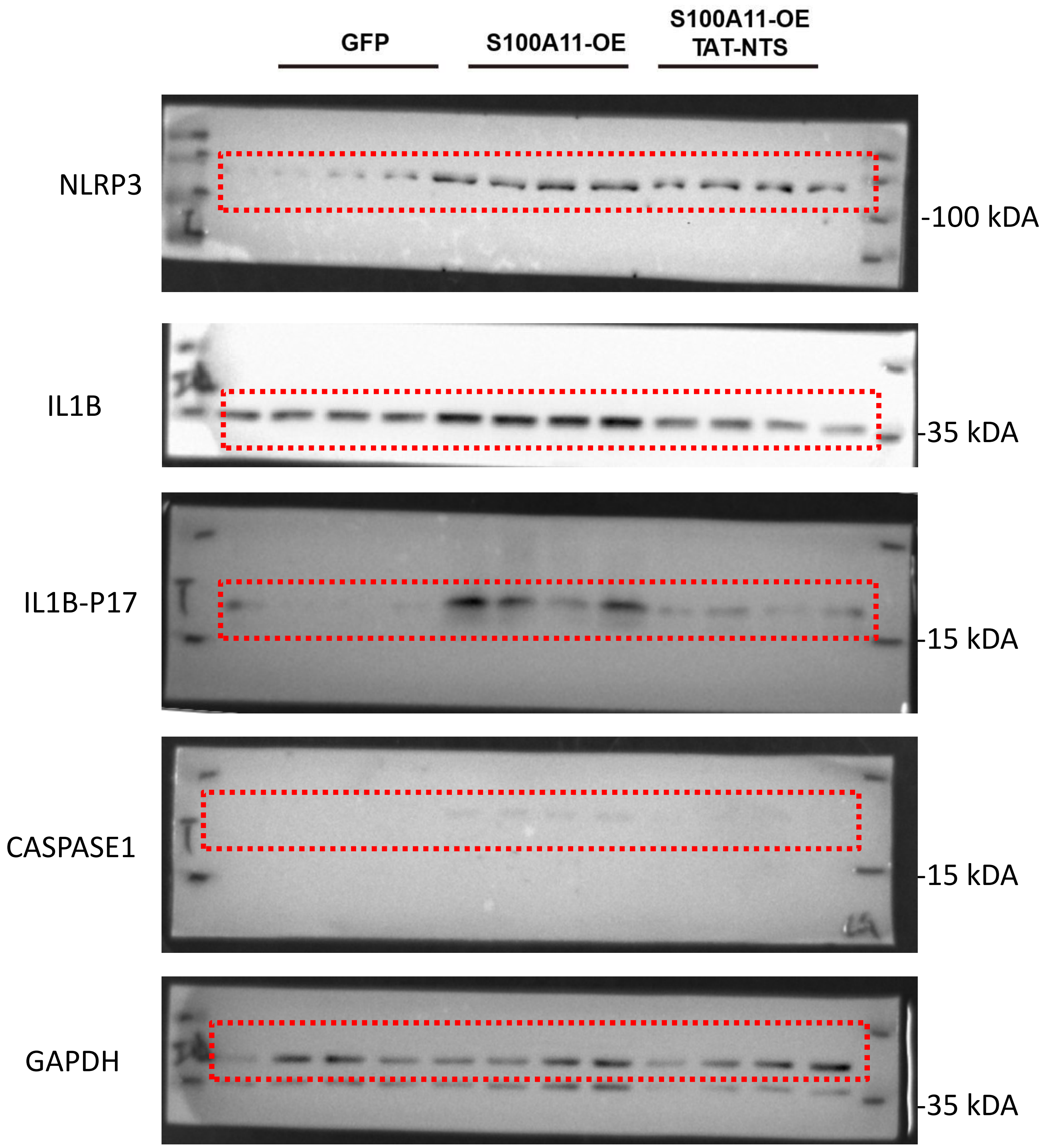

Full unedited blot/gel for Figure S1A

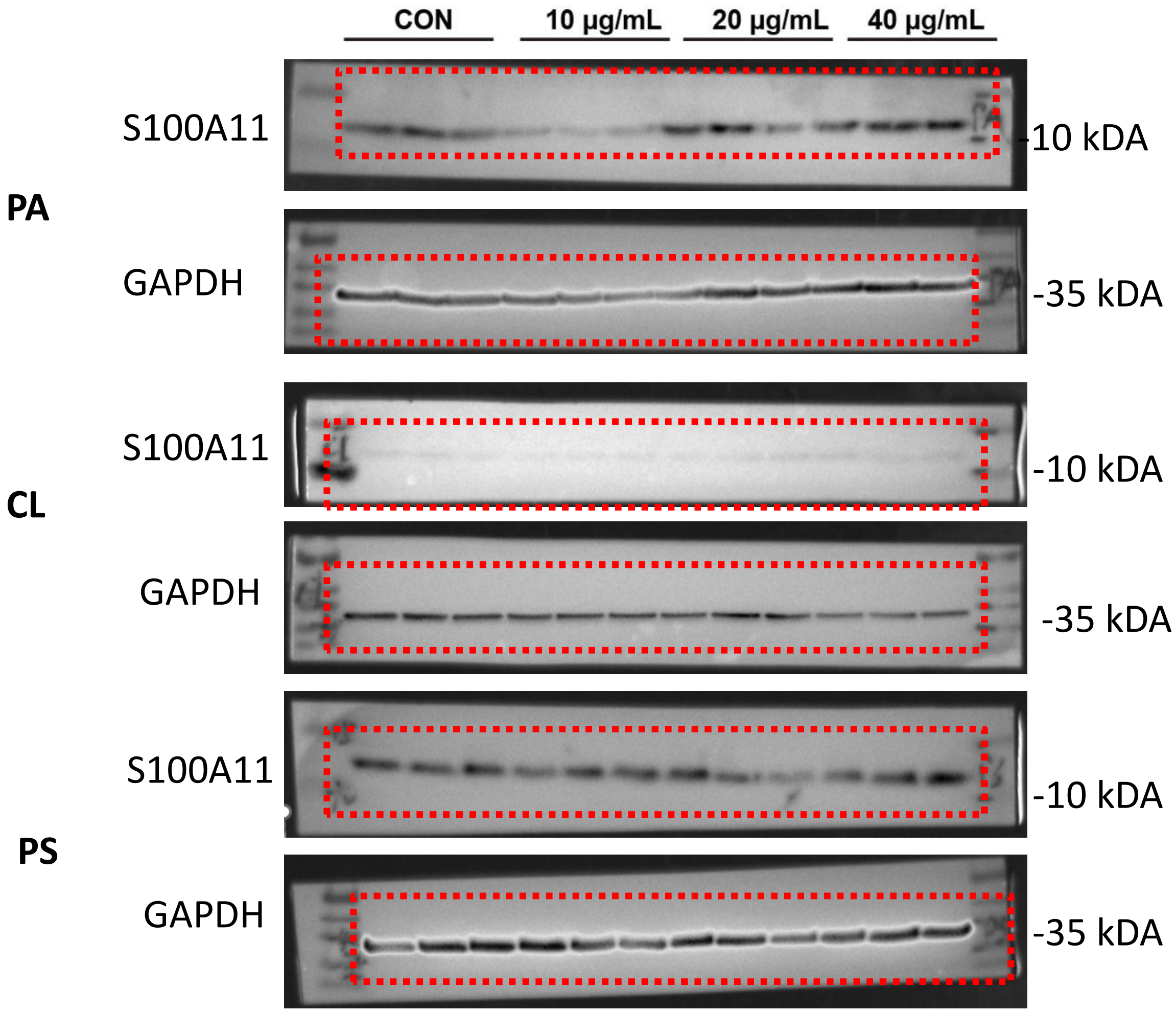

Full unedited blot/gel for Figure S1C

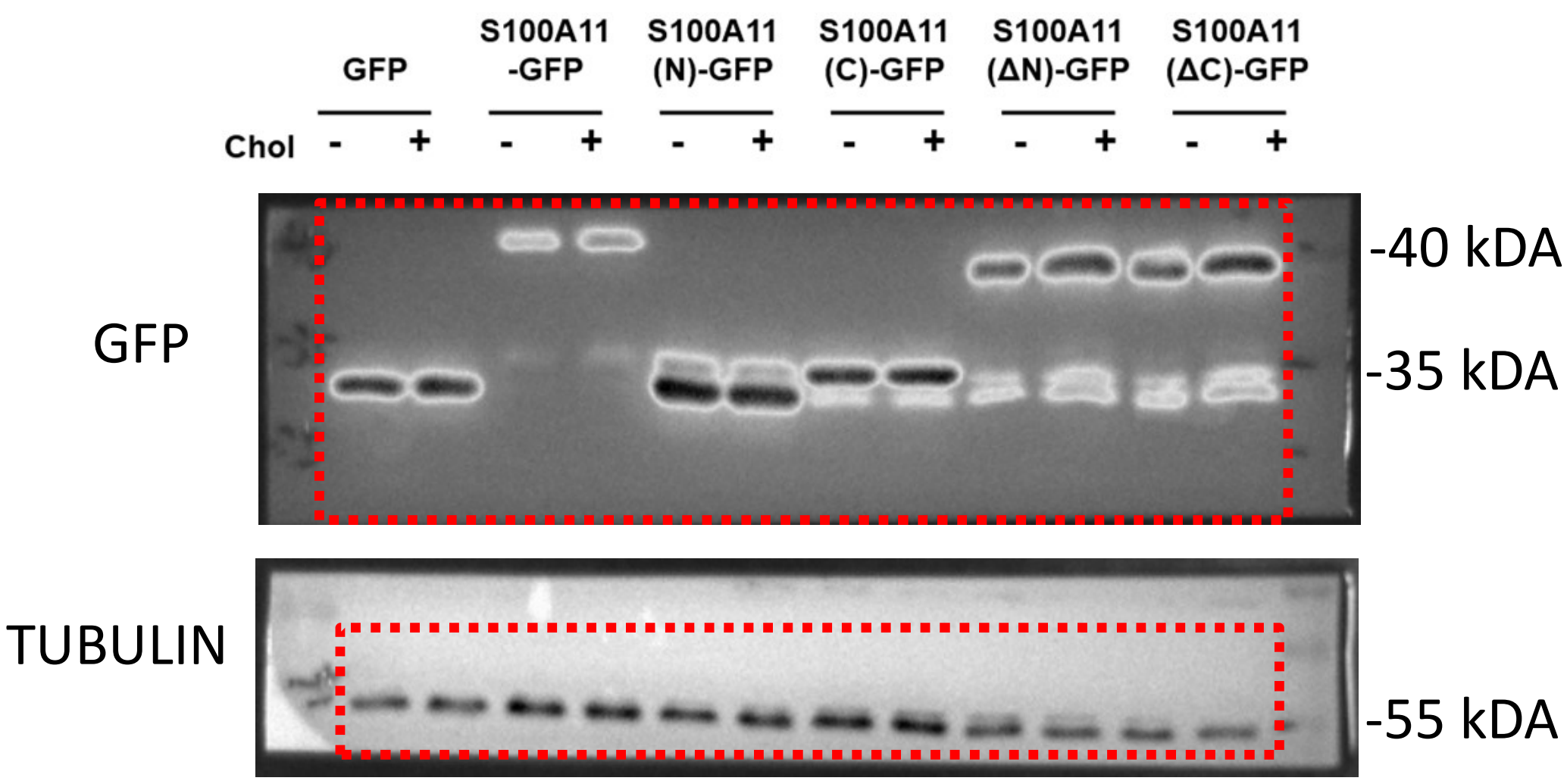

Full unedited blot/gel for Figure S2C

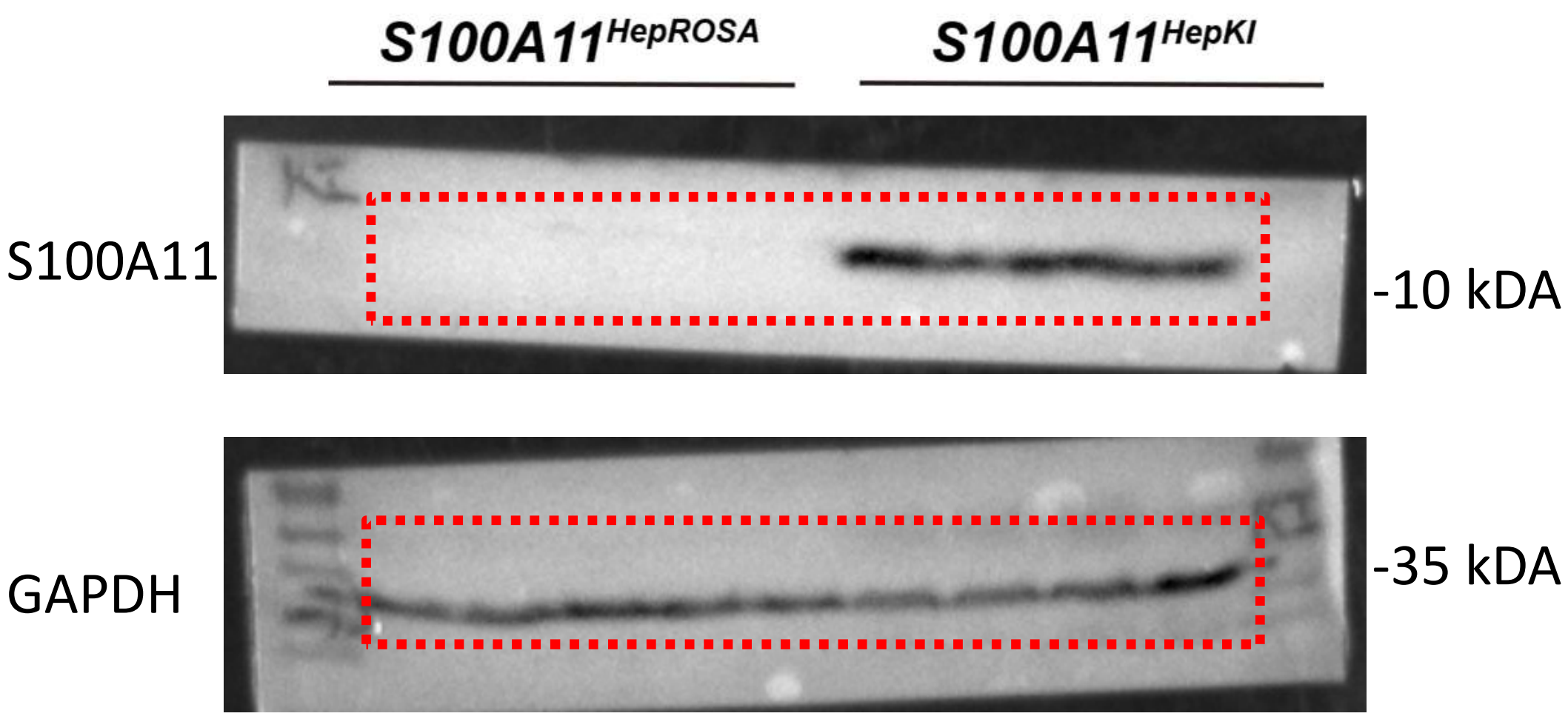

Full unedited blot/gel for Figure S3C

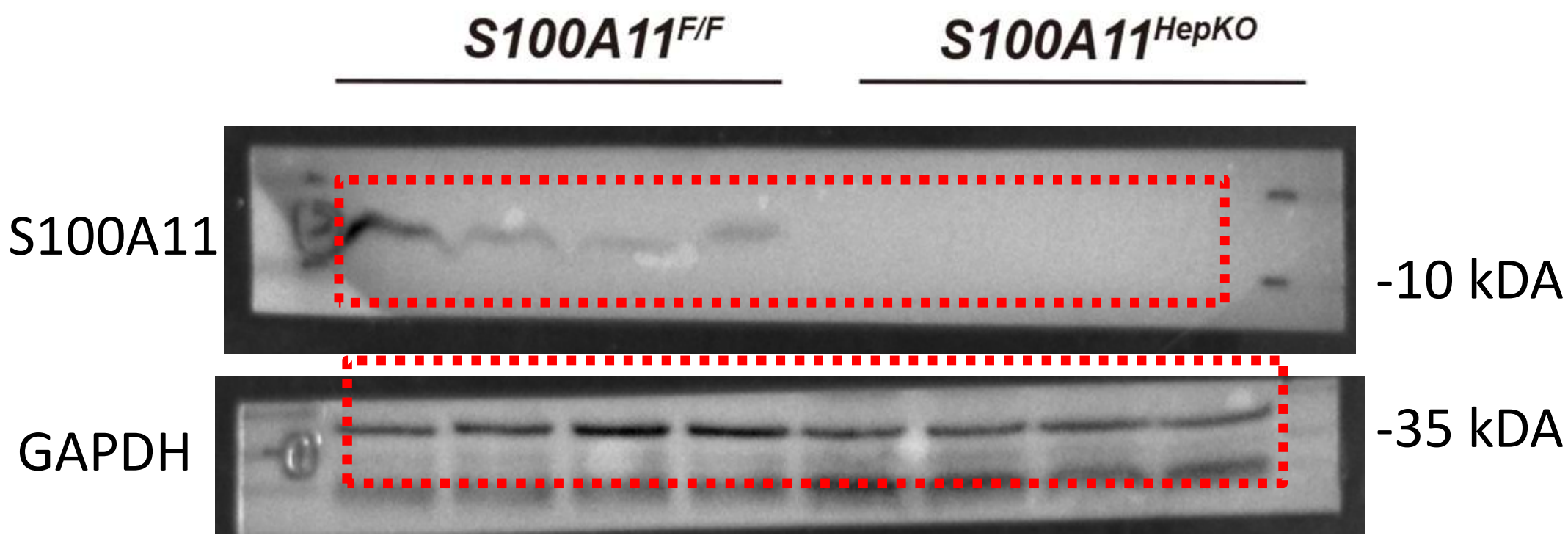

Full unedited blot/gel for Figure S4A

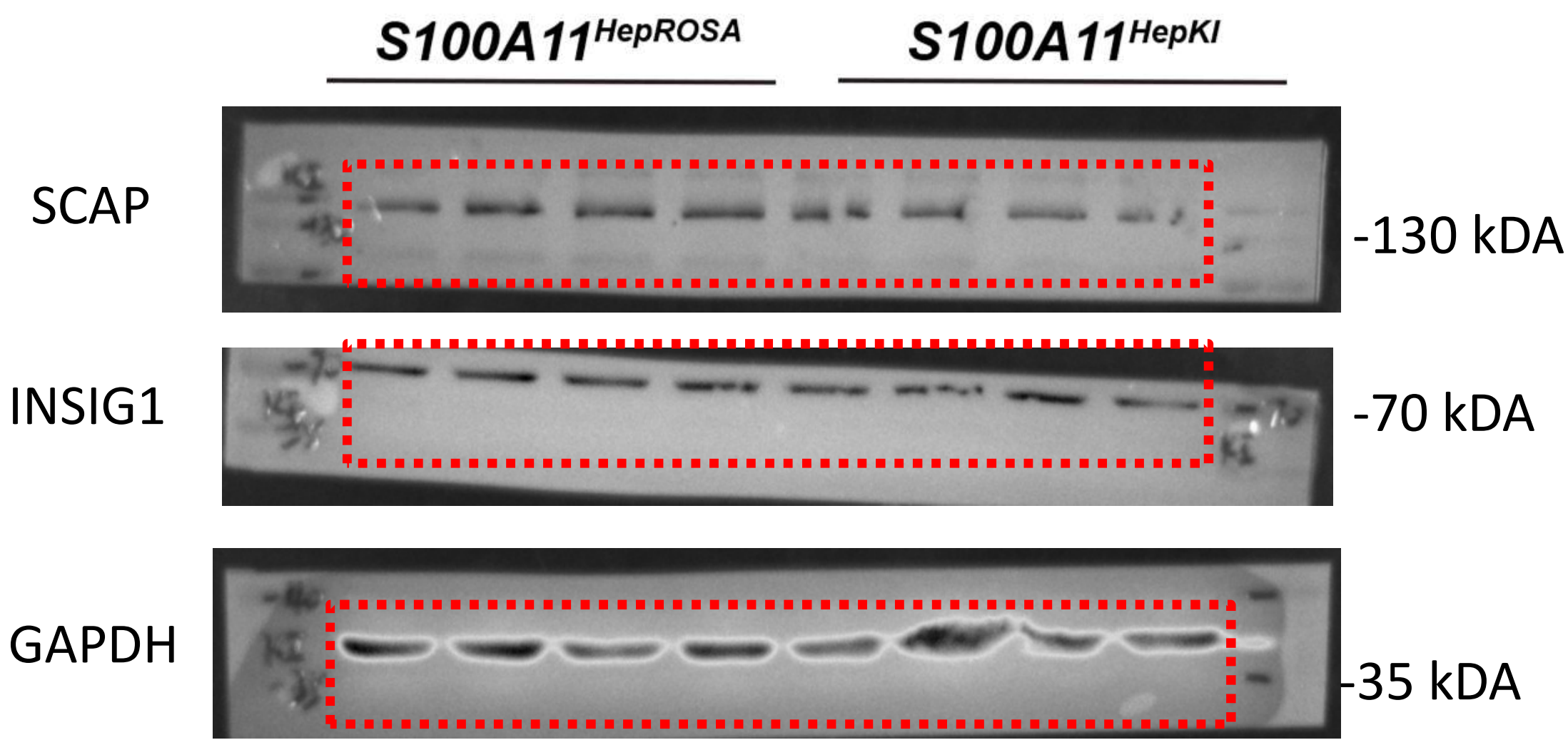

Full unedited blot/gel for Figure S4B

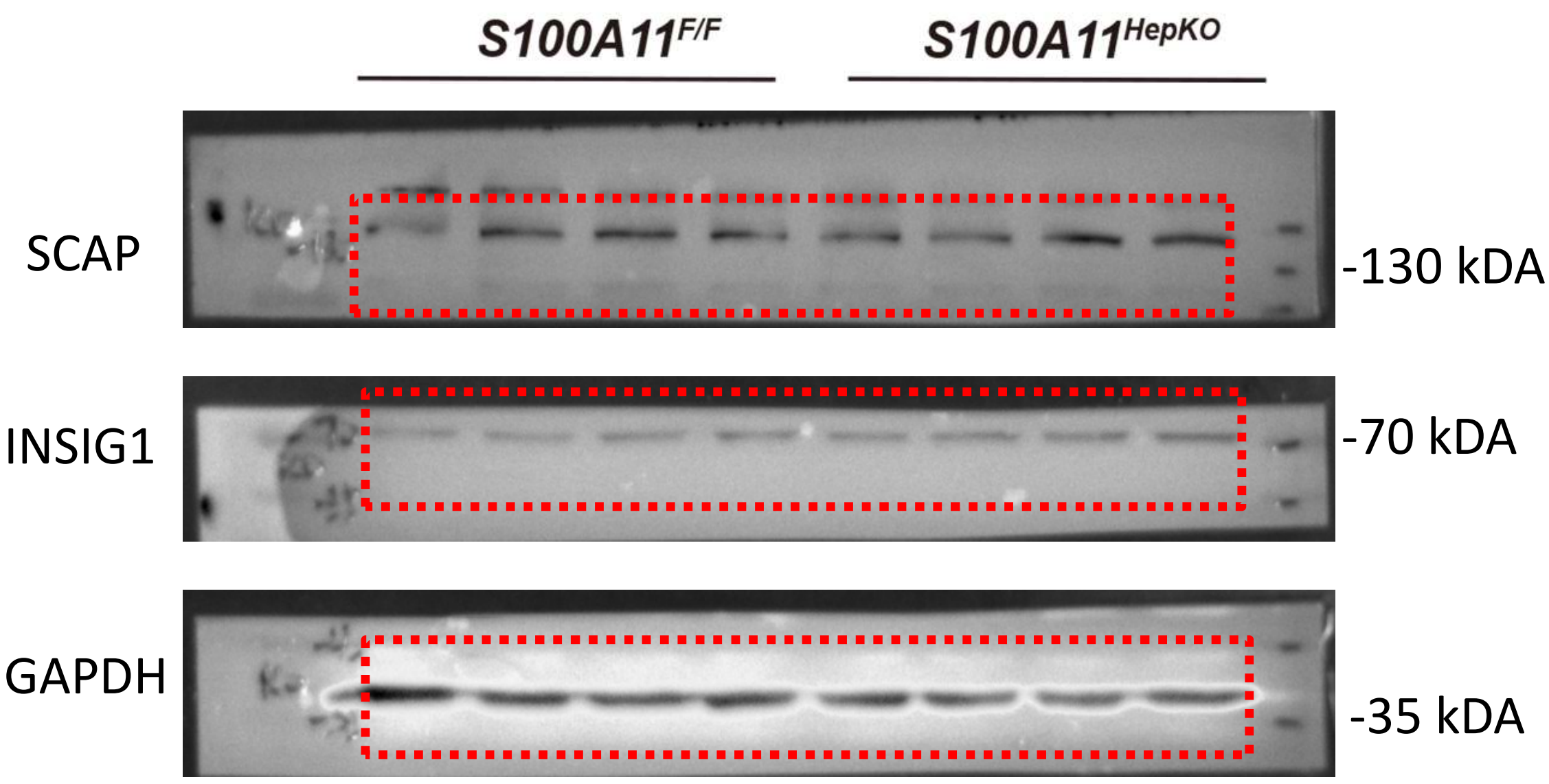

Full unedited blot/gel for Figure S4C and D

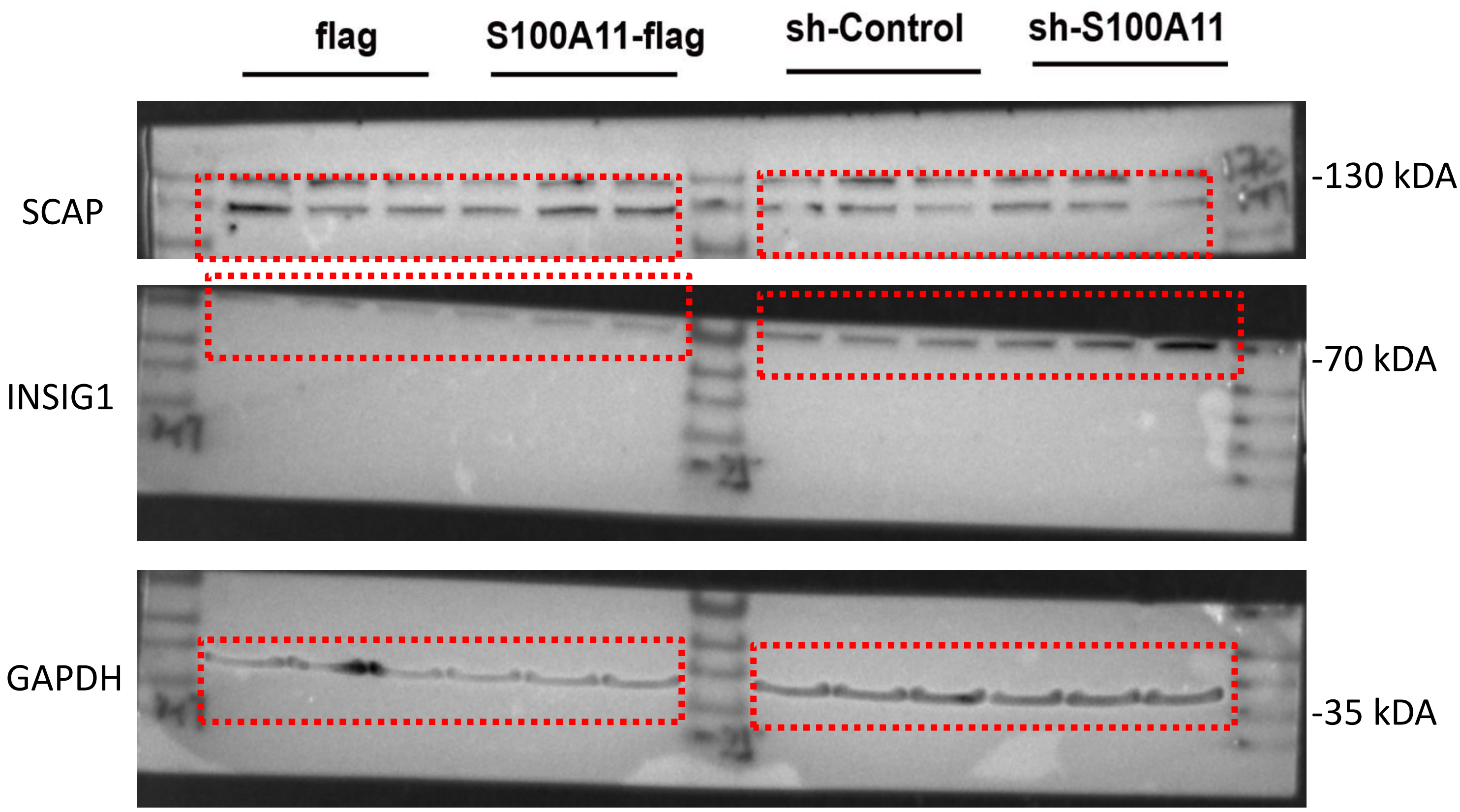

Full unedited blot/gel for Figure S4F

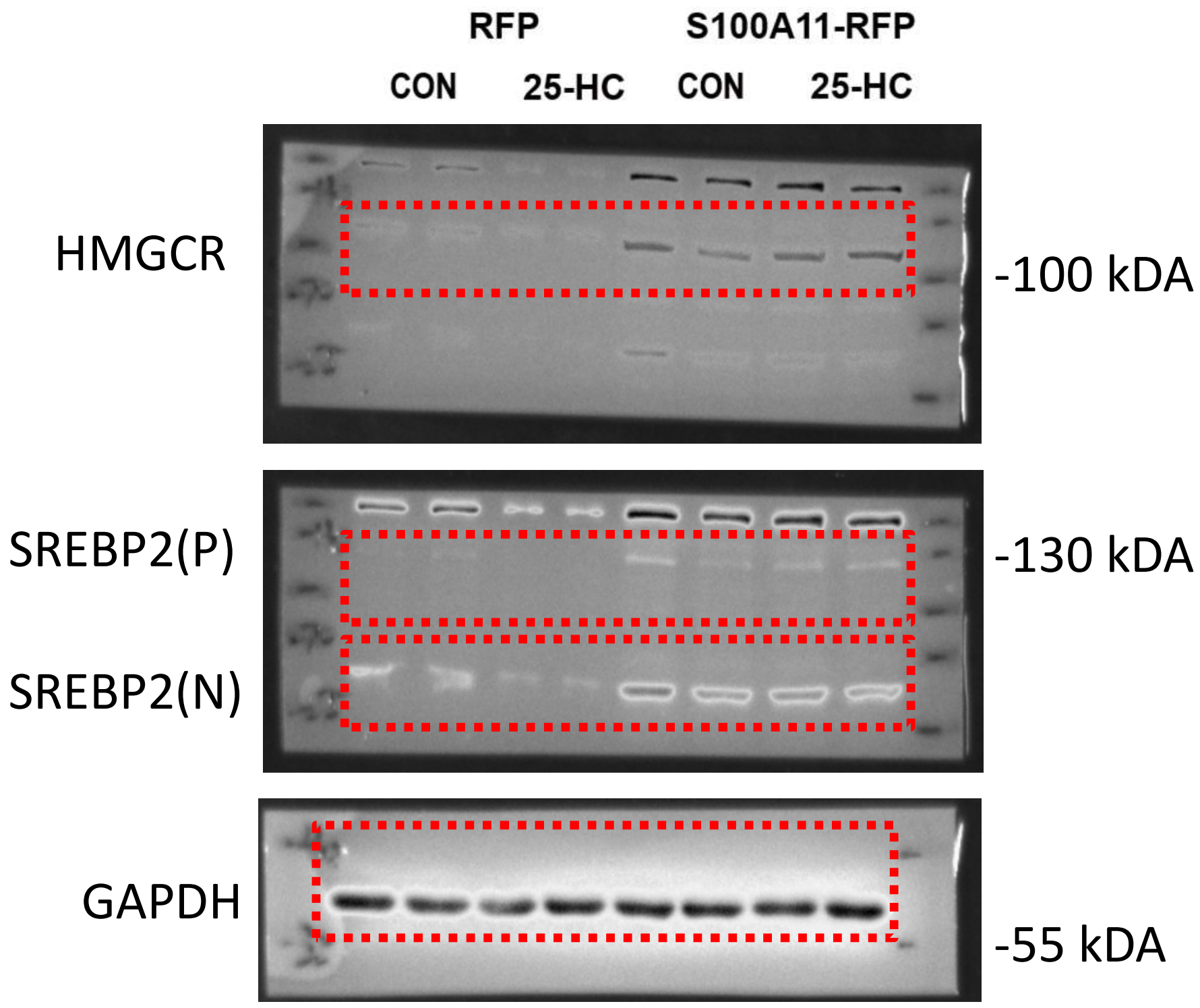

Full unedited blot/gel for Figure S4H

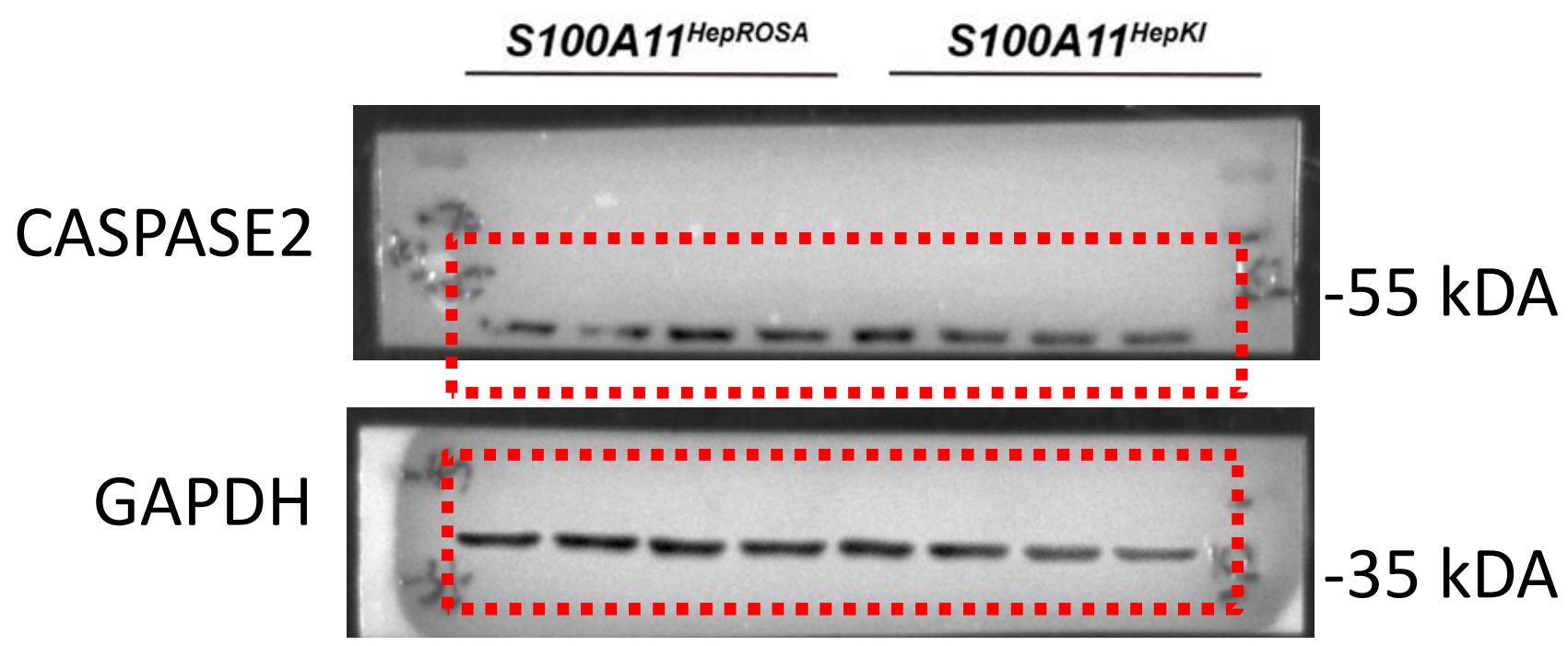

Full unedited blot/gel for Figure S4I

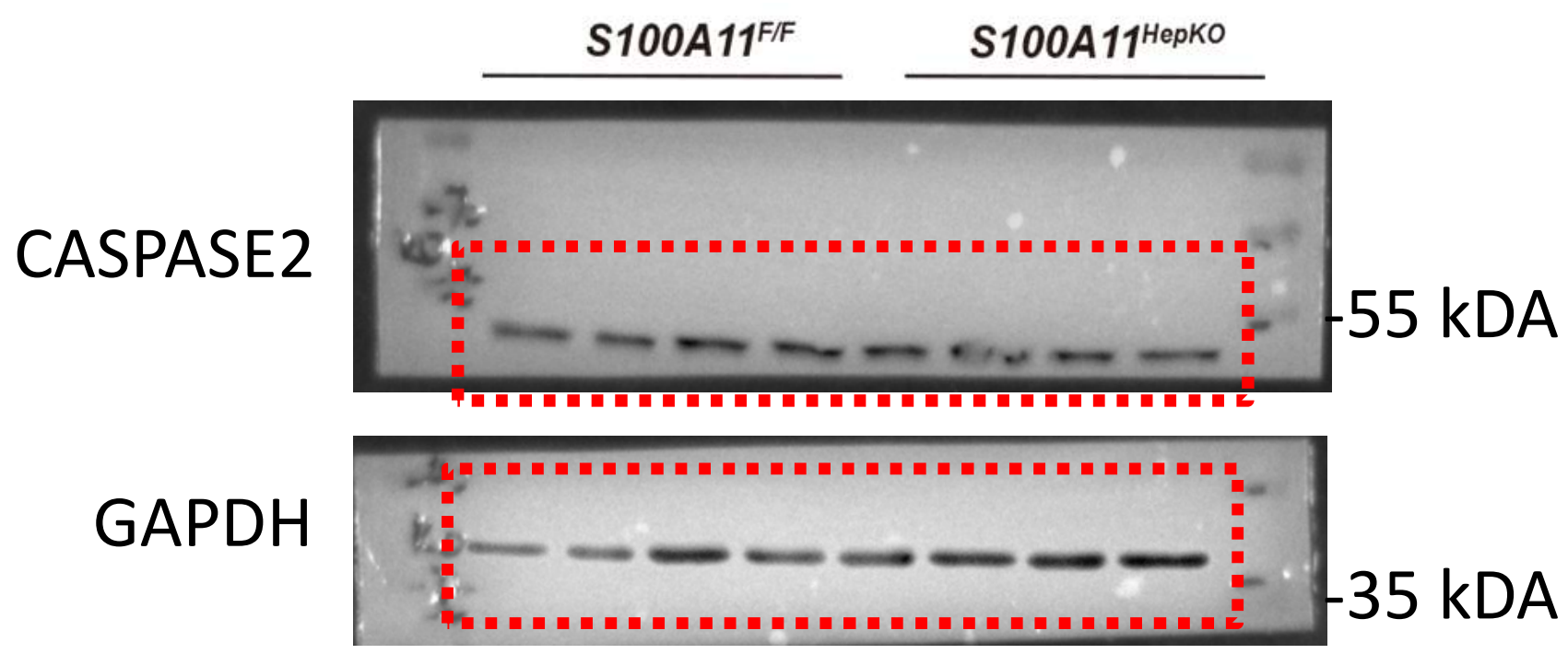

Full unedited blot/gel for Figure S4J

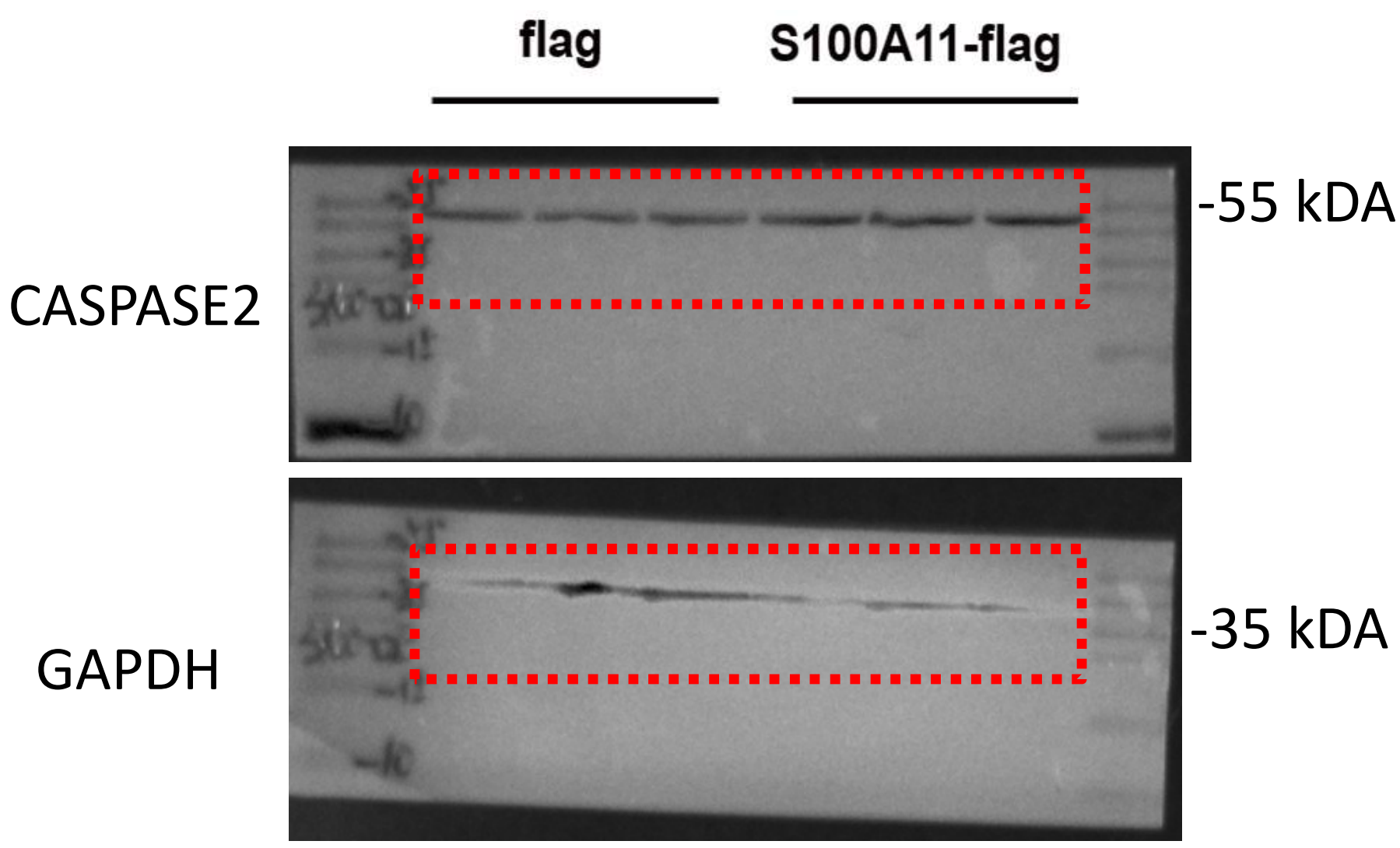

Full unedited blot/gel for Figure S4K

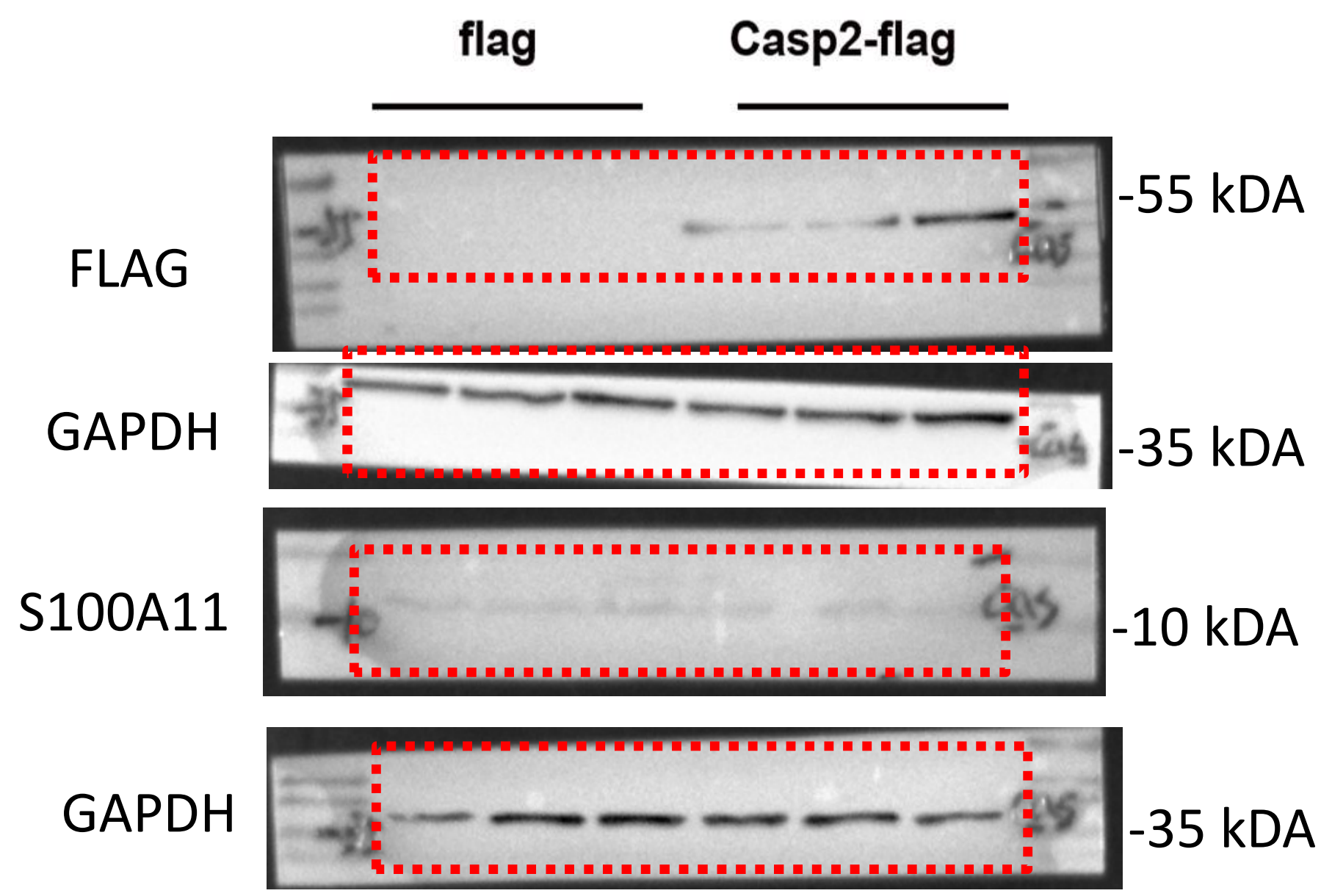

Full unedited blot/gel for Figure S4L

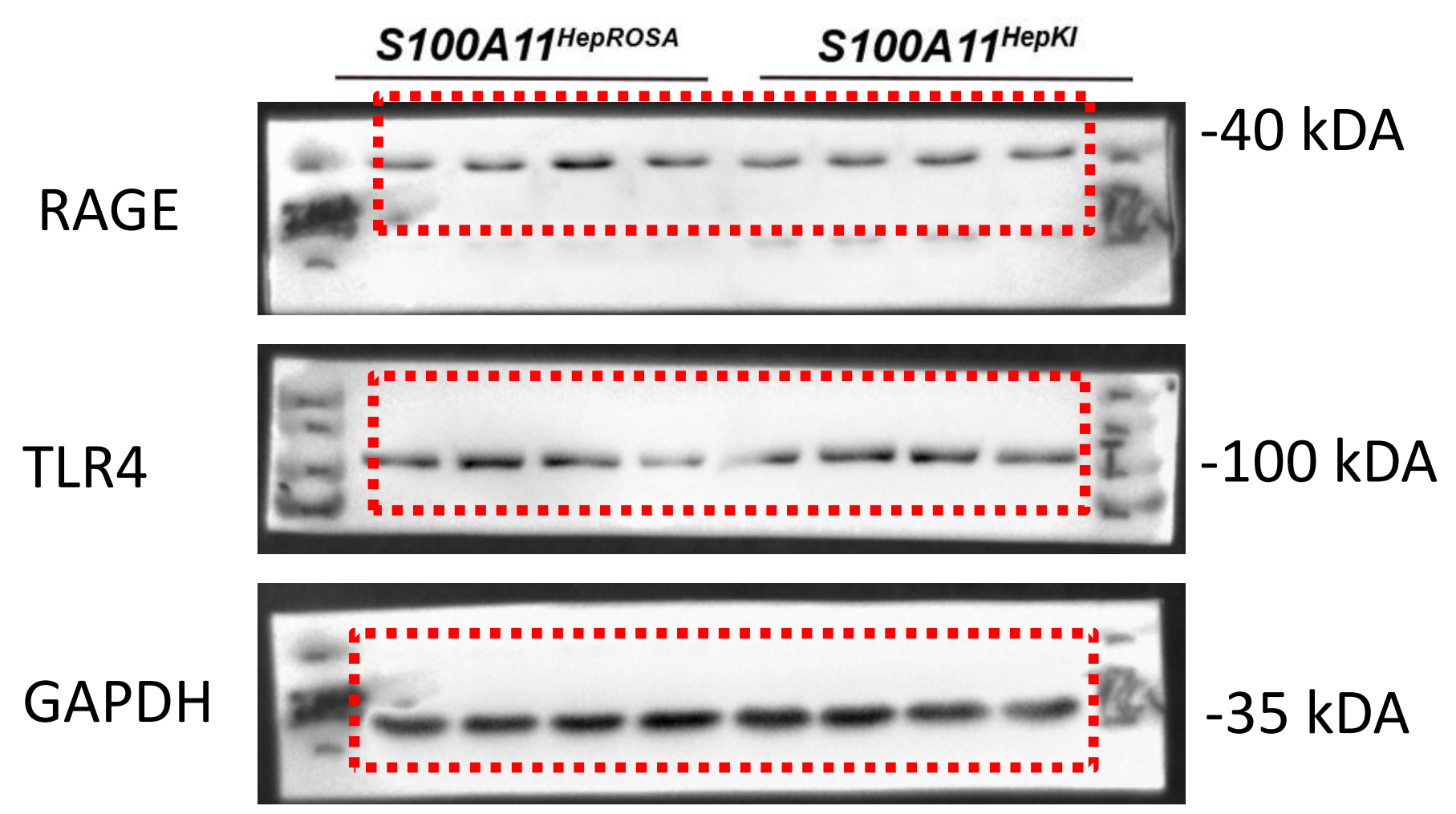

Full unedited blot/gel for Figure S4M

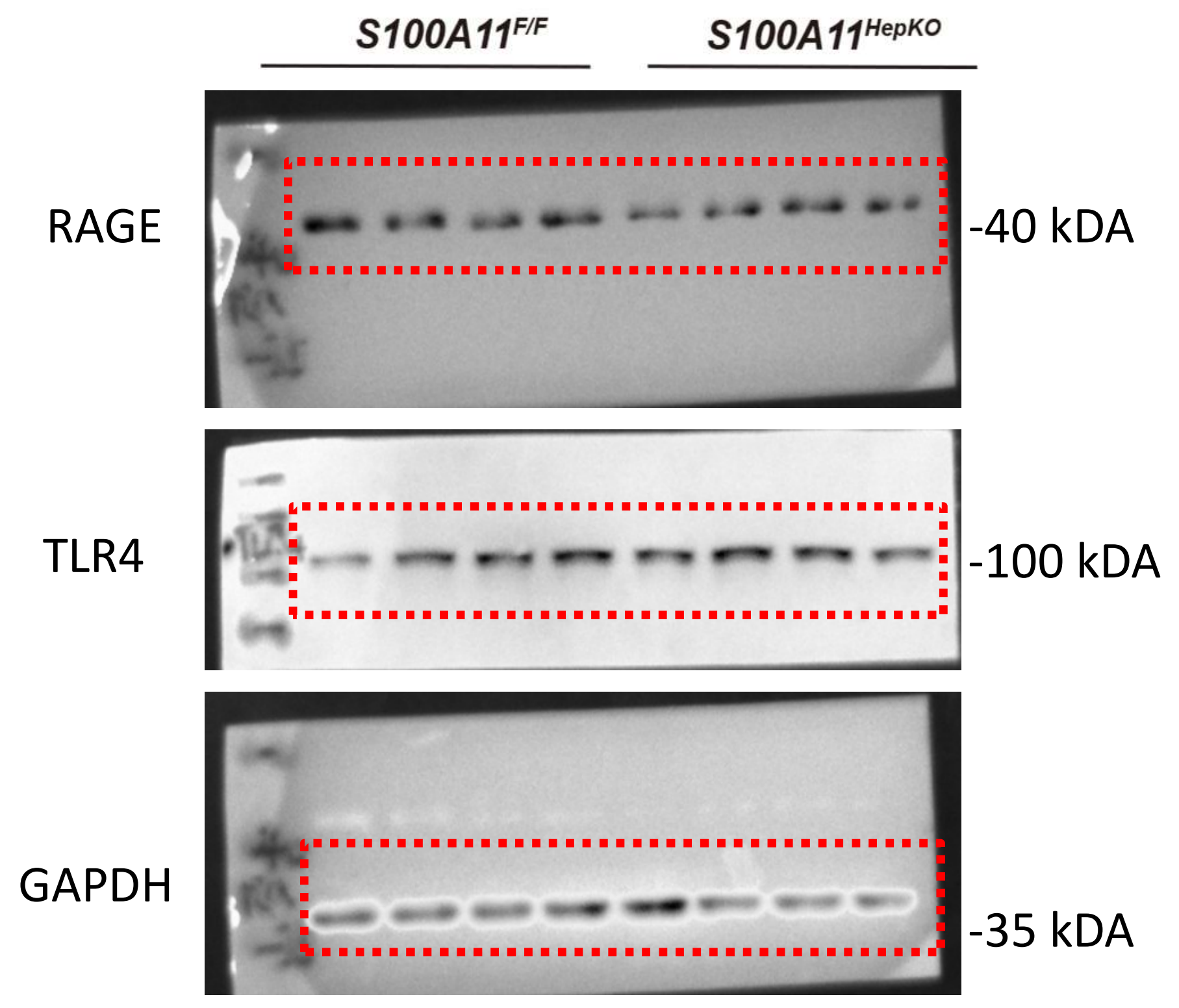

Full unedited blot/gel for Figure S5A

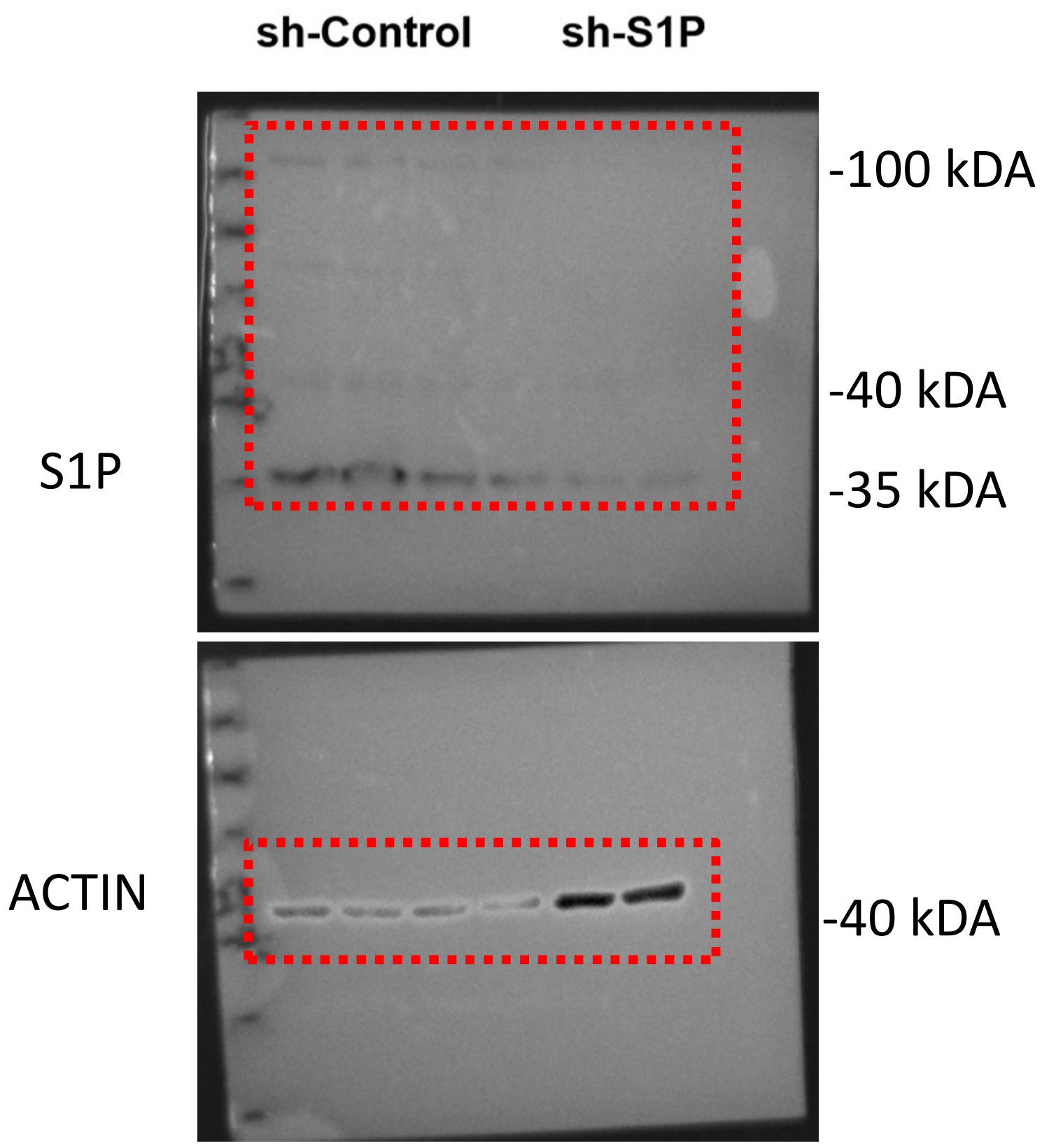

Full unedited blot/gel for Figure S5B

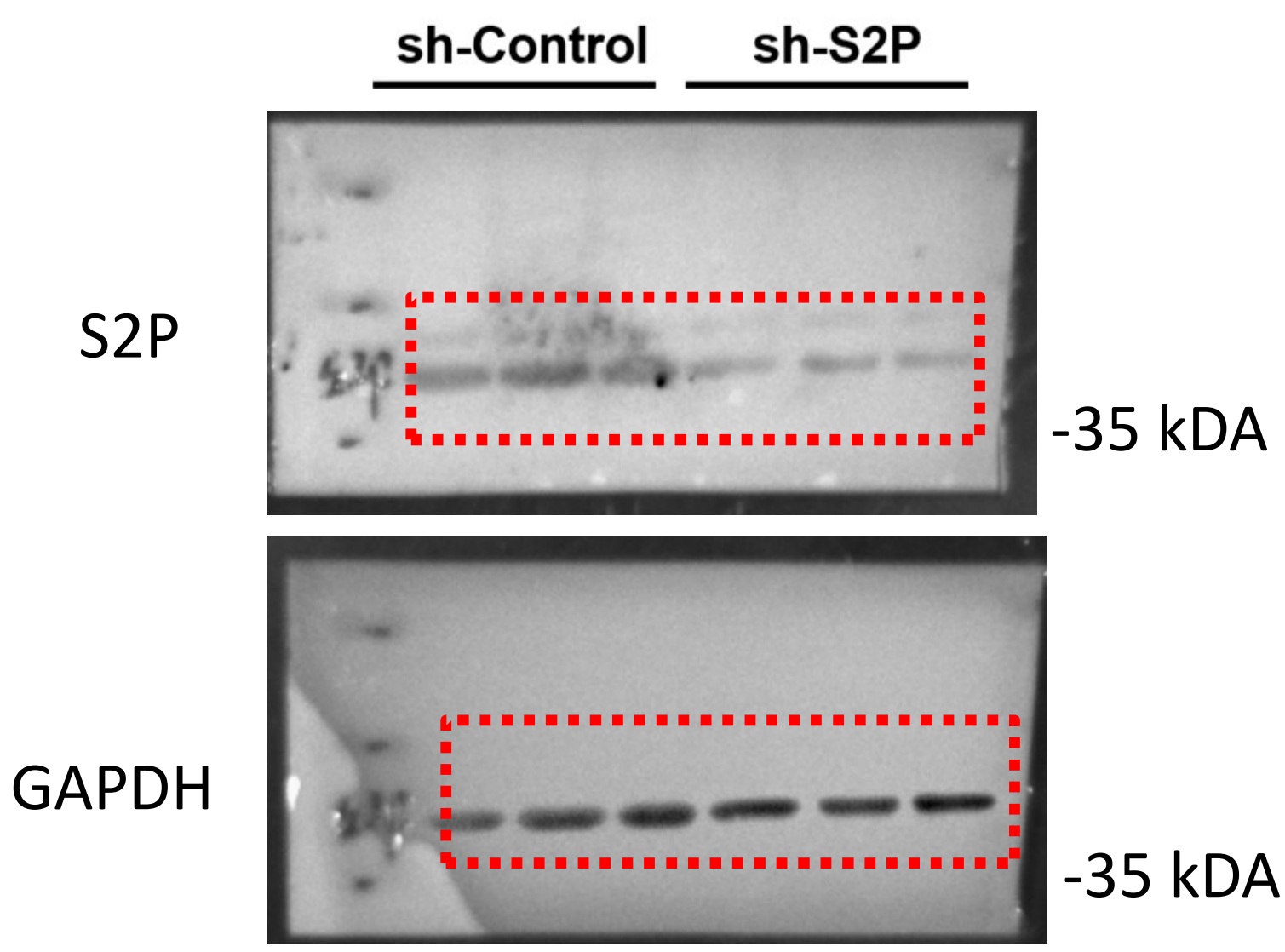

Full unedited blot/gel for Figure S5C

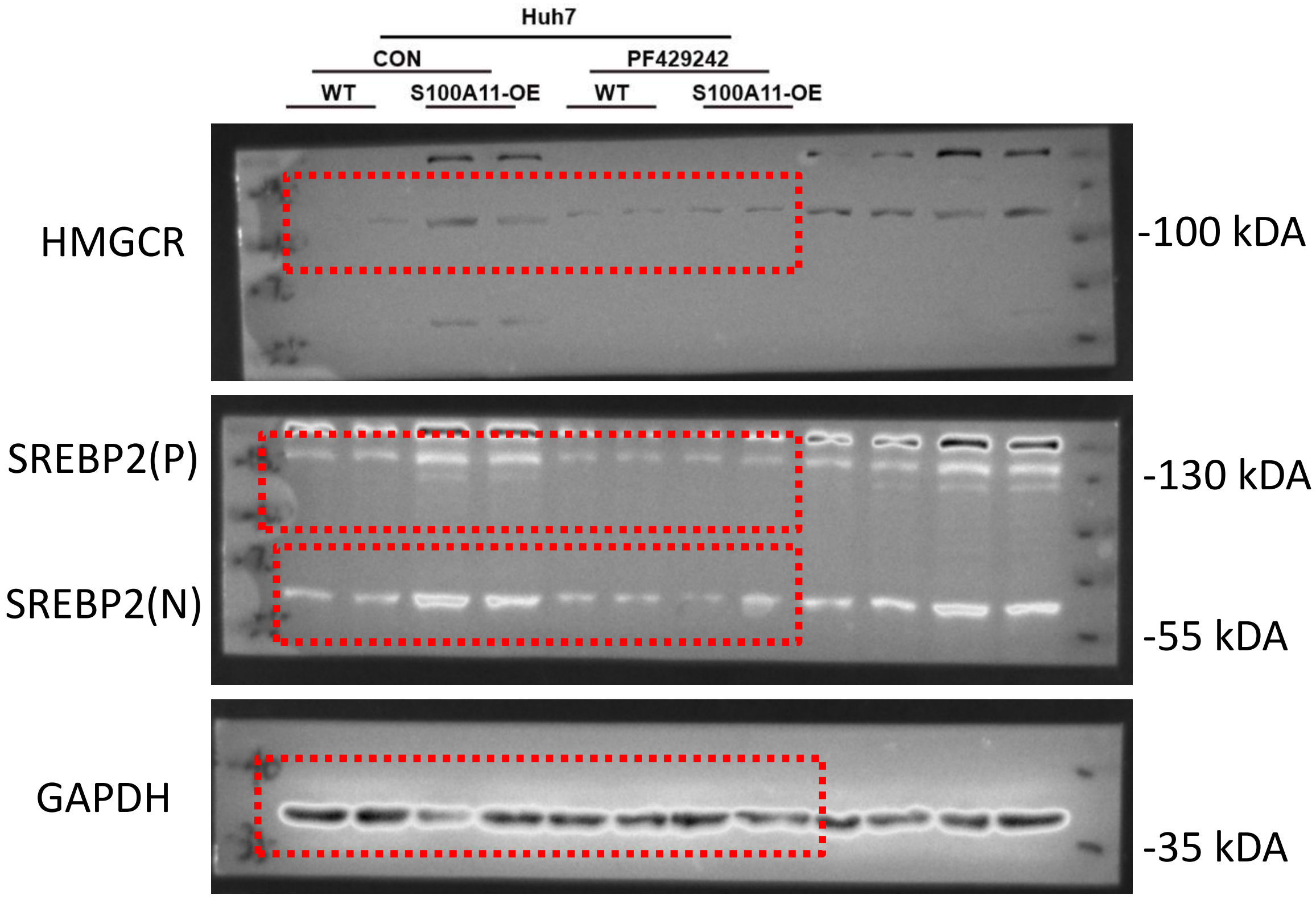

Full unedited blot/gel for Figure S5D

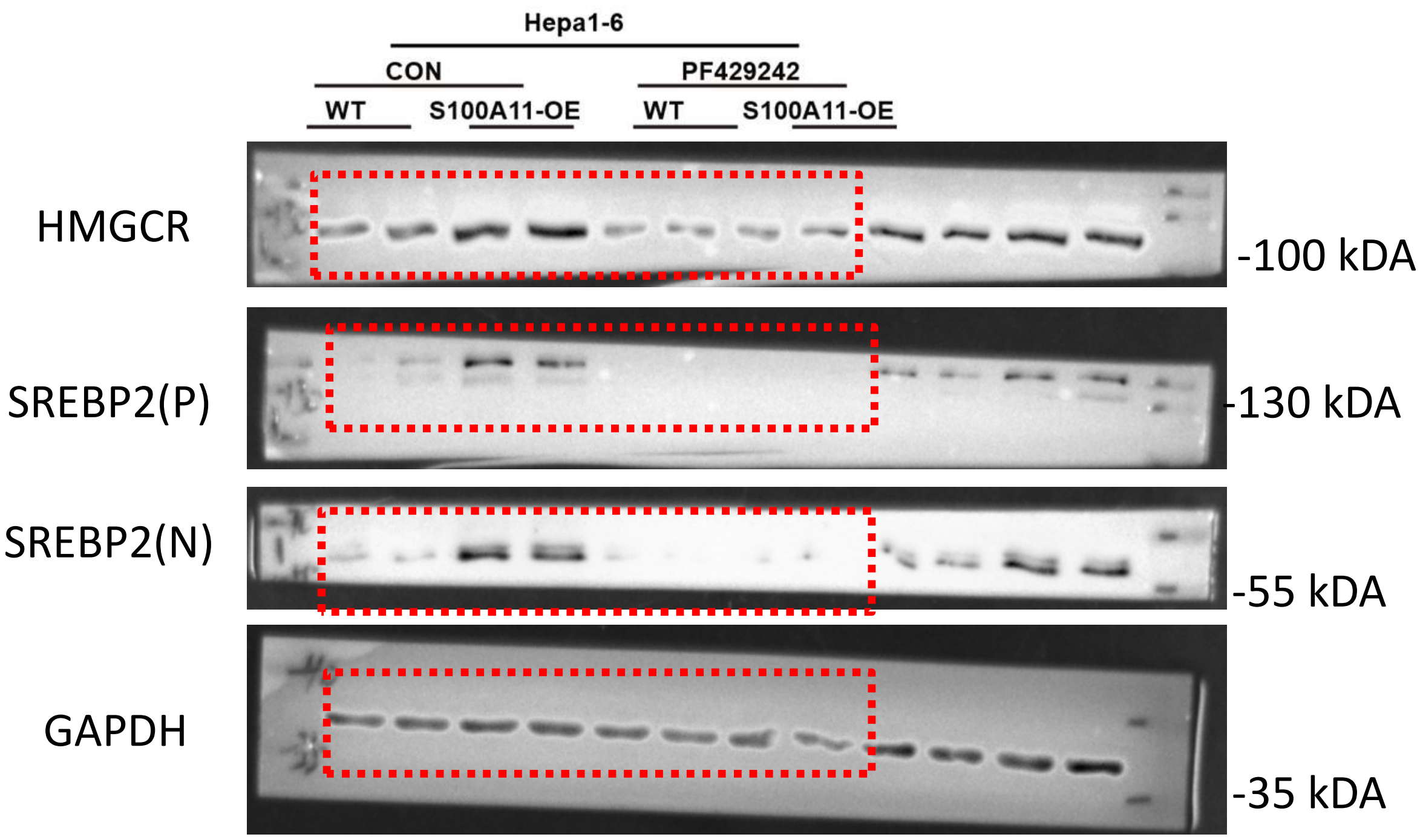

Full unedited blot/gel for Figure S5H

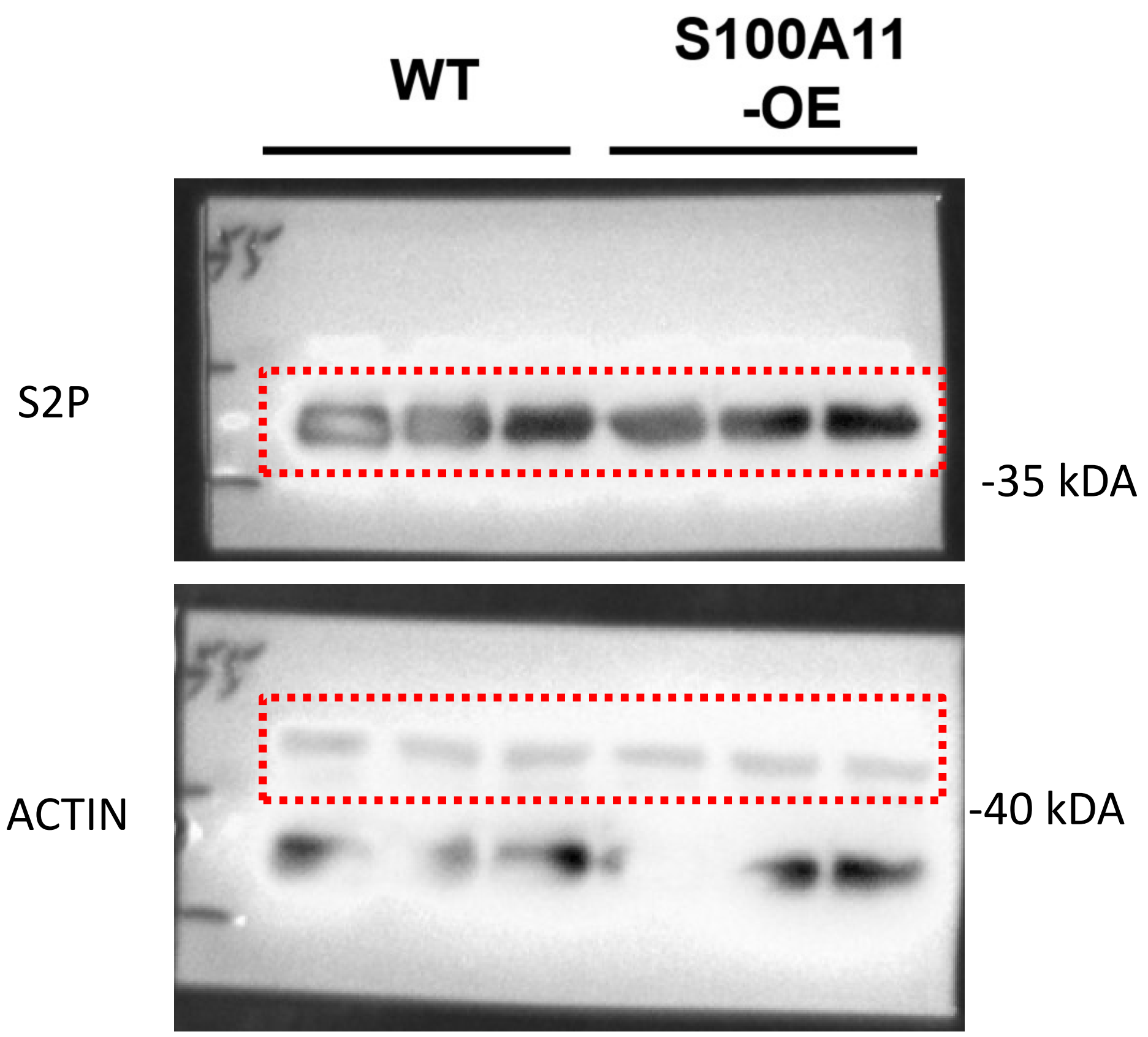

Full unedited blot/gel for Figure S5I

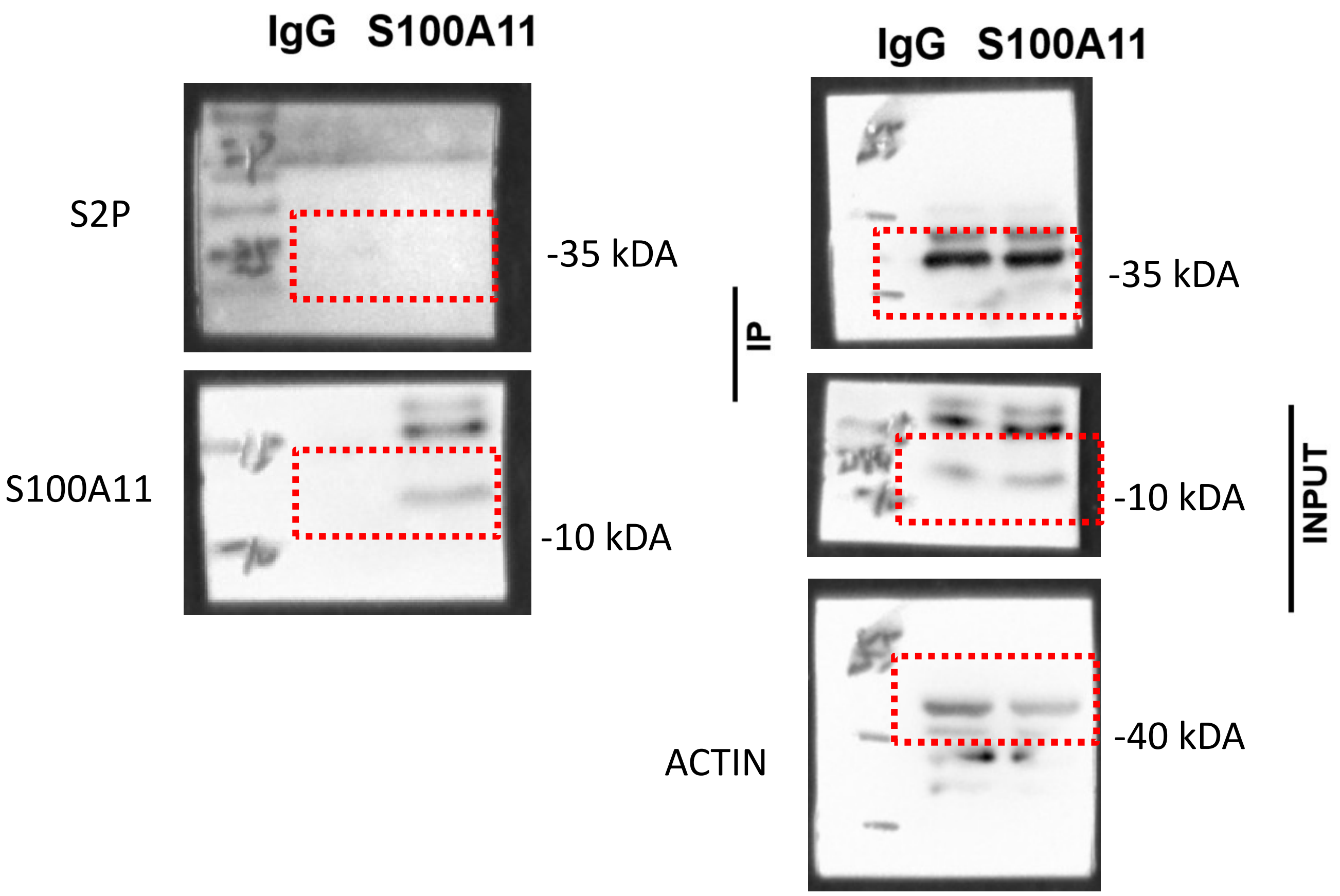

Full unedited blot/gel for Figure S6A

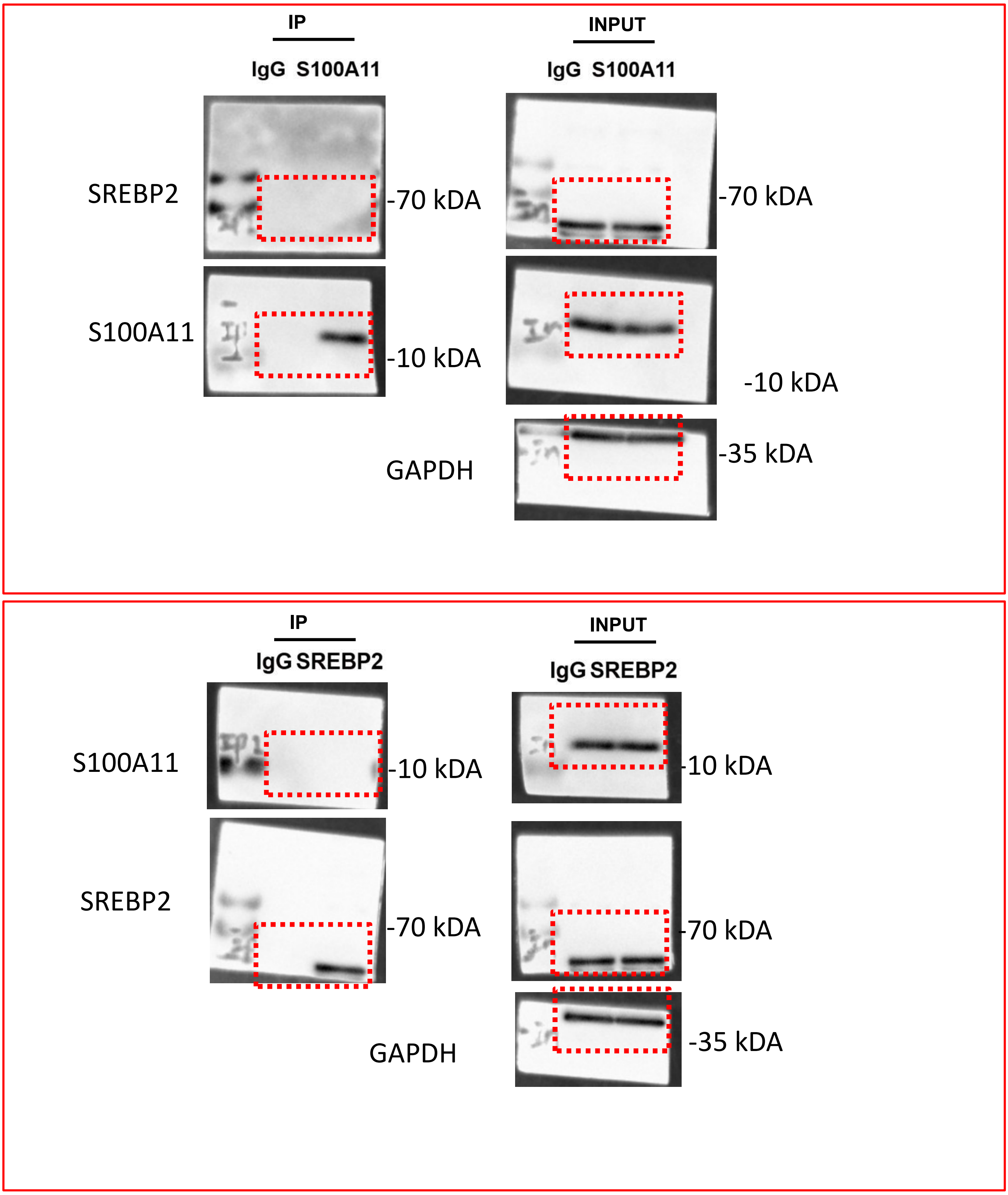

Full unedited blot/gel for Figure S6C

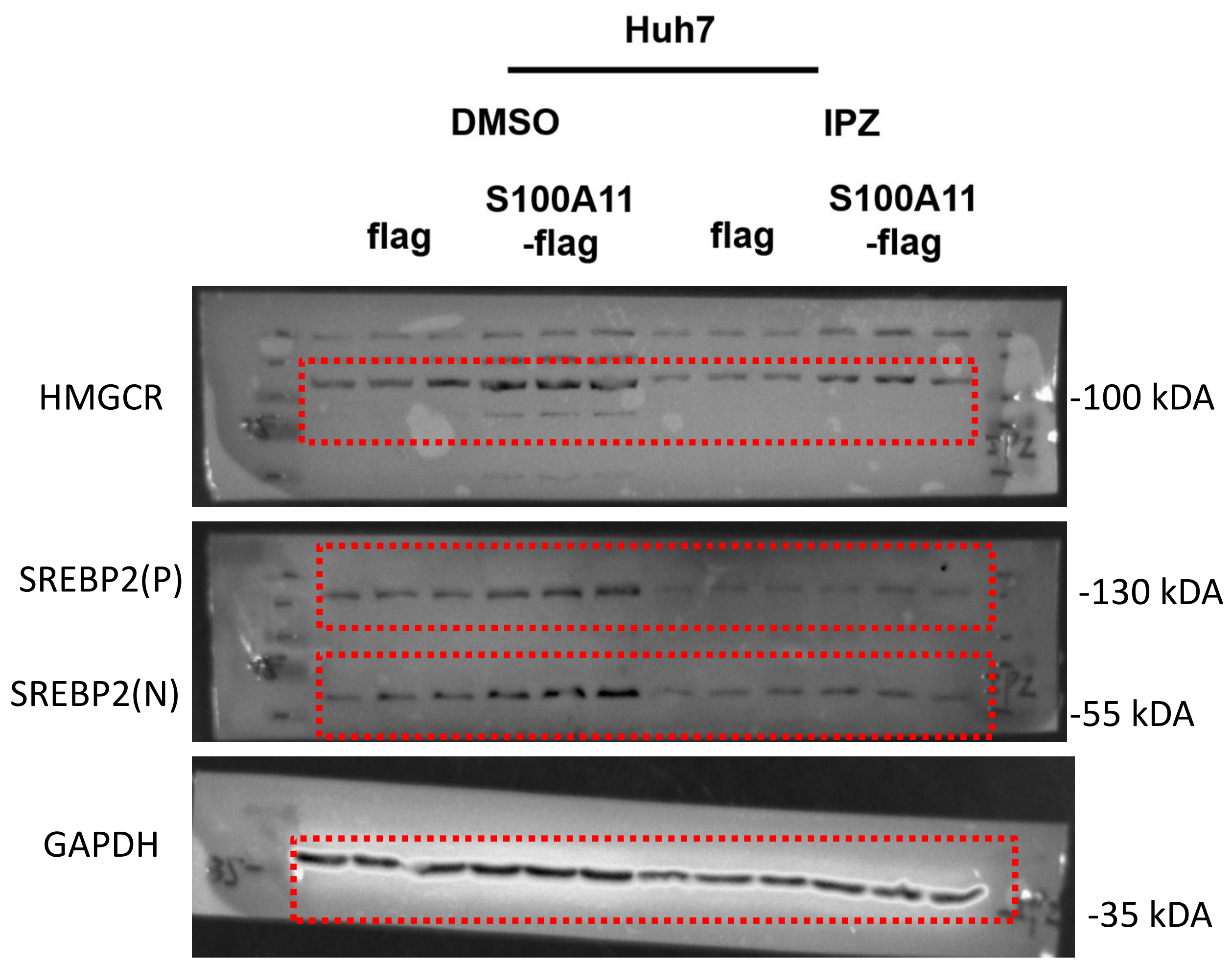

Full unedited blot/gel for Figure S6F

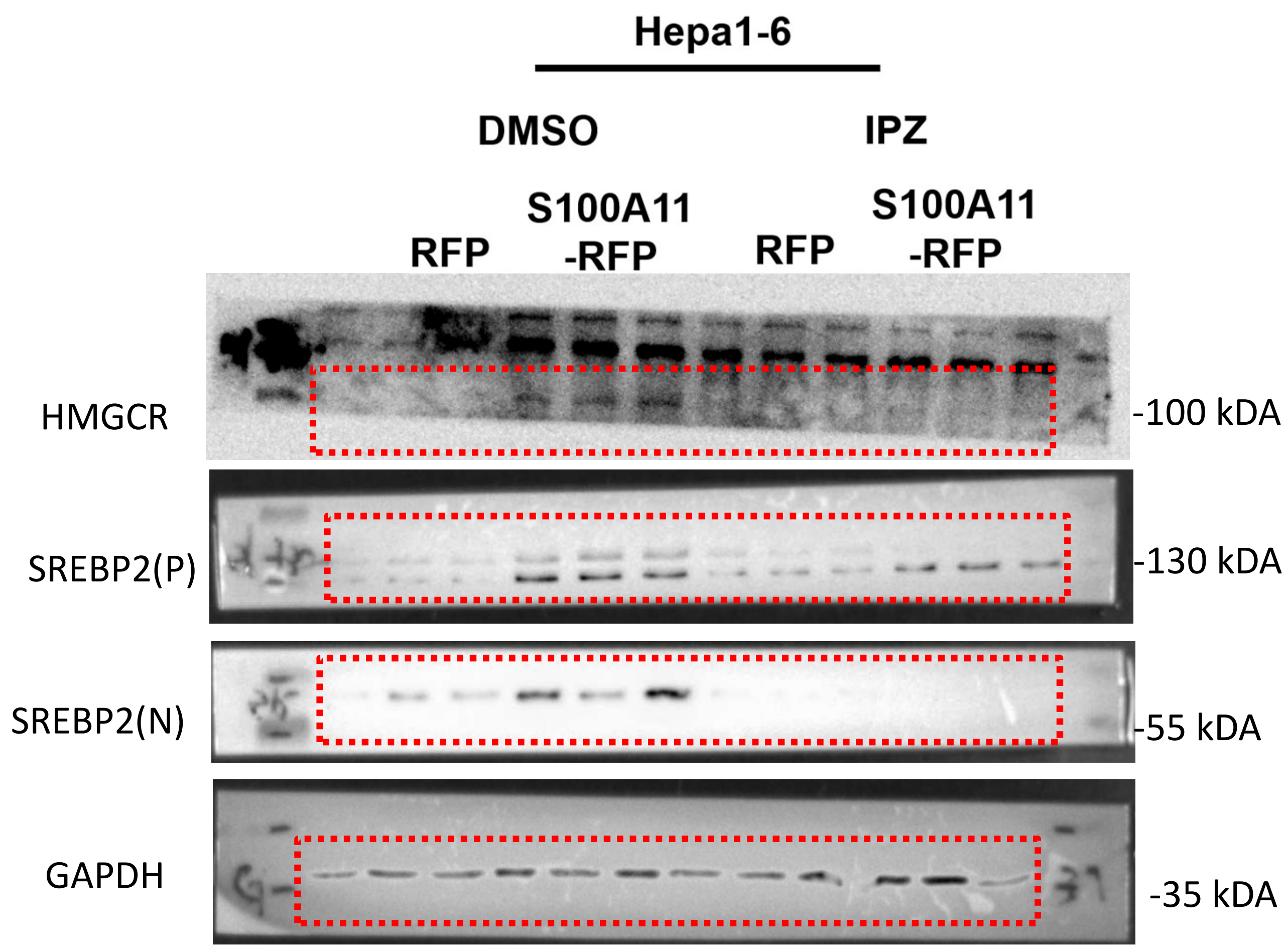

Full unedited blot/gel for Figure S7A

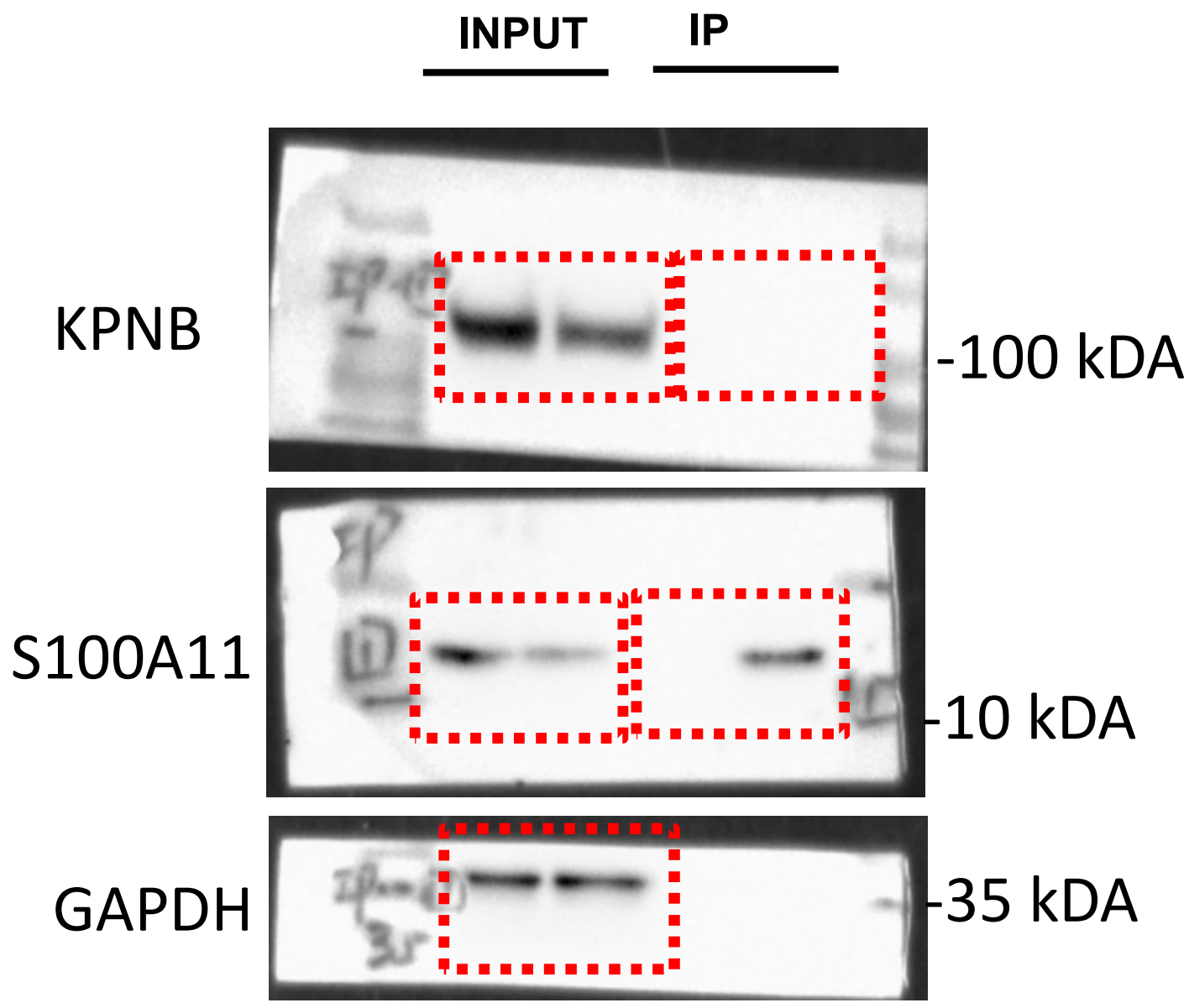

Full unedited blot/gel for Figure S7D

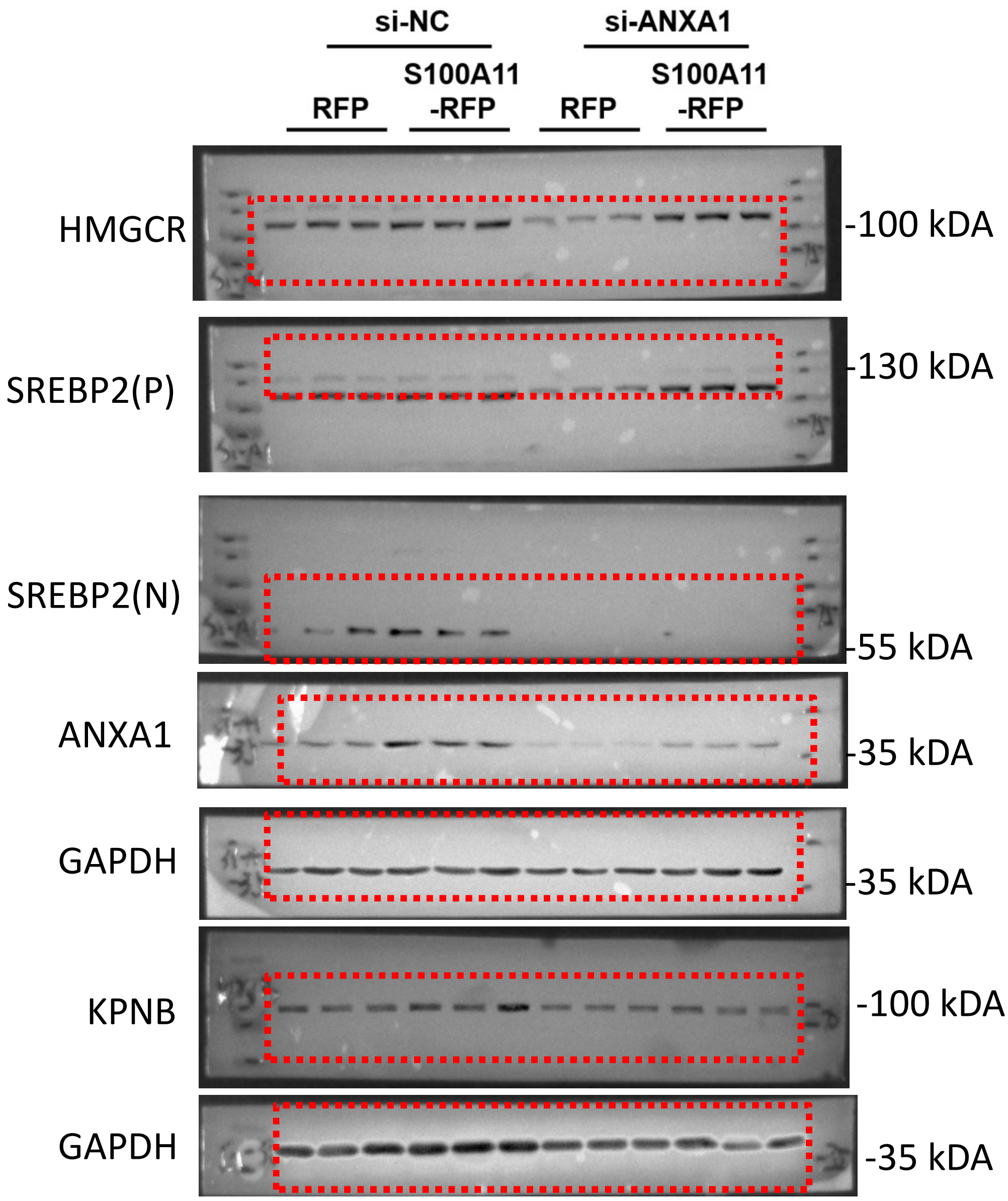

Full unedited blot/gel for Figure S7E

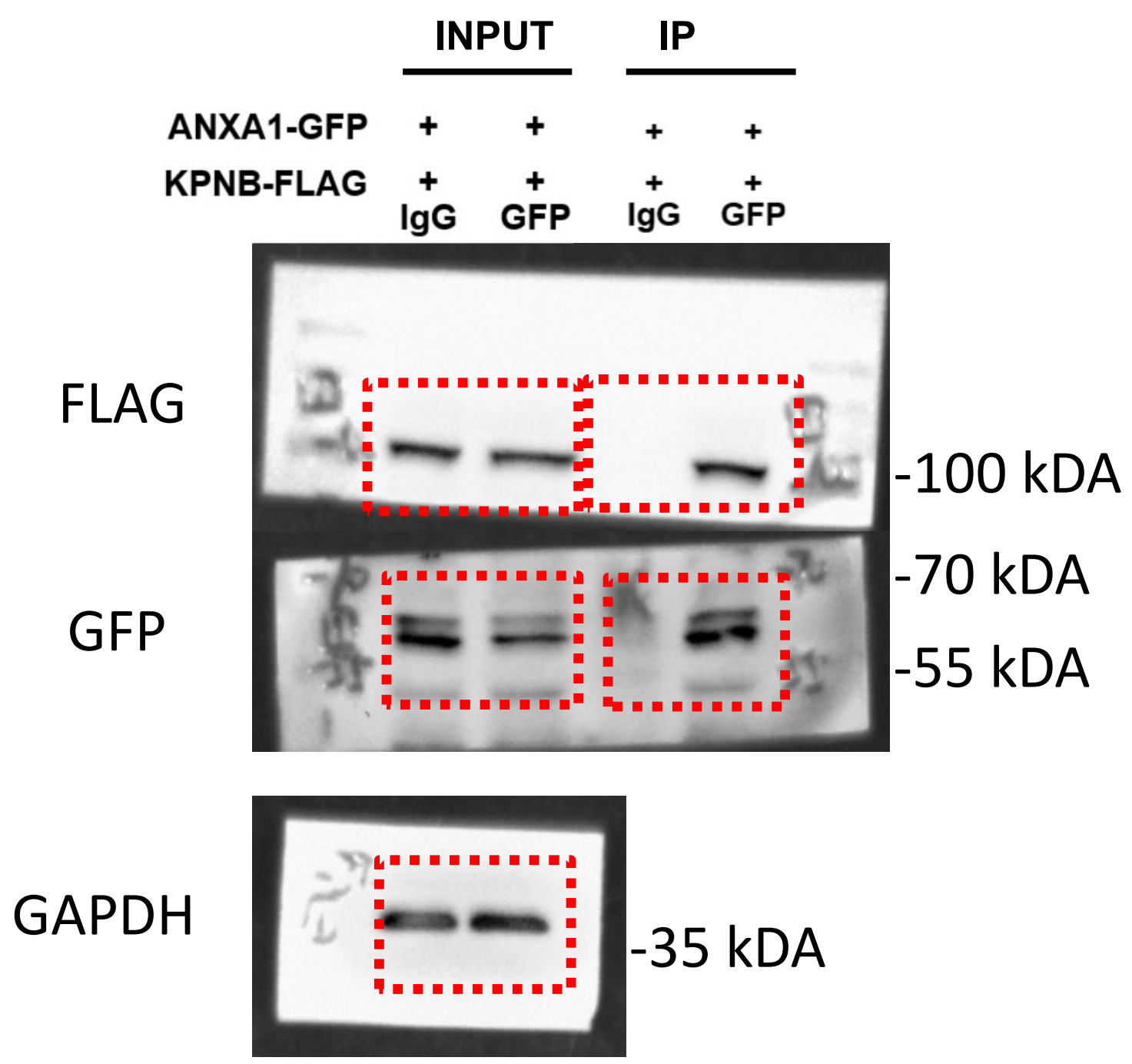

Full unedited blot/gel for Figure S7F

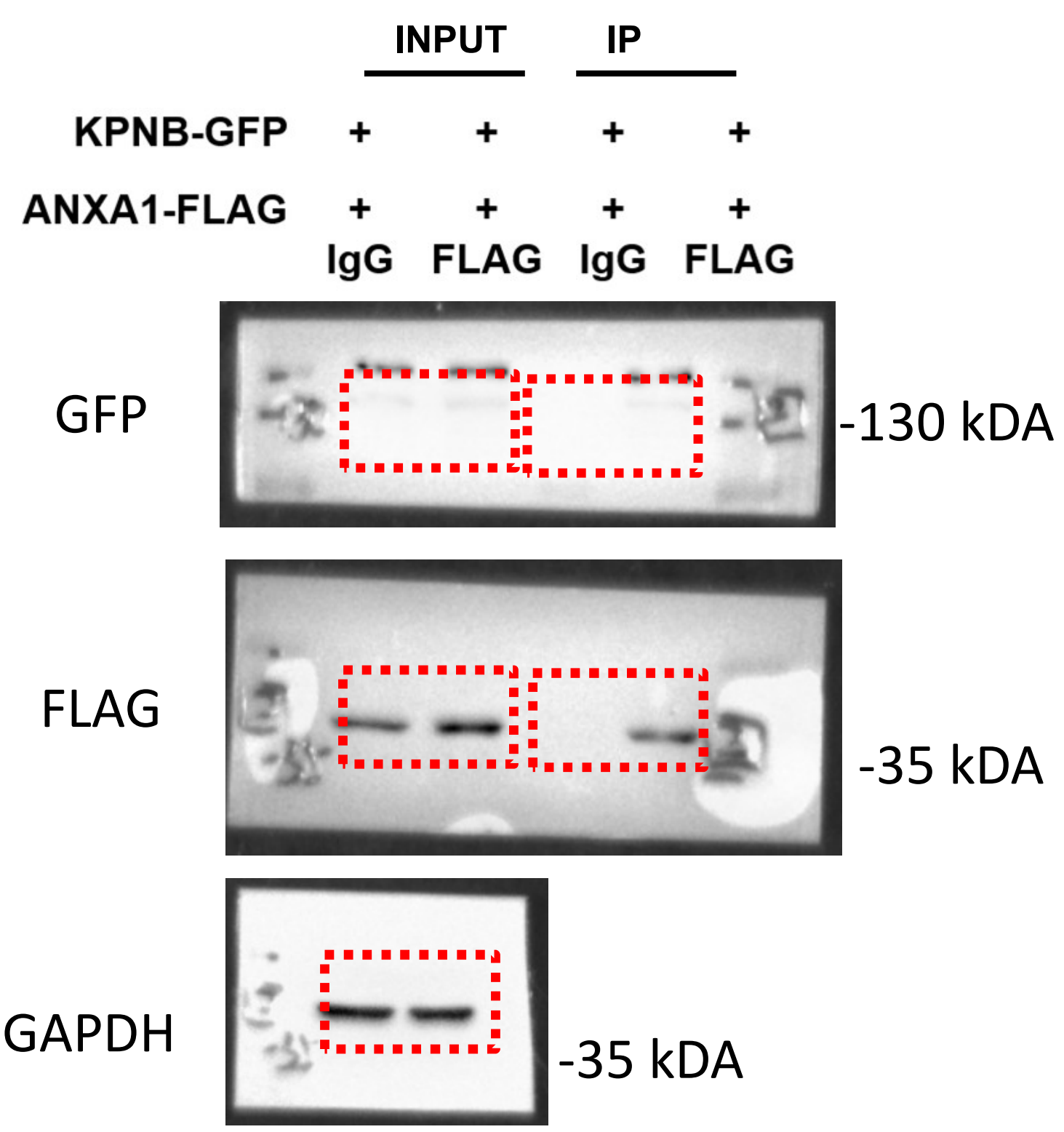

Supplement: Supplementary file 2 [file jci-136-201406-s022.pdf]
